# Unadjuvanted intranasal spike vaccine elicits protective mucosal immunity against sarbecoviruses

**DOI:** 10.1126/science.abo2523

**Published:** 2022-11-25

**Authors:** Tianyang Mao, Benjamin Israelow, Mario A. Peña-Hernández, Alexandra Suberi, Liqun Zhou, Sophia Luyten, Melanie Reschke, Huiping Dong, Robert J. Homer, W. Mark Saltzman, Akiko Iwasaki

**Affiliations:** ^1^ Department of Immunobiology, Yale University School of Medicine, New Haven, CT, USA.; ^2^ Section of Infectious Diseases, Department of Medicine, Yale University School of Medicine, New Haven, CT, USA.; ^3^ Department of Biomedical Engineering, Yale University, New Haven, CT, USA.; ^4^ Department of Molecular Biophysics and Biochemistry, Yale University, New Haven, CT, USA.; ^5^ Department of Pathology, Yale University School of Medicine, New Haven, CT, USA.; ^6^ Department of Chemical and Environmental Engineering, Yale University, New Haven, CT, USA.; ^7^ Department of Cellular and Molecular Physiology, Yale University, New Haven, CT, USA.; ^8^ Department of Dermatology, Yale University, New Haven, CT, USA.; ^9^ Howard Hughes Medical Institute, Chevy Chase, MD, USA.

## Abstract

The severe acute respiratory syndrome coronavirus 2 (SARS-CoV-2) pandemic has highlighted the need for vaccines that not only prevent disease but also prevent transmission. Parenteral vaccines induce robust systemic immunity but poor immunity at the respiratory mucosa. We developed a vaccine strategy that we call “prime and spike,” which leverages existing immunity generated by primary vaccination (prime) to elicit mucosal immune memory within the respiratory tract by using unadjuvanted intranasal spike boosters (spike). We show that prime and spike induces robust resident memory B and T cell responses, induces immunoglobulin A at the respiratory mucosa, boosts systemic immunity, and completely protects mice with partial immunity from lethal SARS-CoV-2 infection. Using divergent spike proteins, prime and spike enables the induction of cross-reactive immunity against sarbecoviruses.

## Prime and Spike passes the sniff test

Nearly 2 years after COVID-19 vaccines became widely available, a combination of waning vaccine-induced immunity and unabated viral mutations have resulted in reduced vaccine effectiveness. Mao *et al*. developed an alternative vaccine-boosting strategy they call “prime and spike” in animal models of COVID-19. After primary vaccination with a messenger RNA vaccine (“prime”), animals received an intranasal dose of unadjuvanted severe acute respiratory syndrome coronavirus 2 (SARS-CoV-2) spike protein (“spike”). This approach resulted in robust cellular and antibody-based immunity in the mucosa that protected animals as strongly and durably as a parenteral boost while also blocking viral transmission better. Spike could be administered in a variety of formulations and, if derived from SARS-CoV-1, could offer strong cross-protection against both viruses. —STS

## Abstract (structured)

**INTRODUCTION:**

During the first year of the severe acute respiratory syndrome coronavirus 2 (SARS-CoV-2) pandemic, multiple highly effective vaccines have been developed, using new technologies such as modified mRNA encapsulated in lipid nanoparticles and replication-deficient adenoviral vectors. Initial trials showed vaccine effectiveness >90% against symptomatic disease. Additional studies showed decreased infection rates of household contacts of vaccinated individuals, suggesting decreased rates of transmission. Unfortunately, waning immunity to vaccines and continued viral evolution with increasingly transmissible and immune-evasive variants have led to decreased vaccine effectiveness. Vaccines have also become much less effective at preventing transmission, which may be in part because of the poor induction of mucosal immunity within the respiratory tract.

**RATIONALE:**

Although the goal of vaccination has been to prevent individual morbidity and mortality, the evolution of SARS-CoV-2 throughout the pandemic has highlighted the need for vaccines that better prevent transmission. Parenteral vaccines induce robust systemic immunity that is protective against disease. However, they induce poor immunity within the upper respiratory mucosa, where viral transmission occurs. Preclinical studies of both SARS-CoV-2 and influenza virus have demonstrated that intranasal vaccination decreases viral shedding and transmission relative to parenteral vaccines. Despite these studies, there is only one currently approved respiratory mucosal vaccine, FluMist, which is a live cold-adapted influenza virus. Most current clinical trials of mucosally administered SARS-CoV-2 vaccines rely on either replication-deficient or attenuated viral vectors, the safety and efficacy of which have yet to be established. All of these strategies become less effective with the development of antivector immunity. Here, we instead leverage preexisting immunity to boost mucosal immunity by intranasally administering unadjuvanted spike protein or mRNA. This consequently avoids the use of viral vectors or adjuvants in the respiratory tract.

**RESULTS:**

We describe the preclinical development of an alternative vaccine strategy that we term “prime and spike” (P&S), which uses existing immunity generated by primary vaccination (prime) to elicit mucosal immune memory within the respiratory tract by using unadjuvanted intranasal spike booster. P&S elicits robust mucosal cellular and humoral memory responses, including the establishment of tissue-resident memory CD8^+^ T cells, CD4^+^ T cells, and B cells. Additionally, we found robust induction of mucosal immunoglobulin A (IgA) and IgG. Intranasal boosters can be delivered through distinct vaccine formulations, ranging from unadjuvanted trimeric recombinant spike proteins to spike-encoding mRNA encapsulated by immunosilent poly(amine-co-ester) (PACE) polymers. We found that an intranasal unadjuvanted spike booster can be administered months out from primary immunization and offers systemic neutralizing antibody responses comparable with that of mRNA–lipid nanoparticle (LNP) boost. P&S shows durability, leading to protection from lethal SCV2 challenge for as long as 118 days from vaccination. P&S is protective in hamsters and is superior to mRNA-LNP prime-boost at blocking transmission. Last, by using a divergent spike antigen from SARS-CoV-1, we demonstrate that P&S can generate mucosal immunity to SCV1 while also boosting systemic and mucosal neutralizing antibodies to SARS-CoV-2.

**CONCLUSION:**

SARS-CoV-2 will continue to evolve and become more immune evasive and transmissible. We will require boosting in human populations for the foreseeable future. The respiratory mucosa provides a formidable barrier against viral pathogens after P&S administration. Therefore, strengthening mucosal immunity through vaccination holds substantial promise for enhancing protection and mitigating transmission. As new variants emerge, it will be a vital tool in combating other respiratory pathogens and the next pandemic.

## Teaser

An intranasal spike protein boost generates broadly protective anti-sarbecovirus mucosal immunity in animals.

## Graphical abstract

**Figure:**
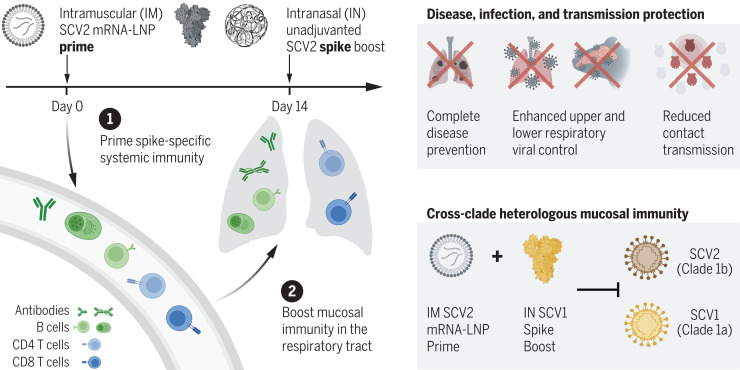
A parenteral prime-unadjuvanted mucosal boost vaccine that elicits mucosal immunity against sarbecoviruses. P&S converts systemic immunity generated by primary vaccination into local immunity in the respiratory mucosa. P&S affords protection against disease development, respiratory viral replication, and contact transmission after SARS-CoV-2 (SCV2) infection. Intranasal boosting by using a divergent spike protein from SARS-CoV-1 (SCV1) induces mucosal immunity against diverse sarbecovirus clades.

During the past 2 years of the severe acute respiratory syndrome coronavirus 2 (SARS-CoV-2) pandemic, there has been an unprecedented development of highly effective vaccines that use technologies including modified mRNA encapsulated in lipid nanoparticles (LNPs) and replication-deficient adenoviral vectors. Phase 3 clinical trials and subsequent postmarketing vaccine effectiveness studies initially showed >90% vaccine efficacy against symptomatic disease (*1*–*3*). Additionally, early transmission studies showed decreased rates of transmission in household members of vaccinated individuals (*4*, *5*). Unfortunately, recent studies have demonstrated decreasing vaccine effectiveness, starting 4 months after a second dose with mRNA-LNP–based regimens and earlier with other vaccines (*6*, *7*). Furthermore, continued viral evolution with increasing immune-evasive variants of concern (VOCs)—most recently, Omicron (B.1.529) and its sublineages—has also contributed to decreased vaccine effectiveness (*8*–*10*). With enhanced immune evasion and waning systemic immunity, current vaccines have become less effective at preventing viral transmission, which is likely worsened by increased viral transmissibility and their poor induction of mucosal immunity (*11*).

Currently approved SARS-CoV-2 vaccines rely on intramuscular (IM) administration, which induces high levels of circulating antibodies, memory B cells, and circulating effector CD4^+^ and CD8^+^ T cells in animal models and humans (*12*–*14*). However, parenteral vaccines do not induce high levels of potent antiviral immune memory at sites of infection, such as tissue-resident memory B cells (B_RM_ cells) and T cells (T_RM_ cells) as well as mucosal immunoglobulin G (IgG) and dimeric IgA (*15*–*17*). This contrasts with infection in humans and mice, in which CD8^+^ T_RM_ cells and mucosal IgA are robustly induced (*15*, *18*). Vaccines that target the respiratory mucosa could address the shortcomings of parenteral vaccination; recent assessments of intranasally delivered SARS-CoV-2 spike encoding adenoviral vectors have shown mucosal immunogenicity as well as protection and reduced viral shedding in mice, hamsters, and nonhuman primates (*19*–*23*).

Although primary respiratory administration of vaccines induces mucosal immunity, systemic priming followed by intranasal (IN) boosting results in similar systemic immunity to systemic prime-boost regimens but with enhanced mucosal immunity (*24*–*26*). Most examples of recombinant subunit vaccines administered either systemically or IN are coformulated with adjuvants to enhance immunogenicity. However, administration of vaccines to the respiratory tract in humans has proven difficult. There have been cases of IN adjuvanted inactivated vaccine for seasonal influenza that have led to Bell’s palsy, possibly caused by the specific toxin adjuvant mediating inflammation of neurons (*27*).

In the setting of nonprotective immunity from parenteral vaccination regimens, we assessed the immunogenicity and protection afforded by IN boosting with SARS-CoV-2 spike. We describe a vaccination strategy that uses systemic priming with mRNA-LNP followed by IN boosting with either unadjuvanted spike proteins or an immunosilent polyplex encapsulating spike mRNA.

## Results

### IN boosting with unadjuvanted SARS-CoV-2 spike induces mucosal humoral immunity

To assess the potential of IN unadjuvanted subunit vaccine boosting for the development of respiratory mucosal immunity, we decided to harness the strong systemic immunogenicity of mRNA-LNP. We additionally benefited from extensive SARS-CoV-2 spike engineering by using HexaPro, which has been shown to substantially enhance immunogenicity and increase protein stability (*28*).

**Fig. 1.**
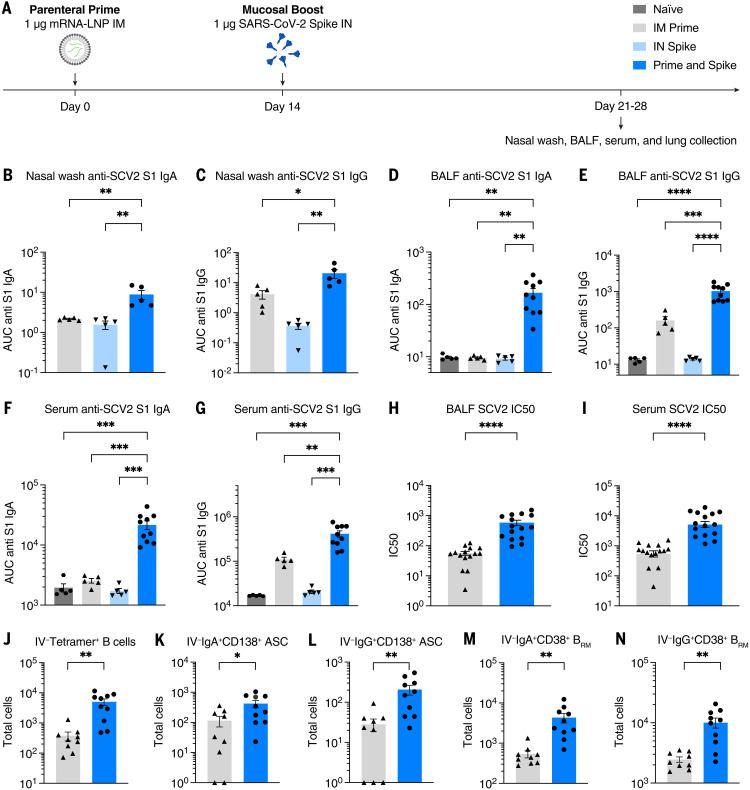
IN boosting with stabilized SARS-CoV-2 spike induces mucosal humoral memory. (**A**) Experimental schema. Mice were intramuscularly immunized with 1 μg of mRNA-LNPs encoding full-length SARS-CoV-2 (SCV2) spike protein (Pfizer/BioNTech BNT162b2), followed by IN immunization with 1 μg of prefusion-stabilized (Hexapro), trimeric, recombinant SCV2 spike protein 14 days after mRNA-LNP immunization. Fourteen days after IN boost, serum, BALF, and nasal washes were collected to assess binding and neutralizing antibody responses. Lung tissues were collected for extravascular B cell analysis. (**B** to **G**) Measurement of SCV2 spike S1 subunit–specific (B) nasal wash IgA, (C) nasal wash IgG, (D) BALF IgA, (E) BALF IgG, (F) serum IgA, and (G) serum IgG in naïve mice, mice immunized with mRNA-LNP IM (IM Prime), mice immunized with the spike protein IN (IN Spike), or mice IM primed and IN boosted with spike (P&S). (**H** to **K**) Measurement of neutralization titer against SCV2 spike–pseudotyped vesicular stomatitis virus (VSV) in (H) BALF and (I) serum. (**J** to **N**) Using CD45 IV labeling, various extravascular (IV labeling antibody negative) B cell subsets were measured, including RBD tetramer-binding B cells, IgA^+^ B_RM_ cells, IgG^+^ B_RM_ cells, IgA^+^ ASCs, and IgG^+^ ASC in lung tissues from IM Prime or P&S mice. Mean ± SEM. Statistical significance was calculated by means of [(B) to (G)] one-way analysis of variance (ANOVA) or [(H) to (N)] Student’s *t* test; *P ≤ 0.05, **P ≤ 0.01, ***P ≤ 0.001, ****P ≤ 0.0001. Individual data points are represented and are pooled from two or three independent experiments.

We vaccinated K18-hACE2 (mice) with mRNA-LNP (Pfizer/BioNTech BNT162b2) by means of IM injection (prime), followed 14 days later with IN administration of recombinant unadjuvanted spike protein [prime and spike (P&S)]. Mice were euthanized at days 21 or 28 and assessed for mucosal humoral immunity (Fig. 1A).

First, we assessed anti–SARS-CoV-2 spike IgG and IgA in nasal wash (Fig. 1, B and C), bronchoalveolar lavage fluid (BALF) (Fig. 1, D and E), and serum (Fig. 1, F and G). Only mice that received P&S developed high levels of anti–SARS-CoV-2 IgA and IgG in the nasal wash and BALF. Neither IM prime nor IN spike alone was sufficient to develop mucosal antibodies. In the serum, prime alone was sufficient to induce low levels of IgA and IgG. By contrast, P&S led to significant systemic boosting of both anti-spike IgA and IgG. These increases in antibody levels correlated with increases in neutralization titers both in BALF (Fig. 1H) and serum (Fig. 1I). Thus, a single-dose unadjuvanted IN spike alone is not immunogenic, and the induction of a potent mucosal and systemic antibody response by unadjuvanted spike requires prior systemic priming, in this case with mRNA-LNP.

B_RM_ cells in the lungs assist in rapid recall response of antibody-secreting plasma cells upon secondary heterologous challenge in influenza models and may be an important local immune effector in protecting against SARS-CoV-2 (*29*). Using intravenous (IV) CD45 labeling to differentiate circulating immune cells within lung tissue combined with B cell tetramers specific for the receptor binding domain (RBD) of the spike protein, we found that P&S leads to increased antigen-specific B cells within lung tissue (IV-CD45^−^B220^+^CD19^+^tetramer^+^) (Fig. 1J). We also examined the polyclonal tissue response, which likely represents a more complete set of spike-specific B cells within the lungs. We found increases in class-switched antibody-secreting cells (ASCs) (IV-CD45^−^CD19^+/−^CD138^+^) in lung tissue expressing IgA or IgG (Fig. 1, K and L), and we found increased class-switched B_RM_ cells (IV-CD45^−^B220^+^CD19^+^IgD^−^IgM^−^CD38^+^) expressing IgA or IgG (Fig. 1, M and N). Thus, P&S elicits local B cell responses in the lung.

### Prime and spike induces mucosal T cell immunity

**Fig. 2.**
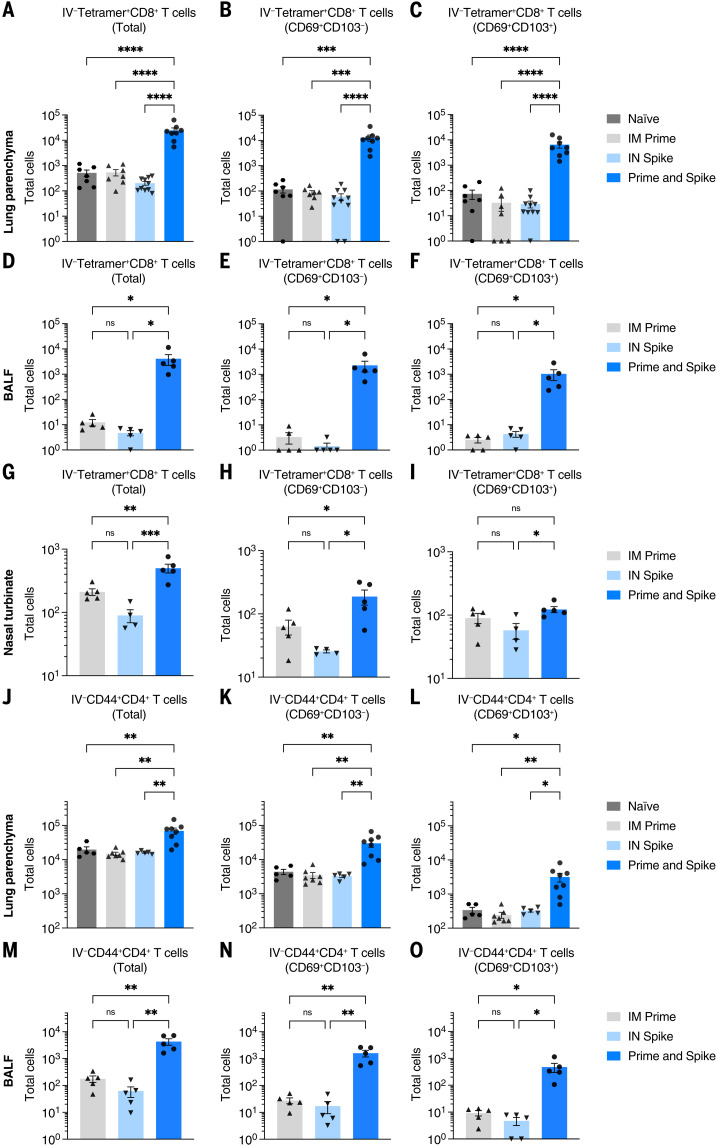
IN boosting with stabilized SARS-CoV-2 spike induces mucosal T cell memory. K18-hACE2 mice were intramuscularly primed with 1 μg mRNA-LNP and 14 days later intranasally boosted with 1 μg SCV2 spike. Lung tissues, BALF, and nasal turbinates were collected for extravascular T cell analysis. Lung tissues were collected 14 days after boost, whereas BALF and nasal turbinates were obtained 7 days after boost. (**A** to **I**) Extravascular CD8 T cell responses. Shown are quantification of SCV2 spike–specific tetramer^+^ CD8 T cells, CD69^+^CD103^−^tetramer^+^ CD8 T cells, or CD69^+^CD103^+^tetramer^+^ CD8 T cells in [(A) to (C)] lung tissues, [(D) to (F)] BALF, or [(G) to (I)] nasal turbinates from naïve, IM prime, IN spike, or P&S mice. (**J** to **O**) Extravascular CD4 T cell responses. Shown are quantification of activated polyclonal CD4 T cells, CD69^+^CD103^−^ CD4 T cells, or CD69^+^CD103^+^ CD4 T cells in [(J) to (L)] lung tissues or [(M) to (O)] BALF from naïve, IM prime, IN spike, or P&S mice. Mean ± SEM. Statistical significance was calculated by means of [(B) to (O)] one-way ANOVA followed by Tukey’s correction; *P ≤ 0.05, **P ≤ 0.01, ***P ≤ 0.001, ****P ≤ 0.0001. Individual data points are represented and are pooled from two or three independent experiments.

Given that P&S induced respiratory mucosal humoral memory, we next assessed the induction of lung T_RM_ cells. Although adjuvant-free subunit vaccines have not traditionally been potent inducers of antigen-specific T cell responses, we hypothesized that the immune memory generated by mRNA-LNP priming would enable subunit-mediated T cell–boosting responses. To identify spike-specific CD8^+^ T cells, we used major histocompatibility complex (MHC) class I tetramer S_539-546_ (VNFNFNGL). There was a significant induction of IV-CD45^−^tetramer^+^ CD8^+^ T cells, which expressed canonical markers of T_RM_ cells including CD69^+^ and CD103^+^, within lung tissue (Fig. 2, A to C), BALF (Fig. 2, D to F), and the nasal turbinate (Fig. 2, G to I). Moreover, there were significant increases in antigen-experienced CD4^+^ T cells (IV-CD45^−^CD44^+^CD4^+^), many of which also expressed CD69^+^ and CD103^+^ both within lung tissue (Fig. 2, J to L) and in the BALF (Fig. 2, M to O). Thus, P&S mediates expansion of lung parenchyma and airway CD8^+^ T_RM_ and CD4^+^ T_RM_ cells.

### Host genotype, boosting interval, and IN volume have little effect on P&S

To assess whether mouse genotype, boosting interval, or boosting volume affected immunity induced by P&S, we compared mucosal CD8^+^ T cell and antibody responses after P&S under varying conditions, including in K18-hACE2 versus C57B6/J (B6J) mice, 2-week versus 4-week boosting intervals, and 25- versus 50-μl IN inoculations (fig. S1A). Antigen-specific lung CD8^+^ T_RM_ cells (fig. S1, B to D), BALF IgA and IgG (fig. S1, E and F), serum IgA and IgG (fig. S1, G and H), and serum neutralizing responses (fig. S1I) were similar among all groups and significantly higher than responses elicited by prime alone. These results support the robustness of P&S because multiple experimental variables can be modified without affecting overall immune responses.

### Delayed-interval P&S induces mucosal immunity

We wondered whether boosting at an increased interval would affect P&S responses. To test this, mice received IM mRNA-LNP and were boosted with IN spike 84 days later. Humoral and cellular mucosal immune responses on days 91 and 140 were sampled (fig. S2A). Delayed P&S was sufficient to induce CD8^+^ T_RM_ cells for at least 56 days (fig. S2, B to D). Polyclonal CD4^+^ T_RM_ cells were induced early at 7 days after boost. However, their numbers appeared to wane by 56 days (fig. S2, E to G). Delayed P&S also resulted in enhanced mucosal IgA and IgG in BALF (fig. S2, H and I) and serum IgA and IgG (fig. S2, J and K) at 56 days after boost. Thus, P&S administered even up to 3 months after priming elicits durable mucosal humoral and cellular immune responses.

### IN delivery of mRNA polyplexes also mediates mucosal boosting

Poly(amine-co-ester)s (PACEs) are biodegradable terpolymers that have been developed to encapsulate and deliver nucleic acids such as mRNA to specified tissues in vivo (*30*). Recent studies have shown that mRNA-LNP delivered to the respiratory tract is lethal in a dose-dependent manner in mice (*31*). By contrast, PACE materials have been developed to be relatively immunologically silent, enabling administration to locations more susceptible to immunopathology such as the respiratory tract. Chemically modifying PACE with polyethylene glycol dramatically improves in vivo lung delivery (*32*). To assess the utility of PACE encapsulating mRNA encoding spike protein as an IN booster, mRNA was extracted from BNT162b2 and encapsulated in PACE. Mice were primed intramuscularly with mRNA-LNP and boosted with IN spike mRNA encapsulated in PACE (PACE-spike). Additional control groups included PACE-spike only and IM prime + extracted mRNA (naked mRNA) (fig. S3A). Similar to what we found with P&S, prime and PACE-spike induced antigen-specific CD8^+^ T_RM_ cells (IV-CD45^−^tetramer^+^CD69^+^CD103^+^) (fig. S3, B to D). Additionally, PACE-spike–boosted mice developed high levels of BALF IgA. Levels of BALF IgG and serum IgA and IgG were similar to IM prime alone (fig. S3, E to H). IM prime followed by IN naked mRNA was unable to induce mucosal or systemic immune responses above that of IM prime alone, indicating that mRNA encapsulation by PACE was required for mucosal boosting. Additionally, a single dose of IN PACE-spike alone was insufficient to elicit any detectable mucosal or systemic antibody response at this dose.

### IN spike or IN PACE-spike boosts suboptimal prime to protect against lethal SARS-CoV-2 challenge

Although current vaccines were initially extremely effective at eliciting protective immunity, waning antibody levels and immune evasion will necessitate boosters for the foreseeable future. The best approach to boosting remains an open question. To test whether IN administration would provide an alternative protective boost, we used a low-dose (LD) 0.05 μg of mRNA-LNP vaccine to mimic nonprotective immunity. We have previously shown that this dose is insufficient to protect from SARS-CoV-2 challenge despite inducing systemic antibody responses (*15*). Mice intramuscularly primed with LD mRNA-LNP and boosted with IN spike developed antigen-specific lung CD8^+^ T_RM_ cells and IgA and IgG in the BALF at 42 days after boost (fig. S4). Thus, low levels of immune memory allow for effective mucosal boosting of humoral and cellular responses by unadjuvanted IN spike.

**Fig. 3.**
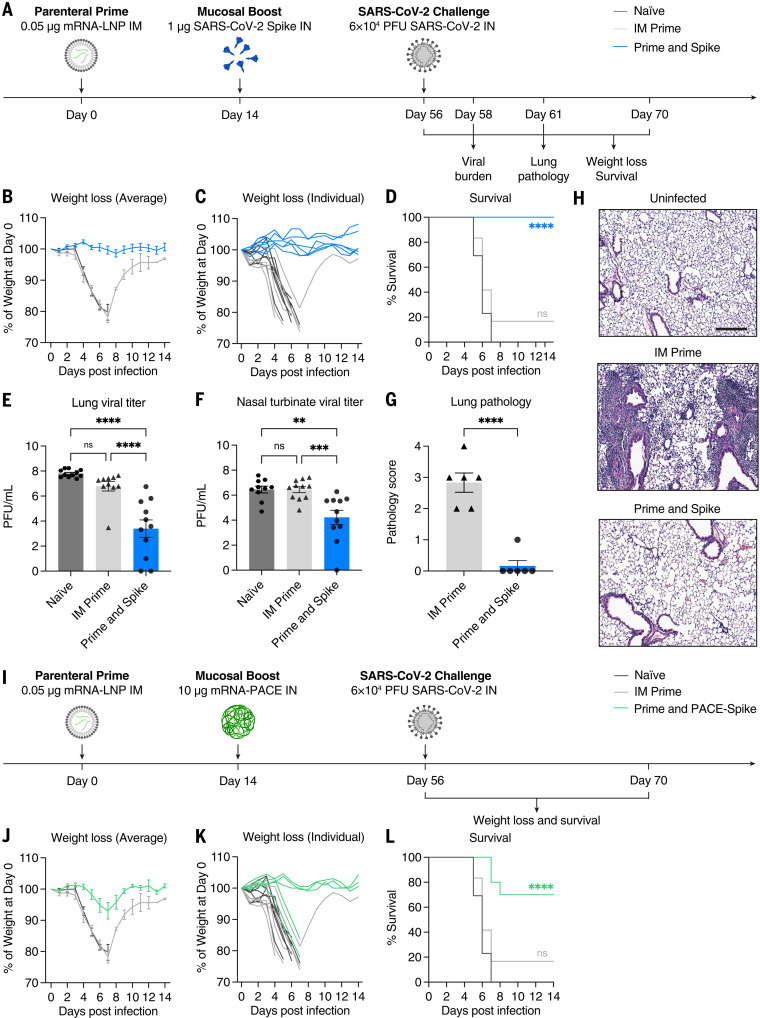
IN SARS-CoV-2 spike boosting protects against COVID-19–like disease. (**A**) Experimental schema. K18-hACE2 mice were intramuscularly primed with 0.05 μg of mRNA-LNP and intranasally boosted with 1 μg of spike 14 days after IM prime. Six weeks after boost, mice were challenged with 6 × 10^4^ PFU SCV2 (2019n-CoV/USA_WA1/2020). The first cohort was used to evaluate weight loss and survival up to 14 days after infection. The second cohort was used to collect lung and nasal turbinate tissues 2 days after infection for viral titer measurement. The third cohort was used to collect lung tissues 5 days after infection for histological assessment. (**B** to **D**) Weight loss and survival of naïve, IM prime, or P&S mice from 1 to 14 days after infection. (**E** to **F**) Measurement of infectious virus titer in lung and nasal turbinate tissues at 2 days after infection by means of plaque assay. (**G**) Pathology score of lung sections at 5 days after infection by means of H&E staining. (**H**) Representative H&E staining results from uninfected, IM prime, or P&S mice. Scale bar, 250 μm. Sections are representative of multiple sections from at least five mice per group. (**I**) Experimental schema. K18-hACE2 mice were intramuscularly primed with 0.05 μg of mRNA-LNP and intranasally boosted with 10 μg of mRNA encapsulated by PACE (IN PACE-Spike) 14 days after IM Prime. Six weeks after boost, mice were challenged with 6 × 10^4^ PFU SCV2 (2019n-CoV/USA_WA1/2020). Weight loss and survival were monitored up to 14 days after infection. (**J** to **L**) Weight loss and survival of naïve, IM prime, or prime and PACE-spike K18-hACE2 mice from 1 to 14 days after infection. Mean ± SEM. Statistical significance was calculated by means of [(D) and (L)] log-rank Mantel-Cox test, [(E) and (F)] one-way ANOVA followed by Tukey’s correction, or (G) Student’s *t* test; *P ≤ 0.05, **P ≤ 0.01, ***P ≤ 0.001, ****P ≤ 0.0001. Individual data points are represented and are pooled from two independent experiments.

Naïve, LD prime only, or LD P&S mice were challenged with SARS-CoV-2 and assessed for viral burden at 2 days after infection, assessed for lungs pathology at 5 days after infection, or monitored for weight loss and mortality for 14 days (Fig. 3A). All mice vaccinated with P&S were completely protected from weight loss or death, but neither naïve nor LD prime–only mice were protected (Fig. 3, B to D). This protection was accompanied by reduced viral burden in both the upper respiratory tract (nasal turbinates) and lower respiratory tract (lungs) (Fig. 3, E and F). Furthermore, P&S led to significant protection from lung pathology, with only one of six mice developing limited mononuclear infiltrates at 5 days after infection (Fig. 3, G and H). Next, to assess the protective capacity of PACE-spike IN boost, we again immunized mice with LD mRNA-LNP intramuscularly and boosted them intranasally with PACE-spike mRNA. Prime and PACE-spike resulted in significant protection from morbidity and mortality (Fig. 3, I to L). Thus, P&S represents a robust, versatile, and safe vaccine strategy because IN boosting by either IN unadjuvanted spike or PACE-spike is sufficient to induce mucosal immunity and to provide protection against lethal challenge and COVID-19–like pulmonary disease.

### P&S achieves robust systemic booster responses similar to parenteral mRNA-LNP

**Fig. 4.**
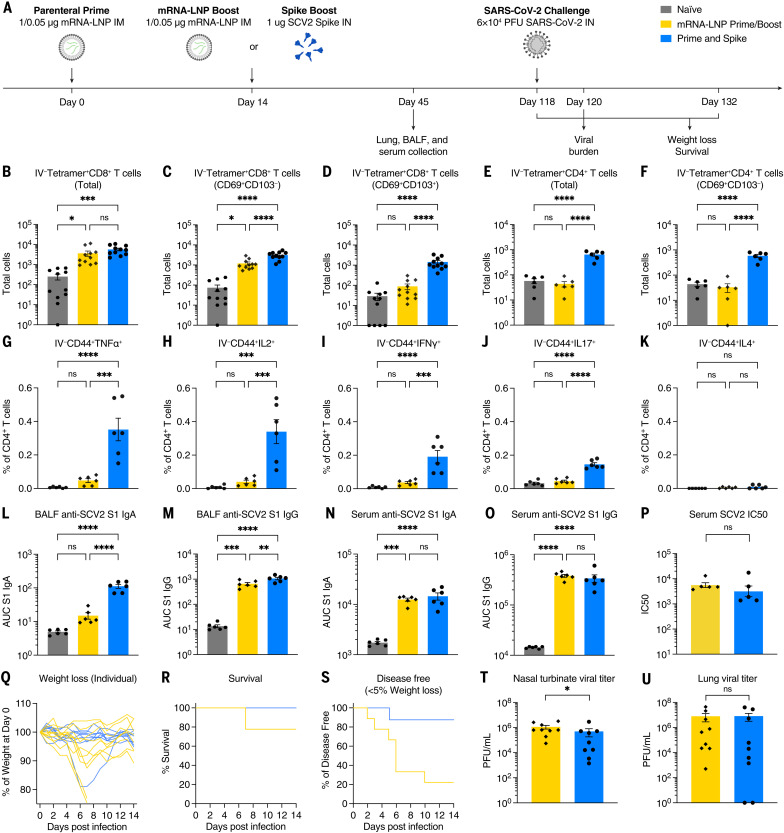
IN spike boosting elicits enhanced mucosal immunity with similar systemic humoral responses to IM mRNA-LNP boosting. (**A**) Experimental schema. K18-hACE2 mice were IM primed with 1 μg of mRNA-LNP, followed 14 days later by boosting with 1 μg of mRNA-LNP IM or 1 μg of SCV2 spike IN. Forty-five days after prime, lung tissues were collected for T cell analysis by means of flow cytometry, and BALF and serum were collected for antibody measurement. K18-hACE2 mice were intramuscularly primed with 0.05 μg of mRNA-LNP, followed 14 days later by boosting with 0.05 μg of mRNA-LNP intramuscularly, or 1 μg of SCV2 spike intranasally and challenged with 6 × 10^4^ PFU SCV2 at 118 days after prime. (**B** to **D**) Quantification of total tetramer^+^ CD8 T cells, CD69^+^CD103^−^tetramer^+^ CD8 T cells, or CD69^+^CD103^+^tetramer^+^ CD8 T cells in lung tissues from naïve, mRNA-LNP prime-boost, or P&S mice. (**E** to **F**) Quantification of total tetramer^+^ CD4 T cells or CD69^+^CD103^−^tetramer^+^ CD4 T cells in lung tissues. (**G** to **K**) Lung lymphocytes were isolated by means of Percoll gradient and restimulated with spike peptide megapool from SCV2. Intracellular cytokine staining was performed to assess antigen-specific production of TNF-α, IL-2, IFN-γ, IL-17, and IL-4 by extravascular IV-CD45^−^CD44^+^ CD4 T cells. (**L** to **O**) Measurement of SCV2 spike S1 subunit–specific (L) BALF IgA, (M) BALF IgG, (N) serum IgA, and (O) serum IgG in naïve, mRNA-LNP prime-boost, or P&S mice. (**P**) Measurement of neutralization titer against SCV2 spike–pseudotyped VSV. (**Q** to **S**) Weight loss, survival, and disease-free survival (<5% maximum weight loss) of mRNA-LNP prime-boost or P&S mice from 1 to 14 days after infection. (**T** and **U**) Measurement of infectious virus titer in lung and nasal turbinate tissues at 2 days after infection by means of plaque assay. To reduce overall number of experimental animals used, control data points from naïve and mRNA prime-boost are common to Figs. 4 and 6. Mean ± SEM. Statistical significance was calculated by means of [(B) to (O)] one-way ANOVA followed by Tukey’s correction or [(P), (T), and (U)] Student’s *t* test, and [(R) and (S)] log-rank Mantel-Cox test; *P ≤ 0.05, **P ≤ 0.01, ***P ≤ 0.001, ****P ≤ 0.0001. Individual data points are represented and are pooled from two independent experiments.

IM mRNA-LNP–based boosts are the current standard. Thus, we compared systemic and mucosal immune responses in P&S-vaccinated and IM mRNA-LNP prime-boost–vaccinated mice (Fig. 4A). Only P&S-vaccinated animals developed lung IV-CD45^−^tetramer^+^CD8^+^ T cells that express CD69^+^ and CD103^+^ (Fig. 4, B to D). The peptide sequence corresponding to spike 62-76 (S62-76) is an epitope recognized by CD4^+^ T cells in convalescent C57BL/6 mice (*33*). We therefore developed an MHC class II tetramer S62-76 (VTWFHAIHVSGTNGT) that readily identified lung-resident CD4^+^ T cells in both P&S and convalescent mice (fig. S5). Both infection and vaccination similarly led to increased IV-CD45^−^tetramer^+^CD4^+^CD69^+^CD103^−^ T_RM_ cells. P&S induced significantly higher levels of lung-resident antigen-specific CD4^+^ T cells that phenotypically resemble infection-induced CD4^+^ T cells (IV-CD45^−^tetramer^+^CD69^+^CD4^+^) (Fig. 4, E and F). To further characterize the CD4^+^ T_RM_ cell response, we used a peptide stimulation assay and found that P&S led to a higher number of tissue-resident CD4^+^ T helper type 1 (T_H_1) and T_H_17 but not T_H_2 CD4^+^ T cells (Fig. 4, G to K). P&S also led to the induction of polyfunctional lung resident T_H_1 cells (fig. S6, B to E).

P&S-vaccinated but not prime-boost–vaccinated animals developed increased levels of BALF IgA (Fig. 4L). Although BALF IgG levels were increased in prime-boost relative to naïve, P&S developed significantly higher BALF IgG than that of prime-boost (Fig. 4M). Serum IgA and IgG in prime-boost– and P&S-vaccinated mice were similar (Fig. 4, N and O), as were neutralizing antibody levels (Fig. 4P). Thus, P&S induces similar systemic binding and neutralizing antibody levels—a correlate of protection in humans—and elicits mucosal IgA, IgG, CD4^+^ T_RM_ cells, and CD8^+^ T_RM_ cells. Only P&S elicits T_H_1 and T_H_17 CD4^+^ T_RM_ cells and not pathogenic T_H_2 cell responses, which have been associated with vaccine-associated enhanced disease (VAED) (*34*).

To compare the protective efficacy of P&S to mRNA-LNP prime-boost, mice were primed with LD mRNA-LNP and boosted with either LD mRNA-LNP (intramuscularly) or unadjuvanted spike protein (intranasally). Mice were challenged 118 days after prime. Both vaccine strategies led to roughly equivalent protection from death, with two of nine prime-boost mice and zero of nine P&S mice succumbing to infection (Fig. 4, Q and R). P&S led to significantly enhanced disease-free survival indicated by only one of nine mice losing >5% of initial body weight, whereas six of nine mRNA-LNP prime-boost mice lost >5% of their starting body weight (Fig. 4S). P&S also led to enhanced upper-airway protection, indicated by decreased nasal turbinate viral load, and reduced although not statistically significant lower airway viral load (Fig. 4, T and U).

### P&S reduces transmission in a hamster model of SARS-CoV-2

**Fig. 5.**
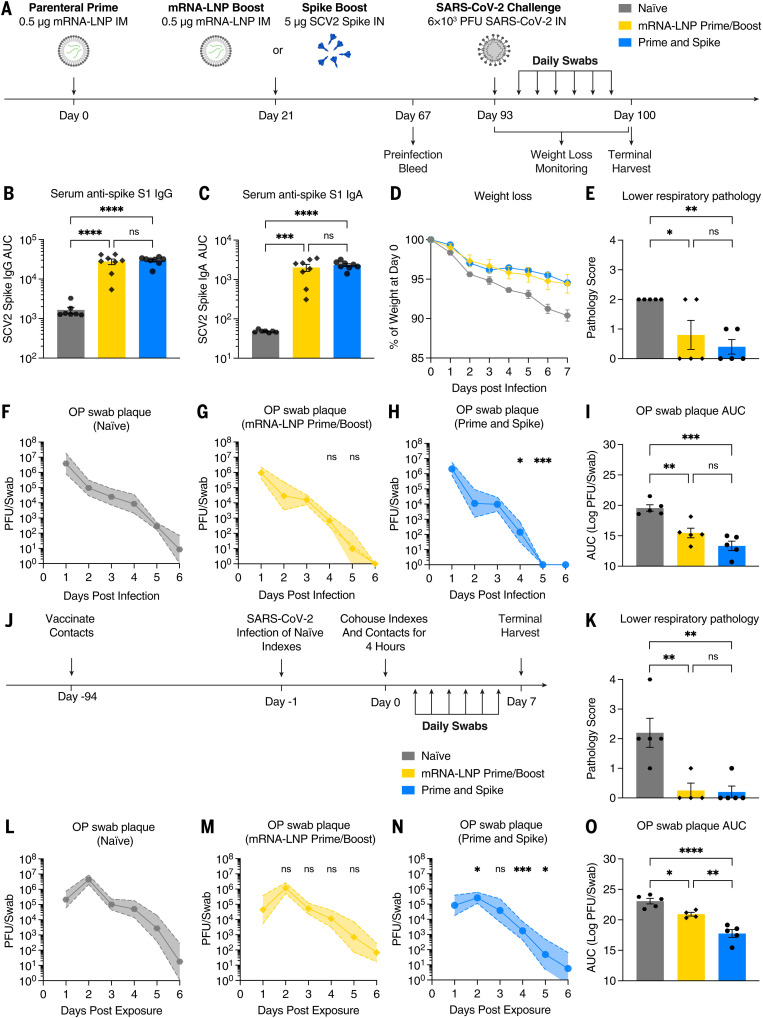
IN spike boosting leads to reduced viral transmission in hamster model. (**A**) Experimental schema. Syrian hamsters were intramuscularly primed with 0.5 μg of mRNA-LNP, followed 21 days later by boosting with 0.5 μg of mRNA-LNP intramuscularly or 5 μg of SCV2 spike intranasally. (**B** and **C**) Sixty-seven days after prime, serum IgG and IgA were assessed by means of ELISA. At 93 days after prime, naïve, mRNA-LNP prime-boost, and P&S hamsters were infected with 6 × 10^3^ PFU SCV2. (**D**) Weight loss as percent of starting. (**E**) Histopathologic analysis of lung samples at 7 days after infection. (**F** to **H**) Viral titer from oropharyngeal swabs are shown as mean (symbols) and standard deviation (shaded regions), *P* value relative to control at the same time point. (**I**) AUC analysis for viral titer over 6 days after infection. (**J**) Transmission experimental schema. Syrian hamsters vaccinated as above were cohoused for 4 hours with naïve donor hamsters that had been infected 24 hours earlier with 6 × 10^3^ PFU SCV2. (**K**) Histopathologic analysis of lung samples at 7 days after exposure. (**L** to **N**) Viral titers from oropharyngeal swabs are shown as mean (symbols) and standard deviation (shade), *P* value relative to control at the same time point. (**O**) AUC analysis for viral titer over 6 days after infection. Mean ± SEM. Statistical significance was calculated by means of [(B), (C), (E), (I), (K), and (O)] one-way ANOVA followed by Tukey’s correction, [(F) to (H)] mixed-effect analysis followed by Tukey’s multiple comparison test, or [(L) to (N)] two-way ANOVA followed by Dunnett’s multiple comparisons test; *P ≤ 0.05, **P ≤ 0.01, ***P ≤ 0.001, ****P ≤ 0.0001. Individual data points are represented from one independent experiment.

Next, we used Syrian hamsters to assess both the viability of P&S in an alternate SARS-CoV-2 model and its ability to reduce transmission. Hamsters were vaccinated by means of either IM mRNA-LNP prime-boost or P&S (Fig. 5A). Serum IgA and IgG levels at 67 days after prime were equivalent between the two groups (Fig. 5, B and C). Hamsters were infected with SARS-CoV-2 at 93 days after prime, and both groups were equivalently protected from disease, as indicated by minimal weight loss and reduced lung pathology relative to those of naïve animals (Fig. 5, D and E). P&S-vaccinated animals cleared viral shedding more quickly relative to naïve controls starting at 4 days after infection, with all oral swabs negative for infectious virus by 5 days after infection. Conversely, mRNA-LNP prime-boost animals did not have significantly lower titers at 4 or 5 days after infection and did not stop shedding virus until 6 days after infection. Cumulative viral shedding assessed with area under the curve (AUC) revealed that both mRNA-LNP prime-boost and P&S-vaccinated animals had significantly lower overall viral shedding than naïve animals. Although P&S AUC was less than mRNA-LNP prime-boost, the results were not statistically significant.

Although P&S reduced viral shedding after infection, whether P&S was able to reduce transmission to vaccinated animals was not yet determined. Vaccinated hamsters were therefore cohoused with naïve donor hamsters that had been infected 24 hours prior (Fig. 5J). P&S-vaccinated contact hamsters had significantly lower viral titers at days 2, 4, and 5 after exposure relative to naïve, whereas mRNA prime-boost–vaccinated animals did not have significantly reduced viral shedding at any single time point after exposure (Fig. 5, L to N). Both P&S and mRNA prime-boost were equally protected from lower respiratory tract pathology in the setting of transmission (Fig. 5K and fig. S7). Peak viral load (at 2 days after infection) and cumulative viral shedding were significantly reduced in P&S-vaccinated animals relative to both naïve and mRNA-LNP prime-boost contact hamsters (Fig. 5O). Thus, P&S appears to be an effective vaccine strategy in hamsters and reduces viral transmission.

### Heterologous spike robustly elicits cross-reactive immunity

Boosting at a distinct anatomic location—in this case, the respiratory mucosa—by homologous unadjuvanted subunit spike enables the formation of new mucosal immune memory and enhances systemic immunity. However, VOCs such as current Omicron sublineages have substantial changes to the spike protein sequence, leading to evasion of preexisting humoral immunity. It is likely that future variants will diverge even more, which suggests that a boosting strategy that elicits broadly reactive immunity will be necessary to neutralize future variants.

**Fig. 6.**
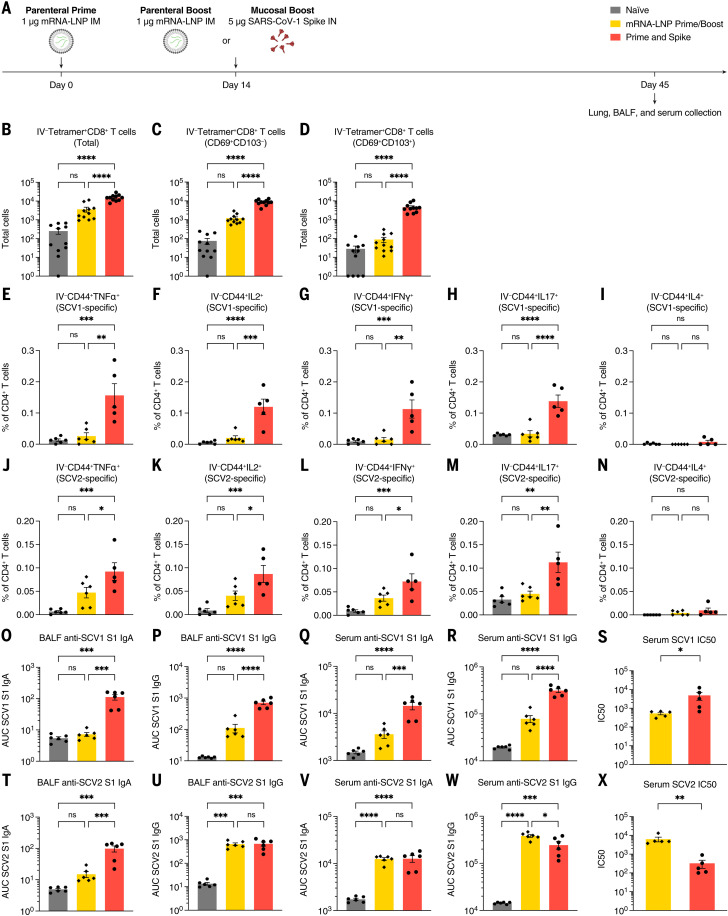
Heterologous IN boosting with SARS-CoV-1 spike enhances preexisting SCV2-specific immunity and broadens reactivities to SCV1. (**A**) Experimental schema. K18-hACE2 mice were intramuscularly primed with 1 μg of mRNA-LNP, followed by boosting with 1 μg of mRNA-LNP intramuscularly, or 5 μg of prefusion-stabilized, trimeric, recombinant SARS-CoV-1 (SCV1) spike IN (IN SpikeX) 14 days after prime. (**B** to **D**) Quantification of total tetramer^+^ CD8 T cells, CD69^+^CD103^−^tetramer^+^ CD8 T cells, or CD69^+^CD103^+^tetramer^+^ CD8 T cells in lung tissues from naïve, mRNA-LNP prime-boost, or P&Sx mice. (**E** to **N**) Percoll gradient purified lung lymphocytes were restimulated with spike peptide megapool from [(E) to (I)] SCV1 or [(J) to (N)] SCV2, and intracellular cytokine staining was performed to assess antigen-specific production of TNF-α, IL-2, IFN-γ, IL-17, and IL-4 by extravascular IV-CD45^−^CD44^+^ CD4 T cells. (**O** to **S**) Measurement of SCV1 spike S1 subunit–specific BALF IgA and IgG, and serum IgA and IgG. (S) Measurement of neutralization titer against SCV1 spike–pseudotyped VSV. (**T** to **W**) Measurement of SCV2 spike S1 subunit–specific BALF IgA and IgG, and serum IgA and IgG. (**X**) Measurement of neutralization titer against SCV2 spike–pseudotyped VSV. To reduce overall number of experimental animals used, control data points from naïve and mRNA prime-boost are common to Figs. 4 and 6. Mean ± SEM. Statistical significance was calculated by means of one-way ANOVA followed by Tukey’s correction, except for [(S) and (X)] Student’s *t* test; *P ≤ 0.05, **P ≤ 0.01, ***P ≤ 0.001, ****P ≤ 0.0001. Individual data points are represented and are pooled from two independent experiments.

To test the ability of an unadjuvanted heterologous spike (Spike X) protein in P&S, mice were primed with SARS-CoV-2 mRNA-LNP followed by IN boosting with SARS-CoV-1 spike, which we refer to as P&Sx (Fig. 6A). Although SARS-CoV-1 is a related sarbecovirus, its spike protein only shares 76% homology with SARS-CoV-2 spike. At 45 days after prime, there were increased IV-CD45^−^ tetramer^+^ CD8^+^ T_RM_ cells (Fig. 6, B to D). The MHC class I tetramer sequence was highly conserved within the sarbecovirus family (fig. S5A). We performed peptide stimulation assay using both SARS-CoV-1 and SARS-CoV-2 peptide pools to assess the development of antigen-specific lung CD4^+^ T_RM_ cells. We found that P&Sx led to both the development of SARS-CoV-1–specific and to a lesser extent SARS-CoV-2–specific antigen-specific T_H_1 and T_H_17 CD4^+^ T_RM_ cells and no induction of CD4^+^ T_RM_ expressing the T_H_2 cytokine interleukin-4 (IL-4) (Fig. 6, E to N, and fig. S8). There were also increased anti–SARS-CoV-1 IgA and IgG in both the BALF and serum in P&Sx relative to IM mRNA-LNP prime-boost (Fig. 6, O to R). P&Sx mice correspondingly developed higher neutralization titers against SARS-CoV-1 than those of mice vaccinated with SARS-CoV-2 mRNA-LNP prime-boost (Fig. 6S). P&Sx induced higher anti–SARS-CoV-2 BALF IgA than SARS-CoV-2 mRNA-LNP prime-boost and similar levels of anti–SARS-CoV-2 IgG in BALF (Fig. 6, T and U). Consistent with the elevated serum IgG levels, mRNA-LNP prime-boost mice had higher serum neutralization titers against SARS-CoV-2 than that of P&Sx mice (Fig. 6, V to X). Thus, IN boosting with unadjuvanted heterologous spike protein can induce potent mucosal cellular and humoral memory against a substantially divergent sarbecovirus.

## Discussion

In this work, we describe the preclinical development of an alternative vaccine strategy, P&S, in which IN unadjuvanted spike subunit protein elicits robust protective mucosal immunity after mRNA-LNP parenteral immunization. These enhanced mucosal responses are characterized by the expansion of antigen-specific CD8^+^ T_RM_, CD4^+^ T_RM_, and B_RM_ cells as well as mucosal secretion of IgA and IgG. We found that an IN unadjuvanted spike booster can be administered months out from primary immunization and that it offers systemic neutralizing antibody booster responses comparable with that of IM mRNA-LNP boost. Similarly, prime and PACE-spike elicits increased antigen-specific CD8^+^ T_RM_ cells and mucosal IgA. Both boosting methods result in protection from lethal SARS-CoV-2 challenge. We also found that P&S leads to durable responses with protective vaccine efficacy at 118 days from the initiation of vaccination. P&S is protective in hamsters and blocks viral transmission more effectively than does mRNA-LNP prime-boost. Last, by using a divergent spike antigen, we demonstrate that P&Sx can generate mucosal immunity to SARS-CoV-1 while also boosting the systemic and mucosal neutralizing antibodies to the original antigenic target, SARS-CoV-2. Although the goal of vaccination has been to prevent individual morbidity and mortality, the evolution of SARS-CoV-2 has highlighted the need for rapidly deployable mucosal vaccines that also prevent transmission. P&S shows promise in reducing both infection and transmission. Improving upon current vaccine platforms to provide mucosal immunity is vital to control this pandemic and will certainly be important for the next.

Preclinical studies of both SARS-CoV-2 and influenza have demonstrated that IN vaccination decreases viral shedding and transmission relative to those of parenteral vaccines (*19*–*23*). Despite these studies, there is only one currently approved respiratory mucosal vaccine, FluMist, which relies on a live attenuated influenza virus. FluMist is contraindicated in people with underlying respiratory conditions and is only approved for young people. Additionally, live attenuated vaccines are not amenable to rapid implementation because this technology requires extensive research and development. Accordingly, most current clinical trials of mucosally administered SARS-CoV-2 vaccines rely on either replication-deficient or attenuated viral vectors. However, the safety and efficacy of these approaches have not yet been established, especially given that preexisting immunity to these vectors can lead to reduced immunogenicity (*35*). Some vector-based mucosal vaccines—including two Merck candidates, V590 and V591—have already been abandoned after phase 1 clinical trials showed poor immunogenicity, whereas candidates by Bharat Biotech and CanSino have recently been approved (*36*).

P&S is likely broadly applicable as a booster against new SARS-CoV-2 VOCs in a previously vaccinated individual or as a de novo primary immunization strategy for newly emerging respiratory pathogens. Although it is possible that our results rely on specific characteristics of mRNA-LNP priming, we believe that this approach will likely work with other primary immunization regimens or in the case of previous infection. Although the above study assesses a single mRNA-LNP dose before IN boosting, we would expect unadjuvanted IN boosting to be as effective if not more so in individuals who have received multiple previous shots because P&S seems to leverage preexisting immunity rather than be inhibited by it. Additionally, it has been shown that the highly stabilized spike enhances its immunogenicity and that applying this vaccination strategy to other pathogens may require the addition of stabilizing mutations to enable unadjuvanted boosting. Our present study characterizes a method for the development of mucosal immunity to SARS-CoV-2 without the use of adjuvants or replicating viruses or vectors in two different well-validated preclinical vaccine models. These results are encouraging but require further validation and optimization for human use.

Vaccines that generate broadly neutralizing immunity against a wide variety of sarbecoviruses are a goal to combat both newly emerging SARS-CoV-2 variants and potential pandemic SARS-like coronaviruses. Using SARS-CoV-1 spike as a heterologous IN boost, P&Sx demonstrates that prior SARS-CoV-2 mRNA-LNP does not prevent the development of SARS-CoV-1–neutralizing antibodies but rather enables it. P&Sx simultaneously elicits broadly reactive neutralizing antibodies and mucosal immunity. Although some recent studies have successfully reported the development of systemic pan-sarbecovirus vaccines (*37*, *38*), P&Sx induces both systemic and mucosal immunity against both SARS-CoV-1 and SARS-CoV-2.

SARS-CoV-2 will continue to evolve and become more immune-evasive and transmissible. We will require boosting in human populations for the foreseeable future. Boosting that induces mucosal immunity may help enhance protection and slow transmission as these new variants emerge.

## Materials and methods

All procedures were performed in a BSL-3 facility (for SARS-CoV-2–infected mice) with approval from the Yale Institutional Animal Care and Use Committee and Yale Environmental Health and Safety.

### Cell and virus

As reported previously (*15*, *39*, *40*), Vero E6 cells overexpressing angiotensin-converting enzyme 2 (ACE2) and TMPRSS2 [kindly provided by B. Graham at the National Institutes of Health Vaccine Research Center (NIH-VRC)] were cultured in Dulbecco’s modified Eagle medium (DMEM) supplemented with 1% sodium pyruvate and 5% fetal bovine serum (FBS) at 37°C and 5% CO_2_. SARS-CoV-2 isolate hCOV-19/USA-WA1/2020 (NR-52281) was obtained from BEI Resources and was amplified in VeroE6 cells overexpressing ACE2 and TMPRSS2. Cells were infected at a multiplicity of infection 0.01 for 2 to 3 days to generate a working stock, and after incubation, the supernatant was clarified by means of centrifugation (5 min, 500*g*) and filtered through a 0.45-μm filter and stored at −80°C. Viral titers were measured with standard plaque assay by using Vero E6 cells overexpressing hACE2 and TMPRSS2.

### Animals

B6.Cg-Tg(K18-ACE2)2Prlmn/J (K18-hACE2) mice (stock no. 034860) were purchased from the Jackson Laboratory and subsequently bred and housed at Yale University. Eight- to twelve-week-old female mice were used for immunization experiments. Male Syrian hamsters (strain, HSdHan:AURA; stock no. 089) were purchased from Envigo, and vaccination began at 12 weeks of age. All procedures used in this study (such as sex matching and age matching) complied with federal guidelines and the institutional policies of the Yale School of Medicine Animal Care and Use Committee. To reduce the overall number of experimental animals used and to be consistent with our institutional animal use policy, control data points are shared among some figures when applicable and noted in figure legends. Sample sizes for animal experiments were determined empirically on the basis of previously published work in the field with similar experimental paradigms to provide sufficient statistical power for assessing biological effects of interest. No statistical methods were used to predetermine the sample size. Age- and sex-matched animals were randomly assigned to experimental groups at the beginning of the experiment. Investigators were not blinded, except for pathological analysis, because no subjective measurements were performed.

### SARS-CoV-2 infection

Mice were anesthetized by using 30% v/v isoflurane diluted in propylene glycol. Using a pipette, 50 μl containing 6 × 10^4^ plaque-forming units (PFU) SARS-CoV-2 was delivered intranasally. Hamsters were anesthetized by using 30% v/v isoflurane diluted in propylene glycol and administered 6 × 10^3^ PFU SARS-CoV-2 intranasally in 100 μl.

### mRNA extraction from Comirnaty (BNT162b2) mRNA-LNP

mRNA was extracted from the vaccine formulation with a TRIzol-chloroform separation method as previously described (*41*). Briefly, aliquots of vaccine were dissolved in TRIzol LS (Thermo Fisher Scientific) at 1:6.6 vaccine to TRIzol volume ratio. After a 15-min incubation (37°C, shaking), 0.2 ml of chloroform was added per 1 ml of TRIzol. The solution was shaken vigorously for 1 min and then incubated at room temperature for 3 min. The solution was centrifuged at 12,000*g* for 8 min at 4°C. The aqueous layer containing the isolated mRNA was further purified with a RNeasy Maxi Kit purchased from Qiagen (Germantown, MD, USA) following the manufacturers protocol. The RNA was eluted from the column on the final step with sodium acetate buffer (25 mM, pH 5.8) warmed to 37°C. Extracted mRNA was analyzed for concentration and purity with NanoDrop measurements of the absorbance at 260, 280, and 230 nm, with purity being assessed as A260/A280 > 2 and A260/A230 > 2. Agarose gel electrophoresis was used to determine the length and verify that the mRNA remained intact. Extracted mRNA containing 1:100 SYBR Safe stain (Thermo Fisher Scientific) was loaded onto a 1% agarose gel and run at 75 V with tris-acetate-EDTA buffer containing 1:5000 SYBR Safe stain (fig. S9).

### PACE polyplex formulation and characterization

PACE polymers were synthesized and characterized as previously described (*42*). All polyplexes were formulated at a 50:1 weight ratio of polymer to mRNA. PACE polymers were dissolved at 100 mg/ml overnight in dimethyl sulfoxide (37°C, shaking). Before polyplex fabrication, an optimal PACE polymer blend was produced by mixing solutions of PACE polymers containing an end-group modification (*43*) and a polyethylene glycol tail (*30*). mRNA and polymer were diluted into equal volumes of sodium acetate buffer (25 mM, pH 5.8). The polymer dilution was then vortexed for 15 s, mixed with the mRNA dilution, and vortexed for an additional 25 s. Polyplexes were incubated at room temperature for 10 min before use.

### Vaccination

Used vials of Comirnaty vaccine were acquired from Yale Health pharmacy within 24 hours of opening and stored at 4°C. Vials contained residual vaccine (diluted to 100 μg/ml per manufacturer’s instructions), which was removed with spinal syringe and pooled. Pooled residual vaccine was aliquoted and stored at −80°C. Mice were anesthetized by using a mixture of ketamine (50 mg per kilogram of body weight) and xylazine (5 mg per kilogram of body weight) and injected intraperitoneally. Vaccine was diluted in sterile phosphate-buffered saline (PBS) and 10 or 20 μl was injected into the left quadriceps muscle with a 31G syringe for a final dose of 1 or 0.05 μg as indicated. Similarly, hamsters were administered 0.5 μg diluted in 20 μl by means of 31G syringe in the left quadriceps muscle. For IN vaccination, SARS-CoV-2–stabilized spike (ACRO biosystems, SPN-C52H9) or SARS-CoV-1 spike (ACRO biosystems, SPN-S52H6) was reconstituted in sterile endotoxin-free water according to the manufacturer’s protocol and then diluted in sterile PBS and stored at −80°C. Mice or hamsters were anesthetized by using isoflurane and administered 1 or 5 μg (as indicated) in 50 μl (25 μl where indicated) through the IN route. For IN mRNA-PACE, 50 μl of polyplexes in solution was administered at the indicated dose.

### Viral titer analysis

Viral titer analysis was performed as previously described (*15*, *39*, *40*), with modifications noted and summarized here. Mice were euthanized in 100% isoflurane at indicated time points. Approximately half of the total lung (right lobes) or nasal turbinate was homogenized in a bead homogenizer tube containing 1 ml of PBS supplemented with 2% FBS and 2% antibiotics/antimycotics (Gibco) and stored at −80°C. Nasal turbinate and lung homogenates were clarified of debris by centrifugation (10 min, 3100*g*). Daily oral swabs (Pruitan PurFlock Ultra 25-3206-U) were performed on hamsters and stored in 1 ml of DMEM with 2% FBS and 2% antibiotics/antimycotics (Gibco) and stored at −80°C. To determine infectious SARS-CoV-2 titers, plaque assay was performed by using ACE2- and TMPRSS2-overexpressing Vero E6 cells. Plaques were resolved by means of formalin fixation 40 to 42 hours after infection, followed by staining with crystal violet and rinsing with water for plaque visualization.

### SARS-CoV-2–specific antibody measurements

Enzyme-linked immunosorbent assays (ELISAs) were performed as previously described (*39*, *44*), with modifications noted and summarized here. Ninety-six–well MaxiSorp plates (Thermo Scientific 442404) were coated with recombinant SARS-CoV-2 S1 protein (ACRO Biosystems S1N-C52H3) or SARS-CoV-1 S1 protein (ACRO Biosystems S1N-S52H5). After overnight incubation at 4°C, plates were replaced with blocking solution (PBS with 0.1% Tween-20, and 5% milk powder) and incubated for 1 to 2 hours at room temperature. Serum or BALF was diluted in dilution solution (PBS with 0.1% Tween-20 and 2% milk powder) and added to plates for 2 hours at room temperature. Plates were washed five times with PBS-T (PBS with 0.05% Tween-20) by using an automatic plate washer (250 μl per cycle), and 50 μl of horseradish peroxidase (HRP) anti-mouse IgG (Cell Signaling Technology 7076; 1:3000), HRP anti-mouse IgA (Southern Biotech 1040-05; 1:1000), HRP anti-hamster IgG (Southern Biotech 6060-05; 1:1000), or rabbit anti-hamster IgA HRP (Brookwood Biomedical, sab3003a, 1:250 100 μg/ml) diluted in dilution solution was added to each well. After 1 hour of incubation at room temperature (overnight at 4°C for hamster IgA), plates were washed three times with PBS-T by using an automatic plate washer. Fifty μl of TMB Substrate Reagent Set (BD Biosciences 555214) was added to plates. To terminate the reaction, another 50 μl of 2 N sulfuric acid was added after 15 min of substrate development. Plates were then recorded at wavelengths of 450 and 570 nm, and the difference was reported as AUC.

### Immunohistochemistry and pathological analysis

Yale Pathology Tissue Services (YPTS) performed embedding, sectioning, and hematoxylin and eosin (H&E) staining of lung tissue. A pulmonary pathologist reviewed the slides blinded and identified immune cell infiltration and other related pathologies. Mouse lung scores of 1 to 4 were characterized as follows: 1, mild patchy mononuclear infiltrate, parenchymal and perivascular, with variably reactive pneumocytes and stromal rection; 2, moderate patchy mononuclear infiltrate, parenchymal and perivascular, with variably reactive pneumocytes and stromal rection; 3, mild, dense mixed infiltrate, including mononuclear cells and granulocytes and neutrophils; and 4, moderate, dense mixed infiltrate, including mononuclear cells and granulocytes and neutrophils. Hamster lung scores of 0 to 4 were characterized as follows: 0, normal; 1, very focal injury, inflammation, and repair; 2, multifocal repair; and 4, multifocal repair with necrosis.

### Intravascular labeling, cell isolation, and flow cytometry

To discriminate circulating from extravascular immune cells, mice were anesthetized with 30% isoflurane and injected intravenously with 2 μg of APC/Fire 750–labeled anti-CD45 Ab. After 3 min of labeling, mice were euthanized. Tissues were harvested and analyzed as previously described (*39*). Briefly, lungs and nasal turbinates were minced with scissors, incubated in a digestion cocktail containing collagenase A (Roche) and DNase I (Sigma-Aldrich) in RPMI at 37°C for 45 min, and dissociated through a 70-μm filter. Airway-resident immune cells were collected by centrifuging BALF at 600*g* for 5 min at 4°C, after which cell pellets were used for flow cytometry, and supernatants were used for antibody analysis. Cells were treated with ammonium-chloride-potassium (ACK) buffer to lyse red blood cells and then washed once with PBS. Single-cell suspensions were incubated with Fixable Aqua cell viability dye (Invitrogen L34957) and anti-mouse CD16/CD32 Fc Block (BD Biosciences 553141) for 30 min at 4°C. Cells were washed once with PBS before surface staining. For T cell analysis, cells were first stained with APC-labeled SARS-CoV-2 S 62-76 MHC class II tetramer [I-A(b)] for 60 min at RT. Cells were washed once with PBS and then stained with anti-CD103, anti-CD3, anti-CD44, anti-CD62L, anti-CD8a, anti-CD69, anti-CD183 (CXCR3), anti-CD4, and PE-SARS-CoV-2 S 539-546 MHC class I tetramer [H-2K(b)] for 30 min at 4°C. For B cell analysis, cells were stained with ant-GL7, anti-IgM, anti-CD138, anti-CD19, anti-IgA, anti-B220, PE-SARS-CoV-2 RBD tetramer, anti-CD38, APC-SARS-CoV-2 RBD tetramer, and anti-IgD for 30 min at 4°C. Cells were washed with PBS once, followed by 4% paraformaldehyde fixation for 45 min at 4°C. Flow cytometry data were acquired on an Attune NxT Flow Cytometer and analyzed by use of FlowJo Software (10.5.3; Tree Star). Gating strategy is provided in fig. S10, and detailed antibody information is provided in table S1.

### Intracellular cytokine staining assay for detection of lung-resident spike-specific CD4 T cells

After intravascular labeling by using an anti-CD45 Ab at the dose of 2 μg per mouse, lung isolation, and processing, single cells from the lung tissue were first enriched by using a Percoll gradient before spike peptide stimulation. Briefly, total lung cells were first resuspended in 5 ml of 30% Percoll solution in a 15-ml conical tube, underlaid with 5 ml of 70% Percoll solution, and subject to centrifugation at 1000*g* for 20 min at room temperature. After centrifugation, lymphocytes located at the interphase between 30 and 70% Percoll solution were collected, washed once with PBS, and resuspended in complete RPMI. In a 96-well U-bottom plate, 10^6^ lymphocytes enriched from each lung sample were added, together with spike peptide megapool from SARS-CoV-2 (JPT PM-WCPV-S-1) or SARS-CoV-1 (JPT PM-CVHSA-S-1) at a final working concentration of 1 μg/ml per peptide, 1X Protein Transport Inhibitor Cocktail (eBioscience 00-4980-03), and 10^6^ freshly isolated splenocytes from CD45.1^+^ mice, with complete RPMI for a final volume of 200 μl. Peptide stimulation was performed for 8 hours at 37°C. After peptide stimulation, cells were incubated at 4°C with Fc block (BioXCell BE0307) and Aqua cell viability dye (ThermoFisher L34957) for 20 min. Cells were washed once with PBS before surface staining with anti-CD3, anti-CD44, anti-CD4, and anti-CD45.1. After washing with PBS, cells were fixed by using 4% paraformaldehyde for 45 min at 4°C. Cells were then washed and permeabilized with 1X Permeabilization Buffer (eBioscience 00-8333-56) for 10 min at RT. After permeabilization, cells were stained with anti–IL-4, anti–IL-2, anti–tumor necrosis factor–α (TNF-α), anti–IL-17A, and anti–interferon-γ (IFN-γ). Cells were washed once with PBS before being acquired on Attune and analyzed by use of FlowJo. Gating strategy is provided in fig. S10, and detailed antibody information is provided in table S1.

### SARS-CoV-2 RBD B cell tetramer production and staining

Recombinant SARS-CoV-2 spike RBD His Biotin Protein, CF (R&D BT10500-050) was incubated at a 4:1 molar ratio with either streptavidin-PE (Prozyme PJRS25) or streptavidin-APC (Prozyme PJ27S) for 30 min at 4°C. Mixture was then purified and concentrated in an Amicon Ultra (50 kDA MWCO) spin column and washed 1X with sterile cold PBS. The concentration was determined on a NanoDrop 8000 Spectrophotometer (ThermoFisher ND-8000-GL) by using fluorophore-specific absorbances. Tetramers were then diluted to 1.0 μM in PBS and stored at 4°C. For every 2 × 10^7^ to 5 × 10^7^ cells, 1 μl of stock 1.0 μM tetramer was used for staining.

### Pseudovirus production and neutralization assay

Pseudoviruses were produced as previously described (*15*). Spike-encoding plasmid was kindly provided by V. Munster and previously described (*45*). To perform pseudovirus neutralization assays, Vero E6 overexpressing hACE2 and TMPRSS2 (Fig. 1) or Huh7.5 cell (Fig. 5 and fig. S3) were plated (3 × 10^4^) in each well of a white 96-well plate the day before infection. On the day of infection, serum and BALF were heat-inactivated for 30 min at 56°C. Sera shown in Fig. 1 were tested at a starting dilution of 1:50, and BALF samples were tested at a starting dilution of 1:4, both with eight twofold serial dilutions. Sera shown in Fig. 5 and fig. S3 were tested at a starting dilution of 1:40 with eight threefold serial dilutions. Serial dilutions were mixed 1:1 with indicated pseudovirus and incubated for 1 hour at 37°C and 5% CO_2_. Growth medium was then aspirated from the cells and replaced with 100 μl of serum-virus mixture. Twenty-four hours after infection, the infection-antibody mixture was removed, and plates were flash-frozen at −80°C. Thirty μl of passive lysis buffer (Promega) was added to each well, and plates were incubated for 15 min at room temperature. Thirty μl of Renilla-Glo Luciferase Assay System substrate (Promega) was then added to each well and incubated at room temperature for an additional 15 min. Luminescence was measured on a microplate reader (SpectraMax i3, Molecular Devices). Median inhibitory concentration was calculated with Prism 9 (GraphPad Software) nonlinear regression.

### Sequence alignment

The following amino acid sequences of coronavirus Spike proteins used in alignment were obtained from Uniprot/Genebank: Wuhan (P0DTC2), B.1.1.7 (QWE88920.1), B.1.351 (QRN78347.1), B.1.617 (QUD52764.1), B.1.1.28.1 (QRX39425.1), BA.1 (UFO69279.1), BA.2 (UFO69279.1), BA.2.12.1 (UMZ92892.1), BA.4 (UPP14409.1), BA.5 (UOZ45804.1), Khosta (MZ190137.1), Khosta-2 (MZ190138.1), SARS-CoV (AY278489.2), WIV1 (KF367457), and BANAL236 (MZ937003.1). Sequence alignment was performed with MAFFT in JalView (v2.11.2.3).

### Graphical illustrations

Graphical illustrations were made with Biorender.com.

## Supplementary Material

20221027-1

## Supplementary Materials

### Other Supplementary Material for this manuscript includes the following:

MDAR Reproducibility Checklist

## Notes

View/request a protocol for this paper from *Bio-protocol*.
