# Supplementary material for: Unadjuvanted intranasal spike vaccine elicits protective mucosal immunity against sarbecoviruses
Source: Science. 2022 Nov 25;378(6622):eabo2523. doi: 10.1126/science.abo2523 (PMC9798903; doi:10.1126/science.abo2523)
Supplement: 20221027-1 [file science.abo2523.v1.pdf]

Cite as: T. Mao *et al.*, *Science*
10.1126/science.abo2523 (2022).

Unadjuvanted intranasal spike vaccine elicits protective mucosal immunity against sarbecoviruses

Tianyang Mao[1]†, Benjamin Israelow[1,2]*†, Mario A. Peña-Hernández[1], Alexandra Suberi[3], Liqun Zhou[1], Sophia Luyten[1], Melanie Reschke[4], Huiping Dong[1], Robert J. Homer[5], W. Mark Saltzman[3,6,7,8,9], Akiko Iwasaki[1,10]*

[1]Department of Immunobiology, Yale University School of Medicine, New Haven, CT, USA. [2]Department of Medicine, Section of Infectious Diseases, Yale University School of Medicine, New Haven, CT, USA. [3]Department of Biomedical Engineering, Yale University, New Haven, CT, USA. [4]Department of Molecular Biophysics and Biochemistry, Yale University, New Haven, CT, USA. [5]Department of Pathology, Yale University School of Medicine, CT, USA. [6]Department of Chemical and Environmental Engineering, Yale University, New Haven, CT, USA. [7]Department of Cellular and Molecular Physiology, Yale University, New Haven, CT, USA. [8]Department of Cellular and Molecular Physiology, Yale University, New Haven, CT, USA. [9]Department of Dermatology, Yale University, New Haven, CT, USA. [10]Howard Hughes Medical Institute, Chevy Chase, MD, USA.

†These authors contributed equally to ths work.

***Corresponding author. Email: benjamin.goldman-israelow@yale.edu (B.I.); akiko.iwasaki@yale.edu (A.I.)**

The SARS-CoV-2 pandemic has highlighted the need for vaccines that not only prevent disease, but also prevent transmission. Parenteral vaccines induce robust systemic immunity, but poor immunity at the respiratory mucosa. Here we describe the development of a vaccine strategy we term “prime and spike” that leverages existing immunity generated by primary vaccination (prime) to elicit mucosal immune memory within the respiratory tract using unadjuvanted intranasal spike boosters (spike). We show that prime and spike induces robust resident memory B and T cell responses, IgA at the respiratory mucosa, boosts systemic immunity, and completely protects mice with partial immunity from lethal SARS-CoV-2 infection. Using divergent spike proteins, prime and spike enables induction of cross-reactive immunity against sarbecoviruses.

During the past 2 years of the SARS-CoV-2 pandemic, there has been an unprecedented development of highly effective vaccines, utilizing technologies including modified mRNA encapsulated in lipid nanoparticles (LNP) and replication-deficient adenoviral vectors. Phase 3 clinical trials and subsequent post-marketing vaccine effectiveness studies initially showed >90% vaccine efficacy against symptomatic disease (*1*–*3*). Additionally, early transmission studies showed decreased rates of transmission in household members of vaccinated individuals (*4*, *5*). Unfortunately, recent studies have demonstrated decreasing vaccine effectiveness, starting 4 months following a second dose with mRNA-LNP–based regimens and earlier with other vaccines (*6*, *7*). Furthermore, continued viral evolution with increasing immune evasive variants of concern (VOC), most recently Omicron (B.1.529) and its sublineages, has also contributed to decreased vaccine effectiveness (*8*–*10*). With enhanced immune evasion and waning systemic immunity, current vaccines have become less effective at preventing viral transmission, which is likely worsened by increased viral transmissibility, and their poor induction of mucosal immunity (*11*).

Currently approved SARS-CoV-2 vaccines rely on intramuscular (IM) administration, which induces high levels of circulating antibodies, memory B cells, and circulating effector $CD4^+$ and $CD8^+$ T cells in animal models and humans (*12*–*14*). However, parenteral vaccines do not induce high levels of potent antiviral immune memory at sites of infection, such as tissue-resident memory B (B_{RM}) cells and T (T_{RM}) cells as well as mucosal IgG and dimeric IgA (*15*–*17*). This contrasts with infection in humans and mice, where $CD8^+$ T_{RM} and mucosal IgA are robustly induced (*15*, *18*). Vaccines targeting the respiratory mucosa could address the shortcomings of parenteral vaccination, as recent assessments of intranasally delivered SARS-CoV-2 spike encoding adenoviral vectors have shown mucosal immunogenicity as well as protection and reduced viral shedding in mice, hamsters, and nonhuman primates (*19*–*23*).

Although primary respiratory administration of vaccines induces mucosal immunity, systemic priming followed by intranasal (IN) boosting results in similar systemic immunity to systemic prime–boost regimens, but with enhanced mucosal immunity (*24*–*26*). Most examples of recombinant subunit vaccines administered either systemically or IN are co-formulated with adjuvants to enhance immunogenicity. However, administration of vaccines to the respiratory tract in humans has proven difficult. There have been cases of IN adjuvanted inactivated vaccine for seasonal influenza leading to Bell’s palsy, possibly caused by the specific toxin adjuvant mediating inflammation of neurons (*27*).

In the setting of non-protective immunity from parenteral vaccination regimens, we assessed the immunogenicity and protection afforded by IN boosting with SARS-CoV-2 spike. Here, we describe a vaccination strategy that utilizes systemic priming with mRNA-LNP followed by IN boosting with either unadjuvanted spike proteins or an immunosilent polyplex encapsulating spike mRNA.

Results

Intranasal boosting with unadjuvanted SARS-CoV-2 spike induces mucosal humoral immunity.

To assess the potential of IN unadjuvanted subunit vaccine boosting for the development of respiratory mucosal immunity, we decided to harness the strong systemic immunogenicity of mRNA-LNP. We additionally benefited from extensive SARS-CoV-2 spike engineering by using HexaPro, which has been shown to significantly enhance immunogenicity and increase protein stability (*28*).

We vaccinated K18-hACE2 (mice) with mRNA-LNP (Pfizer/BioNTech BNT162b2) by IM injection (prime), followed 14 days later by IN administration of recombinant unadjuvanted spike protein (prime and spike (P&S)). Mice were euthanized at days 21 or 28 and assessed for mucosal humoral immunity (Fig. 1A).

First, we assessed anti-SARS-CoV-2 spike IgG and IgA in nasal wash (Fig. 1, B and C), bronchoalveolar lavage fluid (BALF) (Fig. 1, D and E), and serum (Fig. 1, F and G). Only mice that received P&S developed high levels of anti-SARS-CoV-2 IgA and IgG in the nasal wash and BALF. Neither IM prime nor IN spike alone was sufficient to develop mucosal antibodies. In the serum, prime alone was sufficient to induce low levels of IgA and IgG. By contrast, P&S led to significant systemic boosting of both anti-spike IgA and IgG. These increases in antibody levels correlated with increases in neutralization titers both in BALF (Fig. 1H) and serum (Fig. 1I). Thus, a single-dose unadjuvanted intranasal spike alone is not immunogenic and the induction of a potent mucosal and systemic antibody response by unadjuvanted spike requires prior systemic priming, in this case by mRNA-LNP.

B_{RM} cells in the lungs assist in rapid recall response of antibody-secreting plasma cells upon secondary heterologous challenge in influenza models and may be an important local immune effector in protecting against SARS-CoV-2 (*29*). Using intravenous (IV) CD45 labeling to differentiate circulating immune cells within lung tissue combined with B cell tetramers specific for the receptor binding domain (RBD) of the spike protein, we found that P&S leads to increased antigen-specific B cells within lung tissue (IV-$CD45^-B220^+CD19^+tetramer^+$) (Fig. 1J). We also examined the polyclonal tissue response, which likely represents a more complete set of spike-specific B cells within the lungs. We found increases in class-switched antibody-secreting cells (ASC) (IV-$CD45^-CD19^{+/-}CD138^+$) in lung tissue expressing IgA or IgG (Fig. 1, K and L), and we found increased class-switched B_{RM} cells (IV-$CD45^-B220^+CD19^+IgD^-IgM^-CD38^+$) expressing IgA or IgG (Fig. 1, M and N). Thus, P&S elicits local B cell responses in the lung.

Prime and Spike induces mucosal T cell immunity.

Given that P&S induced respiratory mucosal humoral memory, we next assessed the induction of lung T_{RM} cells. Although adjuvant-free subunit vaccines have not traditionally been potent inducers of antigen-specific T cell responses, we hypothesized that the immune memory generated by mRNA-LNP priming would enable subunit-mediated T cell-boosting responses. To identify spike-specific $CD8^+$ T cells, we utilized major histocompatibility complex (MHC) class I tetramer $S_{539-546}$ (VNFNFNGL). There was a significant induction of IV-$CD45^-tetramer^+$ $CD8^+$ T cells, which expressed canonical markers of T_{RM} cells including $CD69^+$ and $CD103^+$, within lung tissue (Fig. 2, A to C), BALF (Fig. 2, D to F), and the nasal turbinate (Fig. 2, G to I). Moreover, there were significant increases in antigen-experienced $CD4^+$ T cells (IV-$CD45^-CD44^+CD4^+$), many of which also expressed $CD69^+$ and $CD103^+$ both within lung tissue (Fig. 2, J to L) and in the BALF (Fig. 2, M to O). Thus, P&S recruits expansion of lung parenchyma and airway $CD8^+$ T_{RM} and $CD4^+$ T_{RM} cells.

Host genotype, boosting interval, and intranasal volume have little effect on Prime and Spike.

To assess whether mouse genotype, boosting interval, or boosting volume affected immunity induced by P&S, we compared mucosal $CD8^+$ T cell and antibody responses following P&S under varying conditions including in K18-hACE2 versus C57B6/J (B6J) mice, 2-week versus 4-week boosting intervals, and 25-μl versus 50-μl intranasal inoculations (fig. S1A). Antigen-specific lung $CD8^+$ T_{RM} cells (fig. S1, B to D), BALF IgA and IgG (fig. S1, E and F), serum IgA and IgG (fig. S1, G and H), and serum neutralizing responses (fig. S1I) were similar among all groups and significantly higher than responses elicited by prime alone. These results support the robustness of P&S as multiple experimental variables can be modified without affecting overall immune responses.

Delayed-interval Prime and Spike induces mucosal immunity.

We wondered whether boosting at an increased interval would affect P&S responses. To test this, mice received IM mRNA-LNP and were boosted with IN spike 84 days later. Humoral and cellular mucosal immune responses on days 91 and 140 were sampled (fig. S2A). Delayed P&S was sufficient to induce $CD8^+$ T_{RM} cells for at least 56 days (fig. S2, B to D). Polyclonal $CD4^+$ T_{RM} cells were induced early at 7 days post boost. However, their numbers appeared to wane by 56 days

(fig. S2, E to G). Delayed P&S also resulted in enhanced mucosal IgA and IgG in BALF (fig. S2, H and I), and serum IgA and IgG (fig. S2, J and K) at 56 days post boost. Thus, P&S administered even up to 3 months after priming elicits durable mucosal humoral and cellular immune responses.

Intranasal delivery of mRNA polyplexes also mediates mucosal boosting.

Poly(amine-co-ester)s (PACE) are biodegradable terpolymers that have been developed to encapsulate and deliver nucleic acids such as mRNA to specified tissues in vivo (*30*). Recent studies have shown that mRNA-LNP delivered to the respiratory tract is lethal in a dose-dependent manner in mice (*31*). By contrast, PACE materials have been developed to be relatively immunologically silent, enabling administration to locations more susceptible to immunopathology such as the respiratory tract. Chemically modifying PACE with polyethylene glycol dramatically improves in vivo lung delivery (*32*). To assess the utility of PACE encapsulating mRNA encoding spike protein as an IN booster, mRNA was extracted from BNT162b2 and encapsulated in PACE. Mice were primed IM with mRNA-LNP and boosted with IN spike mRNA encapsulated in PACE (PACE-spike). Additional control groups included PACE-spike only and IM prime + extracted mRNA (naked mRNA) (fig. S3A). Similar to what we found with P&S, prime and PACE-spike induced antigen-specific $CD8^+$ T_{RM} cells (IV-$CD45^-$$tetramer^+$$CD69^+$$CD103^+$) (fig. S3, B to D). Additionally, PACE-spike-boosted mice developed high levels of BALF IgA. Levels of BALF IgG and serum IgA and IgG were similar to IM prime alone (fig. S3, E to H). IM prime followed by IN naked mRNA was unable to induce mucosal or systemic immune responses above that of IM prime alone indicating that mRNA encapsulation by PACE was required for mucosal boosting. Additionally, a single dose of IN PACE-spike alone was insufficient to elicit any detectable mucosal or systemic antibody response at this dose.

Intranasal Spike or IN PACE-Spike boosts suboptimal Prime to protect against lethal SARS-CoV-2 challenge.

Although current vaccines were initially extremely effective at eliciting protective immunity, waning antibody levels and immune evasion will necessitate boosters for the foreseeable future. The best approach to boosting remains an open question. To test whether IN administration would provide an alternative protective boost, we utilized a low-dose (LD) 0.05 μg of mRNA-LNP vaccine to mimic non-protective immunity. We have previously shown that this dose is insufficient to protect from SARS-CoV-2 challenge despite inducing systemic antibody responses (*15*). Mice primed with LD mRNA-LNP IM and boosted with IN spike developed antigen-specific lung $CD8^+$ T_{RM} cells and IgA and IgG in the BALF at 42 days post boost (fig. S4). Thus, low levels of immune memory allow for effective mucosal boosting of humoral and cellular responses by unadjuvanted IN spike.

Naïve, LD prime only, or LD P&S mice were challenged with SARS-CoV-2 and assessed for viral burden at 2 days post infection (DPI), lungs pathology at 5 DPI, or monitored for weight loss and mortality for 14 days (Fig. 3A). All mice vaccinated with P&S were completely protected from weight loss or death, but neither naïve nor LD prime only mice were protected (Fig. 3, B to D). This protection was accompanied by reduced viral burden in both the upper respiratory tract (nasal turbinates) and lower respiratory tract (lungs) (Fig. 3, E and F). Furthermore, P&S led to significant protection from lung pathology with only one of six mice developing limited mononuclear infiltrates at 5 DPI (Fig. 3, G and H). Next, to assess the protective capacity of PACE-spike IN boost, we again immunized mice with LD mRNA-LNP IM and boosted them IN with PACE-spike mRNA. Prime and PACE-spike resulted in significant protection from morbidity and mortality (Fig. 3, I to L). Thus, P&S represents a robust, versatile, and safe vaccine strategy as intranasal boosting by either IN unadjuvanted spike or PACE-spike is sufficient to induce mucosal immunity and to provide protection against lethal challenge and COVID-19-like pulmonary disease.

Prime and Spike achieves robust systemic booster responses similar to parenteral mRNA-LNP.

IM mRNA-LNP-based boosts are the current standard. Thus, we compared systemic and mucosal immune responses in P&S- and IM mRNA-LNP prime–boost-vaccinated mice (Fig. 4A). Only P&S vaccinated animals developed lung IV-$CD45^-$$tetramer^+$$CD8^+$ T cells that express $CD69^+$ and $CD103^+$ (Fig. 4B to D). The peptide sequence corresponding to spike 62-76 is an epitope recognized by $CD4^+$ T cells in convalescent C57BL/6 mice (*33*). We therefore developed an MHC class II tetramer S62-76 (VTWFHAIHVSGTNGT) that readily identified lung-resident $CD4^+$ T cells in both P&S and convalescent mice (fig. S5). Both infection and vaccination similarly led to increased IV-$CD45^-$$tetramer^+$$CD4^+$$CD69^+$$CD103^-$ T_{RM} cells. P&S induced significantly higher levels of lung-resident antigen-specific $CD4^+$ T cells that phenotypically resemble infection-induced $CD4^+$ T cells (IV-$CD45^-$$tetramer^+$$CD69^+$$CD4^+$) (Fig. 4, E and F). To further characterize the $CD4^+$ T_{RM} cell response, we used a peptide stimulation assay and found that P&S led to higher number of tissue-resident $CD4^+$ T helper type (T_H)1 and T_H17 but not T_H2 $CD4^+$ T cells (Fig. 4, G to K). P&S also led to the induction of polyfunctional lung resident T_H1 cells (fig. S6, B to E).

P&S- but not prime–boost-vaccinated animals developed increased levels of BALF IgA (Fig. 4L). Although BALF IgG levels were increased in prime–boost relative to naïve, P&S developed significantly higher BALF IgG than prime–boost

(Fig. 4M). Serum IgA and IgG in prime–boost- and P&S-vaccinated mice were similar (Fig. 4, N and O), as were neutralizing antibody levels (Fig. 4P). Thus, P&S induces similar systemic binding and neutralizing antibody levels—a correlate of protection in humans—and it uniquely elicits mucosal IgA, IgG, $CD4^+$ T_{RM} cells, and $CD8^+$ T_{RM} cells. Notably, only P&S elicits T_H1 and T_H17 $CD4^+$ T_{RM} cells and not pathogenic T_H2 cell responses, which have been associated with vaccine-associated enhanced disease (VAED) (*34*).

To compare the protective efficacy of P&S to mRNA-LNP prime–boost, mice were primed with LD mRNA-LNP and boosted with either LD mRNA-LNP (IM) or unadjuvanted spike protein (IN). Mice were challenged 118 days post prime. Both vaccine strategies lead to roughly equivalent protection from death with 2/9 prime–boost mice and 0/9 P&S mice succumbing to infection (Fig. 4, Q and R). P&S led to significantly enhanced disease-free survival indicated by only 1/9 mice losing >5% of initial body weight, whereas 6/9 mRNA-LNP prime–boost mice lost >5% of their starting body weight (Fig. 4S). P&S also led enhanced upper airway protection indicated by decreased nasal turbinate viral load and reduced, although not statistically significant lower airway viral load (Fig. 4, T and U).

Prime and Spike reduces transmission in a hamster model of SARS-CoV-2

Next, we used Syrian hamsters to both assess the viability of P&S in an alternate SARS-CoV-2 model and its ability to reduce transmission. Hamsters were vaccinated either by IM mRNA-LNP prime–boost or P&S (Fig. 5A). Serum IgA and IgG levels at 67 days post prime were equivalent between the two groups (Fig. 5, B and C). Hamsters were infected with SARS-CoV-2 at 93 days post prime and both groups were equivalently protected from disease, indicated by minimal weight loss and reduced lung pathology relative to naïve animals (Fig. 5, D and E). P&S-vaccinated animals cleared viral shedding more quickly relative to naïve controls starting at 4 DPI, with all oral swabs negative for infectious virus by 5 DPI. Conversely, mRNA-LNP prime–boost animals did not have significantly lower titers at 4 or 5 DPI and did not stop shedding virus until 6 DPI. Cumulative viral shedding assessed by area under the curve (AUC) revealed that both mRNA-LNP prime–boost and P&S vaccinated animals had significantly lower overall viral shedding than naïve animals. Although P&S AUC was less than mRNA-LNP prime–boost, the results were not statistically significant.

Although P&S reduced viral shedding after infection, whether P&S was able to reduce transmission to vaccinated animals was not yet determined. Vaccinated hamsters were therefore cohoused with naïve donor hamsters who had been infected 24 hours prior (Fig. 5J). P&S vaccinated contact hamsters had significantly lower viral titers at days 2, 4, and 5 post exposure relative to naïve, whereas mRNA prime–boost-vaccinated animals did not have significantly reduced viral shedding at any single time point post exposure (Fig. 5, L to N). Both P&S and mRNA prime–boost were equally protected from lower respiratory tract pathology in the setting of transmission (Fig. 5K and fig. S7). Notably, peak viral load (at 2 DPI) and cumulative viral shedding were significantly reduced in P&S animals relative to both naïve and mRNA-LNP prime–boost contact hamsters (Fig. 5O). Thus, P&S appears to be an effective vaccine strategy in hamsters and reduces viral transmission.

Heterologous spike robustly elicits cross-reactive immunity.

Boosting at a distinct anatomic location—in this case the respiratory mucosa—by homologous unadjuvanted subunit spike enables the formation of new mucosal immune memory and enhances systemic immunity. However, VOCs such as current Omicron sublineages have significant changes to the spike protein sequence, leading to evasion of pre-existing humoral immunity. It is likely that future variants will diverge even more, suggesting a boosting strategy that elicits broadly reactive immunity will be necessary to neutralize future variants.

To test the ability of an unadjuvanted heterologous spike (Spike X) protein in P&S, mice were primed with SARS-CoV-2 mRNA-LNP followed by IN boosting with SARS-CoV-1 spike which we refer to as P&Sx (Fig. 6A). Although SARS-CoV-1 is a related sarbecovirus, its spike protein only shares 76% homology with SARS-CoV-2 spike. At 45 days post prime, there were increased IV-$CD45^-$ $tetramer^+$ $CD8^+$ T_{RM} cells (Fig. 6, B to D). The MHC I tetramer sequence was highly conserved within the sarbecovirus family (fig. S5A). We performed peptide stimulation assay using both SARS-CoV-1 and SARS-CoV-2 peptide pools to assess the development of antigen-specific lung $CD4^+$ T_{RM} cells. We found that P&Sx led to both the development of SARS-CoV-1 and to a lesser extent SARS-CoV-2 specific antigen-specific T_H1 and T_H17 $CD4^+$ T_{RM} cells and no induction of $CD4^+$ T_{RM} expressing the T_H2 cytokine IL-4 (Fig. 6, E to N, and fig. S8). There were also increased anti-SARS-CoV-1 IgA and IgG in both the BALF and serum in P&Sx relative to IM mRNA-LNP prime-boost (Fig. 6, O to R). P&Sx mice correspondingly developed higher neutralization titers against SARS-CoV-1 than mice vaccinated with SARS-CoV-2 mRNA-LNP prime–boost (Fig. 6S). P&Sx induced higher anti-SARS-CoV-2 BALF IgA than SARS-CoV-2 mRNA-LNP prime–boost and similar levels of anti-SARS-CoV-2 IgG in BALF (Fig. 6, T and U). Consistent with the elevated serum IgG levels, mRNA-LNP prime–boost mice had higher serum neutralization titers against SARS-CoV-2 than P&Sx mice (Fig. 6, V to X). Thus, IN boosting with unadjuvanted heterologous spike

protein can induce potent mucosal cellular and humoral memory against a significantly divergent sarbecovirus.

Discussion

Here we describe the preclinical development of an alternative vaccine strategy, P&S, whereby IN unadjuvanted spike subunit protein elicits robust protective mucosal immunity following mRNA-LNP parenteral immunization. These enhanced mucosal responses are characterized by the expansion of antigen-specific CD8$^+$ T_{RM}, CD4$^+$ T_{RM}, and B_{RM} cells as well as mucosal secretion of IgA and IgG. We find that an IN unadjuvanted spike booster can be administered months out from primary immunization and that it offers comparable systemic neutralizing antibody booster responses to IM mRNA-LNP boost. Similarly, Prime and PACE-spike elicits increased antigen-specific CD8$^+$ T_{RM} cells and mucosal IgA. Both boosting methods result in protection from lethal SARS-CoV-2 challenge. We also find that P&S leads to durable responses with protective vaccine efficacy at 118 days from the initiation of vaccination. P&S is protective in hamsters and blocks viral transmission more effectively than mRNA-LNP prime–boost. Finally, by utilizing a divergent spike antigen, we demonstrate that P&Sx can generate mucosal immunity to SARS-CoV-1, while also boosting the systemic and mucosal neutralizing antibodies to the original antigenic target, SARS-CoV-2. Although the goal of vaccination has been to prevent individual morbidity and mortality, the evolution of SARS-CoV-2 has highlighted the need for rapidly deployable mucosal vaccines that also prevent transmission. P&S shows promise in reducing both infection and transmission. Improving upon current vaccine platforms to provide mucosal immunity is vital to control this pandemic and will certainly be important for the next.

Preclinical studies of both SARS-CoV-2 and influenza have demonstrated that intranasal vaccination decreases viral shedding and transmission relative to parenteral vaccines (*19–23*). Despite these studies, there is only one currently approved respiratory mucosal vaccine, FluMist, which relies on a live attenuated influenza virus. FluMist is contraindicated in people with underlying respiratory conditions and is only approved for young people. Additionally, live attenuated vaccines are not amenable to rapid implementation, as this technology require extensive research and development. Accordingly, most current clinical trials of mucosal administered SARS-CoV-2 vaccines rely on either replication-deficient or attenuated viral vectors. However, the safety and efficacy of these approaches have not yet been established, especially given that preexisting immunity to these vectors can lead to reduced immunogenicity (*35*). Indeed, some vector-based mucosal vaccines—including two Merck candidates V590 and V591—have already been abandoned after phase 1 clinical trials showed poor immunogenicity, while candidates by Bharat Biotech and CanSino have recently been approved (*36*).

P&S is likely broadly applicable as a booster against new SARS-CoV-2 VOCs in a previously vaccinated individual or as a de novo primary immunization strategy for newly emerging respiratory pathogens. Although it is possible that our results rely on specific characteristics of mRNA-LNP priming, we believe that this approach will likely work with other primary immunization regimens or in the case of previous infection. Although the above study assesses a single mRNA-LNP dose prior to IN boosting, we would expect unadjuvanted IN boosting to be as effective if not more so in individuals who have received multiple previous shots, because P&S seems to leverage pre-existing immunity rather than be inhibited by it. Additionally, it has been shown that the highly stabilized spike enhances its immunogenicity and that applying this vaccination strategy to other pathogens may require the addition of stabilizing mutations to enable unadjuvanted boosting. Our present study characterizes a method for the development of mucosal immunity to SARS-CoV-2 without the use adjuvants or replicating viruses or vectors in two different well-validated pre-clinical vaccine models. These results are encouraging but require further validation and optimization for human use.

Vaccines that generate broadly neutralizing immunity against a wide variety of sarbecoviruses are a goal to combat both newly emerging SARS-CoV-2 variants and potential pandemic SARS-like coronaviruses. Utilizing SARS-CoV-1 spike as a heterologous IN boost, P&Sx demonstrates that prior SARS-CoV-2 mRNA-LNP does not prevent the development of SARS-CoV-1 neutralizing antibodies, but rather enables it. P&Sx simultaneously elicits broadly reactive neutralizing antibodies and mucosal immunity. Although some recent studies have successfully reported the development of systemic pan-sarbecovirus vaccines (*37*, *38*), P&Sx induces both systemic and mucosal immunity against both SARS-CoV-1 and SARS-CoV-2.

SARS-CoV-2 will continue to evolve and become more immune evasive and transmissible. We will require boosting in human populations for the foreseeable future. Boosting that induces mucosal immunity may help enhance protection and slow transmission as these new variants emerge.

Materials and Methods

All procedures were performed in a BSL-3 facility (for SARS-CoV-2–infected mice) with approval from the Yale Institutional Animal Care and Use Committee and Yale Environmental Health and Safety.

Cell and virus

As reported previously (*15*, *39*, *40*), Vero E6 cells overexpressing ACE2 and TMPRSS2 (kindly provided by B. Graham

at NIH-VRC) were cultured in Dulbecco's Modified Eagle Medium (DMEM) supplemented with 1% sodium pyruvate and 5% fetal bovine serum (FBS) at 37°C and 5% CO_2. SARS-CoV-2 isolate hCOV-19/USA-WA1/2020 (NR-52281) was obtained from BEI Resources and was amplified in VeroE6 cells overexpressing ACE2 and TMPRSS2. Cells were infected at a MOI 0.01 for 2-3 days to generate a working stock and after incubation the supernatant was clarified by centrifugation (5 min, 500*g*) and filtered through a 0.45-μm filter and stored at −80°C. Viral titers were measured by standard plaque assay using Vero E6 cells overexpressing hACE2 and TMPRSS2.

Animals

B6.Cg-Tg(K18-ACE2)2Prlmn/J (K18-hACE2) mice (Stock #: 034860) were purchased from the Jackson Laboratory and subsequently bred and housed at Yale University. Eight-to-twelve-week-old female mice were used for immunization experiments. Male Syrian hamsters (Strain: HSdHan:AURA; Stock #: 089) were purchased from Envigo and vaccination began at 12 weeks of age. All procedures used in this study (e.g., sex matching, age matching, etc.) complied with federal guidelines and the institutional policies of the Yale School of Medicine Animal Care and Use Committee. To reduce the overall number of experimental animals used and to be consistent with our institutional animal use policy, control data points are shared among some figures when applicable and noted in figure legends. Sample sizes for animal experiments were determined empirically based on previously published work in the field with similar experimental paradigms to provide sufficient statistical power for assessing biological effects of interest. No statistical methods were utilized to predetermine the sample size. Age- and sex-matched animals were randomly assigned to experimental groups at the beginning of the experiment. Investigators were not blinded, except for pathological analysis, as no subjective measurements were performed.

SARS-CoV-2 infection

Mice were anesthetized using 30% v/v isoflurane diluted in propylene glycol. Using a pipette, 50 μl containing 6×10^4 PFU SARS-CoV-2 was delivered intranasally. Hamsters were anesthetized using 30% v/v isoflurane diluted in propylene glycol and administered 6×10^3 PFU SARS-CoV-2 intranasally in 100 μl.

mRNA extraction from Comirnaty (BNT162b2) mRNA-LNP

mRNA was extracted from the vaccine formulation with a TRIzol/chloroform separation method as previously described (*41*). Briefly, aliquots of vaccine were dissolved in TRIzol LS (Thermo Fisher Scientific) at 1:6.6 vaccine to TRIzol volume ratio. Following a 15-min incubation (37°C, shaking), 0.2 ml of chloroform was added per 1 ml of TRIzol. The solution was shaken vigorously for 1 min and then incubated at room temperature for 3 min. The solution was centrifuged at 12,000*g* for 8 min at 4°C. The aqueous layer containing the isolated mRNA was further purified with a RNeasy Maxi Kit purchased from Qiagen (Germantown, MD, USA) following the manufacturers protocol. The RNA was eluted from the column on the final step with sodium acetate buffer (25 mM, pH 5.8) warmed to 37°C. Extracted mRNA was analyzed for concentration and purity by NanoDrop measurements of the absorbance at 260, 280 and 230 nm, with purity being assessed as A260/A280 > 2 and A260/A230 > 2. Agarose gel electrophoresis was used to determine the length and verify that the mRNA remained intact. Extracted mRNA containing 1:100 SYBR Safe stain (Thermo Fisher Scientific) was loaded onto a 1% agarose gel and run at 75V with TAE buffer containing 1:5000 SYBR Safe stain (fig. S9).

PACE polyplex formulation and characterization

PACE polymers were synthesized and characterized as previously described (*42*). All polyplexes were formulated at a 50:1 weight ratio of polymer to mRNA. PACE polymers were dissolved at 100 mg/ml overnight in DMSO (37°C, shaking). Prior to polyplex fabrication, an optimal PACE polymer blend was produced by mixing solutions of PACE polymers containing an end-group modification (*43*) and a polyethylene glycol tail (*30*). mRNA and polymer were diluted into equal volumes of sodium acetate buffer (25 mM, pH 5.8). The polymer dilution was then vortexed for 15 s, mixed with the mRNA dilution, and vortexed for an additional 25 s. Polyplexes were incubated at room temperature for 10 min before use.

Vaccination

Used vials of Comirnaty vaccine were acquired from Yale Health pharmacy within 24 hours of opening and stored at 4°C. Vials contained residual vaccine (diluted to 100 μg/ml per manufacturer's instructions) which was removed with spinal syringe and pooled. Pooled residual vaccine was aliquoted and stored at −80°C. Mice were anesthetized using a mixture of ketamine (50 mg per kilogram of body weight) and xylazine (5 mg per kilogram of body weight) and injected intraperitoneally. Vaccine was diluted in sterile PBS and 10 μl or 20 μl was injected into the left quadriceps muscle with a 31G syringe for a final dose of 1 μg or 0.05 μg as indicated. Similarly, hamsters were administered 0.5 μg diluted in 20 μl via 31G syringe in the left quadriceps muscle. For intranasal vaccination, SARS-CoV-2 stabilized spike (ACRO biosystems, SPN-C52H9) or SARS-CoV-1 spike (ACRO biosystems, SPN-S52H6) was reconstituted in sterile endotoxin-free water according to the manufacturer's protocol, and then diluted in sterile PBS and stored at −80°C. Mice or hamsters were anesthetized using isoflurane and administered 1 μg or 5 μg (as

indicated) in 50 µl (25 µl where indicated) via the IN route. For IN mRNA-PACE, 50 µl of polyplexes in solution was administered at the indicated dose.

Viral titer analysis

Viral titer analysis was performed as previously described (*15*, *39*, *40*) with modifications noted and summarized here. Mice were euthanized in 100% isoflurane at indicated time points. Approximately half of the total lung (right lobes) or nasal turbinate was homogenized in a bead homogenizer tube containing 1 ml of PBS supplemented with 2% FBS and 2% antibiotics/antimycotics (Gibco) and stored at −80°C. Nasal turbinate and lung homogenates were clarified of debris by centrifugation (10 min, 3100*g*). Daily oral swabs (Pruitan PurFlock Ultra 25-3206-U) were performed on hamsters and stored in 1 ml of DMEM with 2% FBS and 2% antibiotics/antimycotics (Gibco) and stored at −80°C. To determine infectious SARS-CoV-2 titers, plaque assay was performed using ACE2- and TMPRSS2-overexpressing VeroE6 cells. Plaques were resolved by formalin fixation 40-42 hours after infection, followed by staining with crystal violet and rinsing with water for plaque visualization.

SARS-CoV-2 specific-antibody measurements

ELISAs were performed as previously described (*39*, *44*) with modifications noted and summarized here. Ninety-six-well MaxiSorp plates (Thermo Scientific #442404) were coated with recombinant SARS-CoV-2 S1 protein (ACRO Biosystems S1N-C52H3) or SARS-CoV-1 S1 protein (ACRO Biosystems S1N-S52H5). Following overnight incubation at 4°C, plates were replaced with blocking solution (PBS with 0.1% Tween-20, and 5% milk powder) and incubated for 1-2 hours at RT. Serum or BALF was diluted in dilution solution (PBS with 0.1% Tween-20 and 2% milk powder) and added to plates for 2 hours at RT. Plates were washed five times with PBS-T (PBS with 0.05% Tween-20) using an automatic plate washer (250 µl per cycle) and 50 µl of HRP anti-mouse IgG (Cell Signaling Technology #7076, 1:3,000), HRP anti-mouse IgA (Southern Biotech #1040-05, 1:1,000), HRP anti-hamster IgG (Southern Biotech #6060-05, 1:1,000), or rabbit anti-hamster IgA HRP (Brookwood Biomedical, sab3003a, 1:250 100 µg/ml) diluted in dilution solution was added to each well. After 1 hour of incubation at RT (overnight at 4°C for hamster IgA), plates were washed three times with PBS-T using an automatic plate washer. Fifty microliters of TMB Substrate Reagent Set (BD Biosciences #555214) was added to plates. To terminate the reaction, another 50 µl of 2 N sulfuric acid was added after 15 min of substrate development. Plates were then recorded at wavelengths of 450 nm and 570 nm and the difference was reported as area under the curve (AUC).

Immunohistochemistry and pathological analysis

Yale Pathology Tissue Services (YPTS) performed embedding, sectioning, and H&E staining of lung tissue. A pulmonary pathologist reviewed the slides blinded and identified immune cell infiltration and other related pathologies. Mouse lung scores of 1-4 were characterized as follows: (1) mild patchy mononuclear infiltrate, parenchymal and perivascular, with variably reactive pneumocytes and stromal rection; (2) moderate patchy mononuclear infiltrate, parenchymal and perivascular, with variably reactive pneumocytes and stromal rection; (3) mild, dense mixed infiltrate including mononuclear cells and granulocytes/neutrophils; and (4) moderate, dense mixed infiltrate including mononuclear cells and granulocytes/neutrophils. Hamster lung scores of 0-4 were characterized as follows: (0) normal; (1) very focal injury/inflammation/repair; (2) multifocal repair; and (4) multifocal repair with necrosis.

Intravascular labeling, cell isolation, and flow cytometry

To discriminate circulating from extravascular immune cells, mice were anesthetized with 30% isoflurane and injected IV with 2 µg of APC/Fire 750-labeled anti-CD45 Ab. After 3 min of labeling, mice were euthanized. Tissues were harvested and analyzed as previously described (*39*). Briefly, lungs and nasal turbinates were minced with scissors, incubated in a digestion cocktail containing collagenase A (Roche) and DNase I (Sigma-Aldrich) in RPMI at 37°C for 45 min, and dissociated through a 70-µm filter. Airway-resident immune cells were collected by centrifuging BALF at 600*g* for 5 min at 4°C, after which cell pellets were used for flow cytometry and supernatants were used for antibody analysis. Cells were treated with ammonium-chloride-potassium (ACK) buffer to lyse red blood cells and then washed once with PBS. Single-cell suspensions were incubated with Fixable Aqua cell viability dye (Invitrogen L34957) and anti-mouse CD16/CD32 Fc Block (BD Biosciences 553141) for 30 min at 4°C. Cells were washed once with PBS before surface staining. For T cell analysis, cells were first stained with APC-labeled SARS-CoV-2 S 62-76 MHC class II tetramer (I-A(b)) for 60 min at RT. Cells were washed once with PBS, and then stained with anti-CD103, anti-CD3, anti-CD44, anti-CD62L, anti-CD8a, anti-CD69, anti-CD183 (CXCR3), anti-CD4, and PE-SARS-CoV-2 S 539-546 MHC class I tetramer (H-2K(b)) for 30 min at 4°C. For B cell analysis, cells were stained with ant-GL7, anti-IgM, anti-CD138, anti-CD19, anti-IgA, anti-B220, PE-SARS-CoV-2 RBD tetramer, anti-CD38, APC-SARS-CoV-2 RBD tetramer, and anti-IgD for 30 min at 4°C. Cells were washed with PBS once, following by 4% paraformaldehyde fixation for 45 min at 4°C. Flow cytometry data were acquired on an Attune NxT Flow Cytometer and analyzed using FlowJo

Software (10.5.3; Tree Star). See fig. S10 for gating strategy and table S1 for detailed antibody information.

Intracellular cytokine staining assay for detection of lung-resident spike-specific CD4 T cells

Following intravascular labeling using an anti-CD45 Ab at the dose of 2 μg per mouse, lung isolation, and processing, single cells from the lung tissue was first enriched using a Percoll gradient prior to spike peptide stimulation. Briefly, total lung cells were first resuspended in 5 ml of 30% Percoll solution in a 15 ml conical tube, underlaid with 5 ml of 70% Percoll solution, and subject to centrifugation at 1000*g* for 20 min at RT. Following centrifugation, lymphocytes located at the interphase between 30% and 70% Percoll solution were collected, washed once with PBS, and resuspended in complete RPMI. In a 96-well U-bottom plate, 10^6 lymphocytes enriched from each lung sample were added, together with spike peptide megapool from SARS-CoV-2 (JPT PM-WCPV-S-1) or SARS-CoV-1 (JPT PM-CVHSA-S-1) at a final working concentration of 1 μg/ml per peptide, 1X Protein Transport Inhibitor Cocktail (eBioscience 00-4980-03), and 10^6 freshly isolated splenocytes from CD45.1$^+$ mice, with complete RPMI for a final volume of 200 μl. Peptide stimulation was performed for 8 hours at 37°C. Following peptide stimulation, cells were incubated at 4°C with Fc block (BioXCell BE0307) and Aqua cell viability dye (ThermoFisher L34957) for 20 min. Cells were washed once with PBS before surface staining with anti-CD3, anti-CD44, anti-CD4, and anti-CD45.1. After washing with PBS, cells were fixed using 4% paraformaldehyde for 45 min at 4°C. Cells were then washed and permeabilized with 1X Permeabilization Buffer (eBioscience 00-8333-56) for 10 min at RT. Following permeabilization, cells were stained with anti-IL-4, anti-IL-2, anti-TNF-α, anti-IL-17A, and anti-IFN-γ. Cells were washed once with PBS before being acquired on Attune and analyzed using FlowJo. See fig. S10 for gating strategy and table S1 for detailed antibody information.

SARS-CoV-2 receptor-binding domain B cell tetramer production and staining

Recombinant SARS-CoV-2 Spike RBD His Biotin Protein, CF (R&D BT10500-050) was incubated at a 4:1 molar ratio with either streptavidin-PE (Prozyme PJRS25) or streptavidin-APC (Prozyme PJ27S) for 30 min at 4°C. Mixture was then purified and concentrated in an Amicon Ultra (50 kDA MWCO) spin column and washed 1X with sterile cold PBS. The concentration was determined on a NanoDrop 8000 Spectrophotometer (ThermoFisher ND-8000-GL) using fluorophore-specific absorbances. Tetramers were then diluted to 1.0 μM in PBS and stored at 4°C. For every 2-5×10^7 cells, 1 μl of stock 1.0 μM tetramer was used for staining.

Pseudovirus production and neutralization assay

Pseudoviruses were produced as previously described (*15*). Spike-encoding plasmid was kindly provided by Dr. V. Munster and previously described (*45*). To perform pseudovirus neutralization assays, VeroE6 overexpressing hACE2 and TMPRSS2 (Fig. 1) or Huh7.5 cell (Fig. 5 and fig. S3) were plated (3×10^4) in each well of a white 96-well plate the day before infection. On the day of infection, serum and BALF were heat-inactivated for 30 min at 56°C. Figure 1 sera were tested at a starting dilution of 1:50 and BALF samples were tested at a starting dilution of 1:4, both with eight twofold serial dilutions. Sera shown in Fig. 5 and fig. S3 were tested at a starting dilution of 1:40 with eight threefold serial dilutions. Serial dilutions mixed 1:1 with indicated pseudovirus and incubated for 1 hour at 37°C and 5% CO_2. Growth medium was then aspirated from the cells and replaced with 100 μl of serum/virus mixture. 24 HPI infection/antibody mixture was removed and plates flash-frozen at −80°C. Thirty microliters of passive lysis buffer (Promega) was added to each well and plates were incubated for 15 min at RT. Thirty microliters of Renilla-Glo Luciferase Assay System substrate (Promega) was then added to each well and incubated at RT for an additional 15 min. Luminescence was measured on a microplate reader (SpectraMax i3, Molecular Devices). IC50 was calculated as using Prism 9 (GraphPad Software) nonlinear regression.

Sequence alignment

The following amino acid sequences of coronavirus Spike proteins used in alignment were obtained from Uniprot/Genebank: Wuhan (P0DTC2), B.1.1.7 (QWE88920.1), B.1.351 (QRN78347.1), B.1.617 (QUD52764.1), B.1.1.28.1 (QRX39425.1), BA.1 (UFO69279.1), BA.2 (UFO69279.1), BA.2.12.1 (UMZ92892.1), BA.4 (UPP14409.1), BA.5 (UOZ45804.1), Khosta (MZ190137.1), Khosta-2 (MZ190138.1), SARS-CoV (AY278489.2), WIV1 (KF367457), and BANAL236 (MZ937003.1). Sequence alignment was performed with MAFFT in JalView (v2.11.2.3).

Graphical illustrations

Graphical illustrations were made with Biorender.com.

REFERENCES AND NOTES

1. L. R. Baden, H. M. El Sahly, B. Essink, K. Kotloff, S. Frey, R. Novak, D. Diemert, S. A. Spector, N. Rouphael, C. B. Creech, J. McGettigan, S. Khetan, N. Segall, J. Solis, A. Brosz, C. Fierro, H. Schwartz, K. Neuzil, L. Corey, P. Gilbert, H. Janes, D. Follmann, M. Marovich, J. Mascola, L. Polakowski, J. Ledgerwood, B. S. Graham, H. Bennett, R. Pajon, C. Knightly, B. Leav, W. Deng, H. Zhou, S. Han, M. Ivarsson, J. Miller, T. Zaks; COVE Study Group, Efficacy and Safety of the mRNA-1273 SARS-CoV-2 Vaccine. *N. Engl. J. Med.* **384**, 403–416 (2021). doi:10.1056/NEJMoa2035389 Medline
2. N. Dagan, N. Barda, E. Kepten, O. Miron, S. Perchik, M. A. Katz, M. A. Hernán, M. Lipsitch, B. Reis, R. D. Balicer, BNT162b2 mRNA Covid-19 Vaccine in a Nationwide Mass Vaccination Setting. *N. Engl. J. Med.* **384**, 1412–1423 (2021). doi:10.1056/NEJMoa2101765 Medline

3. K. R. W. Emary, T. Golubchik, P. K. Aley, C. V. Ariani, B. Angus, S. Bibi, B. Blane, D. Bonsall, P. Cicconi, S. Charlton, E. A. Clutterbuck, A. M. Collins, T. Cox, T. C. Darton, C. Dold, A. D. Douglas, C. J. A. Duncan, K. J. Ewer, A. L. Flaxman, S. N. Faust, D. M. Ferreira, S. Feng, A. Finn, P. M. Folegatti, M. Fuskova, E. Galiza, A. L. Goodman, C. M. Green, C. A. Green, M. Greenland, B. Hallis, P. T. Heath, J. Hay, H. C. Hill, D. Jenkin, S. Kerridge, R. Lazarus, V. Libri, P. J. Lillie, C. Ludden, N. G. Marchevsky, A. M. Minassian, A. C. McGregor, Y. F. Mujadidi, D. J. Phillips, E. Plested, K. M. Pollock, H. Robinson, A. Smith, R. Song, M. D. Snape, R. K. Sutherland, E. C. Thomson, M. Toshner, D. P. J. Turner, J. Vekemans, T. L. Villafana, C. J. Williams, A. V. S. Hill, T. Lambe, S. C. Gilbert, M. Voysey, M. N. Ramasamy, A. J. Pollard; COVID-19 Genomics UK consortium; AMPHEUS Project; Oxford COVID-19 Vaccine Trial Group, Efficacy of ChAdOx1 nCoV-19 (AZD1222) vaccine against SARS-CoV-2 variant of concern 202012/01 (B.1.1.7): An exploratory analysis of a randomised controlled trial. *Lancet* **397**, 1351–1362 (2021). doi:10.1016/S0140-6736(21)00628-0 Medline
4. A. S. V. Shah, C. Gribben, J. Bishop, P. Hanlon, D. Caldwell, R. Wood, M. Reid, J. McMenamin, D. Goldberg, D. Stockton, S. Hutchinson, C. Robertson, P. M. McKeigue, H. M. Colhoun, D. A. McAllister, Effect of Vaccination on Transmission of SARS-CoV-2. *N. Engl. J. Med.* **385**, 1718–1720 (2021). doi:10.1056/NEJMc2106757 Medline
5. R. J. Harris, J. A. Hall, A. Zaidi, N. J. Andrews, J. K. Dunbar, G. Dabrera, Effect of Vaccination on Household Transmission of SARS-CoV-2 in England. *N. Engl. J. Med.* **385**, 759–760 (2021). doi:10.1056/NEJMc2107717 Medline
6. H. Chemaitelly, P. Tang, M. R. Hasan, S. AlMukdad, H. M. Yassine, F. M. Benslimane, H. A. Al Khatib, P. Coyle, H. H. Ayoub, Z. Al Kanaani, E. Al Kuwari, A. Jeremijenko, A. H. Kaleeckal, A. N. Latif, R. M. Shaik, H. F. Abdul Rahim, G. K. Nasrallah, M. G. Al Kuwari, H. E. Al Romaihi, A. A. Butt, M. H. Al-Thani, A. Al Khal, R. Bertollini, L. J. Abu-Raddad, Waning of BNT162b2 Vaccine Protection against SARS-CoV-2 Infection in Qatar. *N. Engl. J. Med.* **385**, e83 (2021). doi:10.1056/NEJMoa2114114 Medline
7. B. A. Cohn, P. M. Cirillo, C. C. Murphy, N. Y. Krigbaum, A. W. Wallace, SARS-CoV-2 vaccine protection and deaths among US veterans during 2021. *Science* **375**, 331–336 (2022). doi:10.1126/science.abm0620 Medline
8. C. Lucas, C. B. F. Vogels, I. Yildirim, J. E. Rothman, P. Lu, V. Monteiro, J. R. Gehlhausen, M. Campbell, J. Silva, A. Tabachnikova, M. A. Peña-Hernandez, M. C. Muenker, M. I. Breban, J. R. Fauver, S. Mohanty, J. Huang, A. C. Shaw, A. I. Ko, S. B. Omer, N. D. Grubaugh, A. Iwasaki, Yale SARS-CoV-2 Genomic Surveillance Initiative, Impact of circulating SARS-CoV-2 variants on mRNA vaccine-induced immunity. *Nature* **600**, 523–529 (2021). doi:10.1038/s41586-021-04085-y Medline
9. F. Schmidt, F. Muecksch, Y. Weisblum, J. Da Silva, E. Bednarski, A. Cho, Z. Wang, C. Gaebler, M. Caskey, M. C. Nussenzweig, T. Hatziioannou, P. D. Bieniasz, Plasma neutralization of the SARS-CoV-2 Omicron variant. *N. Engl. J. Med.* **386**, 599–601 (2022). doi:10.1056/NEJMc2119641 Medline
10. S. Cele *et al.*, SARS-CoV-2 Omicron has extensive but incomplete escape of Pfizer BNT162b2 elicited neutralization and requires ACE2 for infection. *Nature* **602**, 654–656 (2022). doi:10.1038/s41586-021-04387-1 Medline
11. A. Singanayagam, S. Hakki, J. Dunning, K. J. Madon, M. A. Crone, A. Koycheva, N. Derqui-Fernandez, J. L. Barnett, M. G. Whitfield, R. Varro, A. Charlett, R. Kundu, J. Fenn, J. Cutajar, V. Quinn, E. Conibear, W. Barclay, P. S. Freemont, G. P. Taylor, S. Ahmad, M. Zambon, N. M. Ferguson, A. Lalvani, A. Badhan, S. Dustan, C. Tejpal, A. V. Ketkar, J. S. Narean, S. Hammett, E. McDermott, T. Pillay, H. Houston, C. Luca, J. Samuel, S. Bremang, S. Evetts, J. Poh, C. Anderson, D. Jackson, S. Miah, J. Ellis, A. Lackenby; ATACCC Study Investigators, Community transmission and viral load kinetics of the SARS-CoV-2 delta (B.1.617.2) variant in vaccinated and unvaccinated individuals in the UK: A prospective, longitudinal, cohort study. *Lancet Infect. Dis.* **22**, 183–195 (2022). doi:10.1016/S1473-3099(21)00648-4 Medline
12. D. Laczkó, M. J. Hogan, S. A. Toulmin, P. Hicks, K. Lederer, B. T. Gaudette, D. Castaño, F. Amanat, H. Muramatsu, T. H. Oguin 3rd, A. Ojha, L. Zhang, Z. Mu, R. Parks, T. B. Manzoni, B. Roper, S. Strohmeier, I. Tombácz, L. Arwood, R. Nachbagauer, K. Karikó, J. Greenhouse, L. Pessaint, M. Porto, T. Putman-Taylor, A. Strasbaugh, T.-A. Campbell, P. J. C. Lin, Y. K. Tam, G. D. Sempowski, M. Farzan, H. Choe, K. O. Saunders, B. F. Haynes, H. Andersen, L. C. Eisenlohr, D. Weissman, F. Krammer, P. Bates, D. Allman, M. Locci, N. Pardi, A Single Immunization with Nucleoside-Modified mRNA Vaccines Elicits Strong Cellular and Humoral Immune Responses against SARS-CoV-2 in Mice. *Immunity* **53**, 724–732.e7 (2020). doi:10.1016/j.immuni.2020.07.019 Medline
13. R. R. Goel, M. M. Painter, S. A. Apostolidis, D. Mathew, W. Meng, A. M. Rosenfeld, K. A. Lundgreen, A. Reynaldi, D. S. Khoury, A. Pattekar, S. Gouma, L. Kuri-Cervantes, P. Hicks, S. Dysinger, A. Hicks, H. Sharma, S. Herring, S. Korte, A. E. Baxter, D. A. Oldridge, J. R. Giles, M. E. Weirick, C. M. McAllister, M. Awofolaju, N. Tanenbaum, E. M. Drapeau, J. Dougherty, S. Long, K. D'Andrea, J. T. Hamilton, M. McLaughlin, J. C. Williams, S. Adamski, O. Kuthuru, I. Frank, M. R. Betts, L. A. Vella, A. Grifoni, D. Weiskopf, A. Sette, S. E. Hensley, M. P. Davenport, P. Bates, E. T. Luning Prak, A. R. Greenplate, E. J. Wherry; UPenn COVID Processing Unit‡, mRNA vaccines induce durable immune memory to SARS-CoV-2 and variants of concern. *Science* **374**, abm0829 (2021). doi:10.1126/science.abm0829 Medline
14. J. S. Turner, J. A. O'Halloran, E. Kalaidina, W. Kim, A. J. Schmitz, J. Q. Zhou, T. Lei, M. Thapa, R. E. Chen, J. B. Case, F. Amanat, A. M. Rauseo, A. Haile, X. Xie, M. K. Klebert, T. Suessen, W. D. Middleton, P.-Y. Shi, F. Krammer, S. A. Teefey, M. S. Diamond, R. M. Presti, A. H. Ellebedy, SARS-CoV-2 mRNA vaccines induce persistent human germinal centre responses. *Nature* **596**, 109–113 (2021). doi:10.1038/s41586-021-03738-2 Medline
15. B. Israelow, T. Mao, J. Klein, E. Song, B. Menasche, S. B. Omer, A. Iwasaki, Adaptive immune determinants of viral clearance and protection in mouse models of SARS-CoV-2. *Sci. Immunol.* **6**, eabl4509 (2021). doi:10.1126/sciimmunol.abl4509 Medline
16. S. Sheikh-Mohamed, B. Isho, G. Y. C. Chao, M. Zuo, C. Cohen, Y. Lustig, G. R. Nahass, R. E. Salomon-Shulman, G. Blacker, M. Fazel-Zarandi, B. Rathod, K. Colwill, A. Jamal, Z. Li, K. Q. de Launay, A. Takaoka, J. Garnham-Takaoka, A. Patel, C. Fahim, A. Paterson, A. X. Li, N. Haq, S. Barati, L. Gilbert, K. Green, M. Mozafarihashjin, P. Samaan, P. Budylowski, W. L. Siqueira, S. Mubareka, M. Ostrowski, J. M. Rini, O. L. Rojas, I. L. Weissman, M. C. Tal, A. McGeer, G. Regev-Yochay, S. Straus, A.-C. Gingras, J. L. Gommerman, Systemic and mucosal IgA responses are variably induced in response to SARS-CoV-2 mRNA vaccination and are associated with protection against subsequent infection. *Mucosal Immunol.* **15**, 799–808 (2022). doi:10.1038/s41385-022-00511-0 Medline
17. K. Sano *et al.*, Efficient mucosal antibody response to SARS-CoV-2 vaccination is induced in previously infected individuals. medRxiv 2021.2012.2006.21267352 [Preprint] (2021). https://doi.org/10.1101/2021.12.06.21267352.
18. M. M. L. Poon, K. Rybkina, Y. Kato, M. Kubota, R. Matsumoto, N. I. Bloom, Z. Zhang, K. M. Hastie, A. Grifoni, D. Weiskopf, S. B. Wells, B. B. Ural, N. Lam, P. A. Szabo, P. Dogra, Y. S. Lee, J. I. Gray, M. C. Bradley, M. A. Brusko, T. M. Brusko, E. O. Saphire, T. J. Connors, A. Sette, S. Crotty, D. L. Farber, SARS-CoV-2 infection generates tissue-localized immunological memory in humans. *Sci. Immunol.* **6**, eabl9105 (2021). doi:10.1126/sciimmunol.abl9105 Medline
19. A. O. Hassan, N. M. Kafai, I. P. Dmitriev, J. M. Fox, B. K. Smith, I. B. Harvey, R. E. Chen, E. S. Winkler, A. W. Wessel, J. B. Case, E. Kashentseva, B. T. McCune, A. L. Bailey, H. Zhao, L. A. VanBlargan, Y.-N. Dai, M. Ma, L. J. Adams, S. Shrihari, J. E. Danis, L. E. Gralinski, Y. J. Hou, A. Schäfer, A. S. Kim, S. P. Keeler, D. Weiskopf, R. S. Baric, M. J. Holtzman, D. H. Fremont, D. T. Curiel, M. S. Diamond, A Single-Dose Intranasal ChAd Vaccine Protects Upper and Lower Respiratory Tracts against SARS-CoV-2. *Cell* **183**, 169–184.e13 (2020). doi:10.1016/j.cell.2020.08.026 Medline
20. A. O. Hassan, F. Feldmann, H. Zhao, D. T. Curiel, A. Okumura, T.-L. Tang-Huau, J. B. Case, K. Meade-White, J. Callison, R. E. Chen, J. Lovaglio, P. W. Hanley, D. P. Scott, D. H. Fremont, H. Feldmann, M. S. Diamond, A single intranasal dose of chimpanzee adenovirus-vectored vaccine protects against SARS-CoV-2 infection in rhesus macaques. *Cell Rep. Med.* **2**, 100230 (2021). doi:10.1016/j.xcrm.2021.100230 Medline
21. T. L. Bricker, T. L. Darling, A. O. Hassan, H. H. Harastani, A. Soung, X. Jiang, Y.-N. Dai, H. Zhao, L. J. Adams, M. J. Holtzman, A. L. Bailey, J. B. Case, D. H. Fremont, R. Klein, M. S. Diamond, A. C. M. Boon, A single intranasal or intramuscular immunization with chimpanzee adenovirus-vectored SARS-CoV-2 vaccine protects against pneumonia in hamsters. *Cell Rep.* **36**, 109400 (2021). doi:10.1016/j.celrep.2021.109400 Medline
22. R. J. Fischer, N. van Doremalen, D. R. Adney, C. K. Yinda, J. R. Port, M. G. Holbrook, J. E. Schulz, B. N. Williamson, T. Thomas, K. Barbian, S. L. Anzick, S. Ricklefs, B. J. Smith, D. Long, C. Martens, G. Saturday, E. de Wit, S. C. Gilbert, T. Lambe, V. J.

Munster, ChAdOx1 nCoV-19 (AZD1222) protects Syrian hamsters against SARS-CoV-2 B.1.351 and B.1.1.7. *Nat. Commun.* **12**, 5868 (2021). doi:10.1038/s41467-021-26178-y Medline
23. N. van Doremalen, J. N. Purushotham, J. E. Schulz, M. G. Holbrook, T. Bushmaker, A. Carmody, J. R. Port, C. K. Yinda, A. Okumura, G. Saturday, F. Amanat, F. Krammer, P. W. Hanley, B. J. Smith, J. Lovaglio, S. L. Anzick, K. Barbian, C. Martens, S. C. Gilbert, T. Lambe, V. J. Munster, Intranasal ChAdOx1 nCoV-19/AZD1222 vaccination reduces viral shedding after SARS-CoV-2 D614G challenge in preclinical models. *Sci. Transl. Med.* **13**, eabh0755 (2021). doi:10.1126/scitranslmed.abh0755 Medline
24. J. Tang, C. Zeng, T. M. Cox, C. Li, Y. M. Son, I. S. Cheon, Y. Wu, S. Behl, J. J. Taylor, R. Chakaraborty, A. J. Johnson, D. N. Shiavo, J. P. Utz, J. S. Reisenauer, D. E. Midthun, J. J. Mullon, E. S. Edell, M. G. Alameh, L. Borish, W. G. Teague, M. H. Kaplan, D. Weissman, R. Kern, H. Hu, R. Vassallo, S.-L. Liu, J. Sun, Respiratory mucosal immunity against SARS-CoV-2 after mRNA vaccination. *Sci. Immunol.* **7**, eadd4853 (2022). doi:10.1126/sciimmunol.add4853 Medline
25. F. Fiorino, E. Pettini, G. Pozzi, D. Medaglini, A. Ciabattini, Prime-boost strategies in mucosal immunization affect local IgA production and the type of th response. *Front. Immunol.* **4**, 128 (2013). doi:10.3389/fimmu.2013.00128 Medline
26. D. Lapuente, J. Fuchs, J. Willar, A. Vieira Antão, V. Eberlein, N. Uhlig, L. Issmail, A. Schmidt, F. Oltmanns, A. S. Peter, S. Mueller-Schmucker, P. Irrgang, K. Fraedrich, A. Cara, M. Hoffmann, S. Pöhlmann, A. Ensser, C. Pertl, T. Willert, C. Thirion, T. Grunwald, K. Überla, M. Tenbusch, Protective mucosal immunity against SARS-CoV-2 after heterologous systemic prime-mucosal boost immunization. *Nat. Commun.* **12**, 6871 (2021). doi:10.1038/s41467-021-27063-4 Medline
27. M. Mutsch, W. Zhou, P. Rhodes, M. Bopp, R. T. Chen, T. Linder, C. Spyr, R. Steffen, Use of the inactivated intranasal influenza vaccine and the risk of Bell's palsy in Switzerland. *N. Engl. J. Med.* **350**, 896–903 (2004). doi:10.1056/NEJMoa030595 Medline
28. C.-L. Hsieh, J. A. Goldsmith, J. M. Schaub, A. M. DiVenere, H.-C. Kuo, K. Javanmardi, K. C. Le, D. Wrapp, A. G. Lee, Y. Liu, C.-W. Chou, P. O. Byrne, C. K. Hjorth, N. V. Johnson, J. Ludes-Meyers, A. W. Nguyen, J. Park, N. Wang, D. Amengor, J. J. Lavinder, G. C. Ippolito, J. A. Maynard, I. J. Finkelstein, J. S. McLellan, Structure-based design of prefusion-stabilized SARS-CoV-2 spikes. *Science* **369**, 1501–1505 (2020). doi:10.1126/science.abd0826 Medline
29. S. R. Allie, J. E. Bradley, U. Mudunuru, M. D. Schultz, B. A. Graf, F. E. Lund, T. D. Randall, The establishment of resident memory B cells in the lung requires local antigen encounter. *Nat. Immunol.* **20**, 97–108 (2019). doi:10.1038/s41590-018-0260-6 Medline
30. M. K. Grun, A. Suberi, K. Shin, T. Lee, V. Gomerdinger, Z. M. Moscato, A. S. Piotrowski-Daspit, W. M. Saltzman, PEGylation of poly(amine-co-ester) polyplexes for tunable gene delivery. *Biomaterials* **272**, 120780 (2021). doi:10.1016/j.biomaterials.2021.120780 Medline
31. S. Ndeupen, Z. Qin, S. Jacobsen, A. Bouteau, H. Estanbouli, B. Z. Igyártó, The mRNA-LNP platform's lipid nanoparticle component used in preclinical vaccine studies is highly inflammatory. *iScience* **24**, 103479 (2021). doi:10.1016/j.isci.2021.103479 Medline
32. A. Suberi *et al.*, Inhalable polymer nanoparticles for versatile mRNA delivery and mucosal vaccination. bioRxiv, 485401 [Preprint] (2022). https://doi.org/10.1101/2022.03.22.485401.
33. Z. Zhuang, X. Lai, J. Sun, Z. Chen, Z. Zhang, J. Dai, D. Liu, Y. Li, F. Li, Y. Wang, A. Zhu, J. Wang, W. Yang, J. Huang, X. Li, L. Hu, L. Wen, J. Zhuo, Y. Zhang, D. Chen, S. Li, S. Huang, Y. Shi, K. Zheng, N. Zhong, J. Zhao, D. Zhou, J. Zhao, Mapping and role of T cell response in SARS-CoV-2-infected mice. *J. Exp. Med.* **218**, e20202187 (2021). doi:10.1084/jem.20202187 Medline
34. C. Gartlan, T. Tipton, F. J. Salguero, Q. Sattentau, A. Gorringe, M. W. Carroll, Vaccine-Associated Enhanced Disease and Pathogenic Human Coronaviruses. *Front. Immunol.* **13**, 882972 (2022). doi:10.3389/fimmu.2022.882972 Medline
35. S. Sanchez, N. Palacio, T. Dangi, T. Ciucci, P. Penaloza-MacMaster, Fractionating a COVID-19 Ad5-vectored vaccine improves virus-specific immunity. *Sci. Immunol.* **6**, eabi8635 (2021). doi:10.1126/sciimmunol.abi8635 Medline
36. M. P. Release, Merck Discontinues Development of SARS-CoV-2/COVID-19 Vaccine Candidates; Continues Development of Two Investigational Therapeutic Candidates https://www.merck.com/news/merck-discontinues-development-of-sars-cov-2-covid-19-vaccine-candidates-continues-development-of-two-investigational-therapeutic-candidates (2021).
37. D. R. Martinez, A. Schäfer, S. R. Leist, G. De la Cruz, A. West, E. N. Atochina-Vasserman, L. C. Lindesmith, N. Pardi, R. Parks, M. Barr, D. Li, B. Yount, K. O. Saunders, D. Weissman, B. F. Haynes, S. A. Montgomery, R. S. Baric, Chimeric spike mRNA vaccines protect against Sarbecovirus challenge in mice. *Science* **373**, 991–998 (2021). doi:10.1126/science.abi4506 Medline
38. A. C. Walls, M. C. Miranda, A. Schäfer, M. N. Pham, A. Greaney, P. S. Arunachalam, M.-J. Navarro, M. A. Tortorici, K. Rogers, M. A. O'Connor, L. Shirreff, D. E. Ferrell, J. Bowen, N. Brunette, E. Kepl, S. K. Zepeda, T. Starr, C.-L. Hsieh, B. Fiala, S. Wrenn, D. Pettie, C. Sydeman, K. R. Sprouse, M. Johnson, A. Blackstone, R. Ravichandran, C. Ogohara, L. Carter, S. W. Tilles, R. Rappuoli, S. R. Leist, D. R. Martinez, M. Clark, R. Tisch, D. T. O'Hagan, R. Van Der Most, W. C. Van Voorhis, D. Corti, J. S. McLellan, H. Kleanthous, T. P. Sheahan, K. D. Smith, D. H. Fuller, F. Villinger, J. Bloom, B. Pulendran, R. S. Baric, N. P. King, D. Veesler, Elicitation of broadly protective sarbecovirus immunity by receptor-binding domain nanoparticle vaccines. *Cell* **184**, 5432–5447.e16 (2021). doi:10.1016/j.cell.2021.09.015 Medline
39. B. Israelow, E. Song, T. Mao, P. Lu, A. Meir, F. Liu, M. M. Alfajaro, J. Wei, H. Dong, R. J. Homer, A. Ring, C. B. Wilen, A. Iwasaki, Mouse model of SARS-CoV-2 reveals inflammatory role of type I interferon signaling. *J. Exp. Med.* **217**, e20201241 (2020). doi:10.1084/jem.20201241 Medline
40. T. Mao, B. Israelow, C. Lucas, C. B. F. Vogels, M. L. Gomez-Calvo, O. Fedorova, M. I. Breban, B. L. Menasche, H. Dong, M. Linehan, C. B. Wilen, M. L. Landry, N. D. Grubaugh, A. M. Pyle, A. Iwasaki, K. Munyenyembe, I. M. Ott, M. E. Petrone, J. Rothman, A. E. Watkins, C. B. Wilen, M. L. Landry, N. D. Grubaugh, A. M. Pyle, A. Iwasaki, Yale SARS-CoV-2 Genome Surveillance Initiative, A stem-loop RNA RIG-I agonist protects against acute and chronic SARS-CoV-2 infection in mice. *J. Exp. Med.* **219**, e20211818 (2022). doi:10.1084/jem.20211818 Medline
41. D. C. Rio, M. Ares Jr., G. J. Hannon, T. W. Nilsen, Purification of RNA using TRIzol (TRI reagent). *Cold Spring Harb. Protoc.* **2010**, prot5439 (2010). doi:10.1101/pdb.prot5439 Medline
42. A. C. Kauffman, A. S. Piotrowski-Daspit, K. H. Nakazawa, Y. Jiang, A. Datye, W. M. Saltzman, Tunability of Biodegradable Poly(amine- co-ester) Polymers for Customized Nucleic Acid Delivery and Other Biomedical Applications. *Biomacromolecules* **19**, 3861–3873 (2018). doi:10.1021/acs.biomac.8b00997 Medline
43. Y. Jiang, Q. Lu, Y. Wang, E. Xu, A. Ho, P. Singh, Y. Wang, Z. Jiang, F. Yang, G. T. Tietjen, P. Cresswell, W. M. Saltzman, Quantitating Endosomal Escape of a Library of Polymers for mRNA Delivery. *Nano Lett.* **20**, 1117–1123 (2020). doi:10.1021/acs.nanolett.9b04426 Medline
44. F. Amanat, D. Stadlbauer, S. Strohmeier, T. H. O. Nguyen, V. Chromikova, M. McMahon, K. Jiang, G. A. Arunkumar, D. Jurczyszak, J. Polanco, M. Bermudez-Gonzalez, G. Kleiner, T. Aydillo, L. Miorin, D. S. Fierer, L. A. Lugo, E. M. Kojic, J. Stoever, S. T. H. Liu, C. Cunningham-Rundles, P. L. Felgner, T. Moran, A. García-Sastre, D. Caplivski, A. C. Cheng, K. Kedzierska, O. Vapalahti, J. M. Hepojoki, V. Simon, F. Krammer, A serological assay to detect SARS-CoV-2 seroconversion in humans. *Nat. Med.* **26**, 1033–1036 (2020). doi:10.1038/s41591-020-0913-5 Medline
45. M. Letko, A. Marzi, V. Munster, Functional assessment of cell entry and receptor usage for SARS-CoV-2 and other lineage B betacoronaviruses. *Nat. Microbiol.* **5**, 562–569 (2020). doi:10.1038/s41564-020-0688-y Medline

ACKNOWLEDGMENTS

We thank M. Linehan for her technical and logistical assistance. We thank B. Graham (NIH-VRC) for kindly providing VeroE6 cells overexpressing ACE2 and TMPRSS2. We thank the NIH tetramer Core Facility for providing PE-labeled SARS-CoV-2 S 539-546 tetramer (H-2K(b)) and APC-labeled SARS-CoV-2 S 62-76 MHC class II tetramer (I-A(b)). We thank H. Steach for providing the B cell tetramer production protocol. We thank C. Wilen and J. Wei for their technical expertise. We thank H.W. Suh for providing PACE polymers and A.S. Piotrowski-Daspit for her helpful discussions. We thank J. Frank for her technical assistance with

coronavirus spike sequence alignment. We also give special recognition of the services of B. Fontes and the Yale EH&S Department for their continuing assistance in safely conducting biosafety level 3 research. **Funding:** This work was in part supported by the Howard Hughes Medical Institute, the Fast Grant from Emergent Ventures at the Mercatus Center, and 1R01AI157488. A.I. is an Investigator of the Howard Hughes Medical Institute. B.I. is supported by NIAID T32AI007517 and K08AI163493. T.M. is supported by NIAID T32AI007019. W.M.S, A.S., and M.R. are supported by UG3 HL147352 from the NIH. **Author contributions:** B.I., T.M., and A.I. conceived and designed research with contributions from A.S., M.R., R.J.H., and W.M.S. T.M., B.I., A.S., M.R., L.Z., S.L., M.P.-H., R.J.H., and H.D. conducted experiments. T.M., B.I., and A.I. analyzed and interpreted results. B.I., T.M., and A.I. wrote the original manuscript which was revised and edited by all authors. **Competing interests:** A.I., T.M., and B.I. are listed as inventors on patent applications relating to intranasal spike-based SARS-CoV-2 vaccines filed by the Yale University. A.I., W.M.S., T.M, B.I., A.S., and M.H. are listed as inventors on patent applications relating to intranasal PACE nanoparticle delivery-based vaccines filed by the Yale University. W.M.S. and A.I. are cofounders of Xanadu Bio and T.M. and B.I are consultants for Xanadu Bio. **Data and materials availability:** All data needed to evaluate this paper's conclusions are present in the main text or supplemental materials. The reagents used to conduct experiments in this manuscript are either publicly available as described in Materials and Methods or are available with the appropriate material transfer agreement. PACE polymer is available from Mark Saltzman under a material transfer agreement with Yale University. **License information:** This work is licensed under a Creative Commons Attribution 4.0 International (CC BY 4.0) license, which permits unrestricted use, distribution, and reproduction in any medium, provided the original work is properly cited. To view a copy of this license, visit https://creativecommons.org/licenses/by/4.0/. This license does not apply to figures/photos/artwork or other content included in the article that is credited to a third party; obtain authorization from the rights holder before using such material.

SUPPLEMENTARY MATERIALS

science.org/doi/10.1126/science.abo2523
Figs. S1 to S10
Table S1
MDAR Reproducibility Checklist

Submitted 23 January 2022; resubmitted 22 July 2022
Accepted 24 October 2022
Published online 27 October 2022
10.1126/science.abo2523

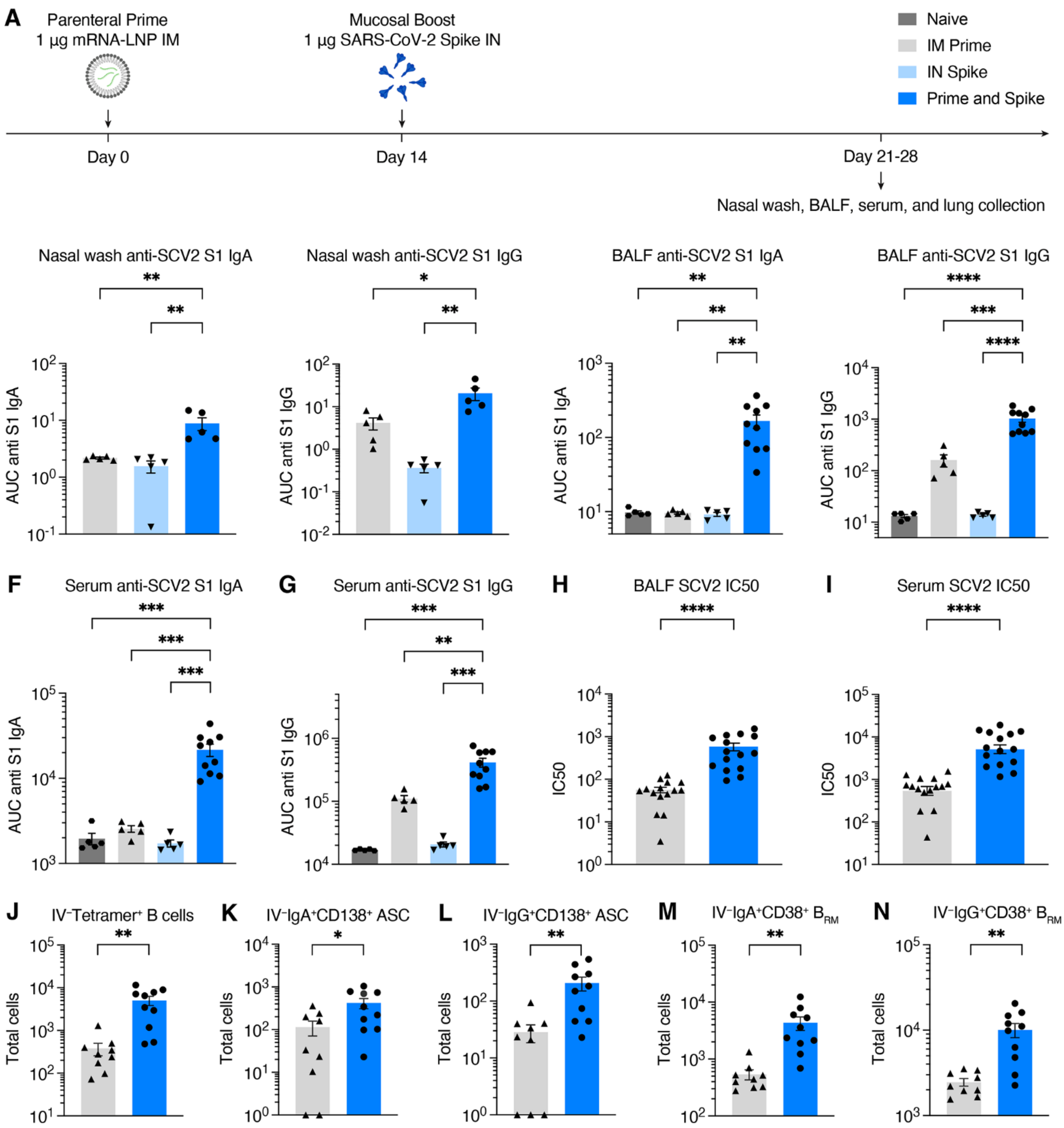

A
Parenteral Prime
1 µg mRNA-LNP IM
Mucosal Boost
1 µg SARS-CoV-2 Spike IN
Naive
IM Prime
IN Spike
Prime and Spike
Day 0
Day 14
Day 21-28
Nasal wash, BALF, serum, and lung collection
Nasal wash anti-SCV2 S1 IgA
Nasal wash anti-SCV2 S1 IgG
BALF anti-SCV2 S1 IgA
BALF anti-SCV2 S1 IgG
AUC anti S1 IgA
AUC anti S1 IgG
F
Serum anti-SCV2 S1 IgA
G
Serum anti-SCV2 S1 IgG
H
BALF SCV2 IC50
I
Serum SCV2 IC50
IC50
J
IV⁻Tetramer⁺ B cells
K
IV⁻IgA⁺CD138⁺ ASC
L
IV⁻IgG⁺CD138⁺ ASC
M
IV⁻IgA⁺CD38⁺ BRM
N
IV⁻IgG⁺CD38⁺ BRM
Total cells

Fig. 1. IN boosting with stabilized SARS-CoV-2 spike induces mucosal humoral memory. (**A**) Experimental schema: mice were intramuscularly (IM) immunized with 1 μg of mRNA-lipid nanoparticles (LNP) encoding full-length SARS-CoV-2 (SCV2) spike protein (Pfizer/BioNTech BNT162b2), followed by intranasal (IN) immunization with 1 μg of prefusion-stabilized (Hexapro), trimeric, recombinant SCV2 spike protein 14 days following mRNA-LNP immunization. Fourteen days post IN boost, serum, bronchoalveolar lavage fluids (BALF), and nasal washes were collected to assess binding and neutralizing antibody responses. Lung tissues were collected for extravascular B cell analysis. (**B** to **G**) Measurement of SCV2 spike S1 subunit-specific nasal wash IgA (B), nasal wash IgG (C), BALF IgA (D), BALF IgG (E), serum IgA (F), and serum IgG (G) in naïve mice, mice immunized with mRNA-LNP IM (IM Prime), mice immunized with the spike protein IN (IN Spike), or mice IM primed and IN boosted with spike (P&S). (**H** to **K**) Measurement of neutralization titer against SCV2 spike-pseudotyped vesicular stomatitis virus (VSV) in BALF (H) and serum (I). (**J** to **N**) Using CD45 intravenous (IV) labeling, various extravascular (IV labeling antibody negative) B cell subsets were measured, including RBD tetramer-binding B cells, IgA^+ resident memory B (B_{RM}) cells, IgG^+ B_{RM} cells, IgA^+ antibody-secreting cells (ASC), and IgG^+ ASC in lung tissues from IM Prime or P&S mice. Mean ± s.e.m. Statistical significance was calculated by [(B) to (G)] one-way ANOVA or [(H) to (N)] Student's *t* test; $*P \leq 0.05$, $**P \leq 0.01$, $***P \leq 0.001$, $****P \leq 0.0001$. Individual data points are represented and are pooled from two or three independent experiments.

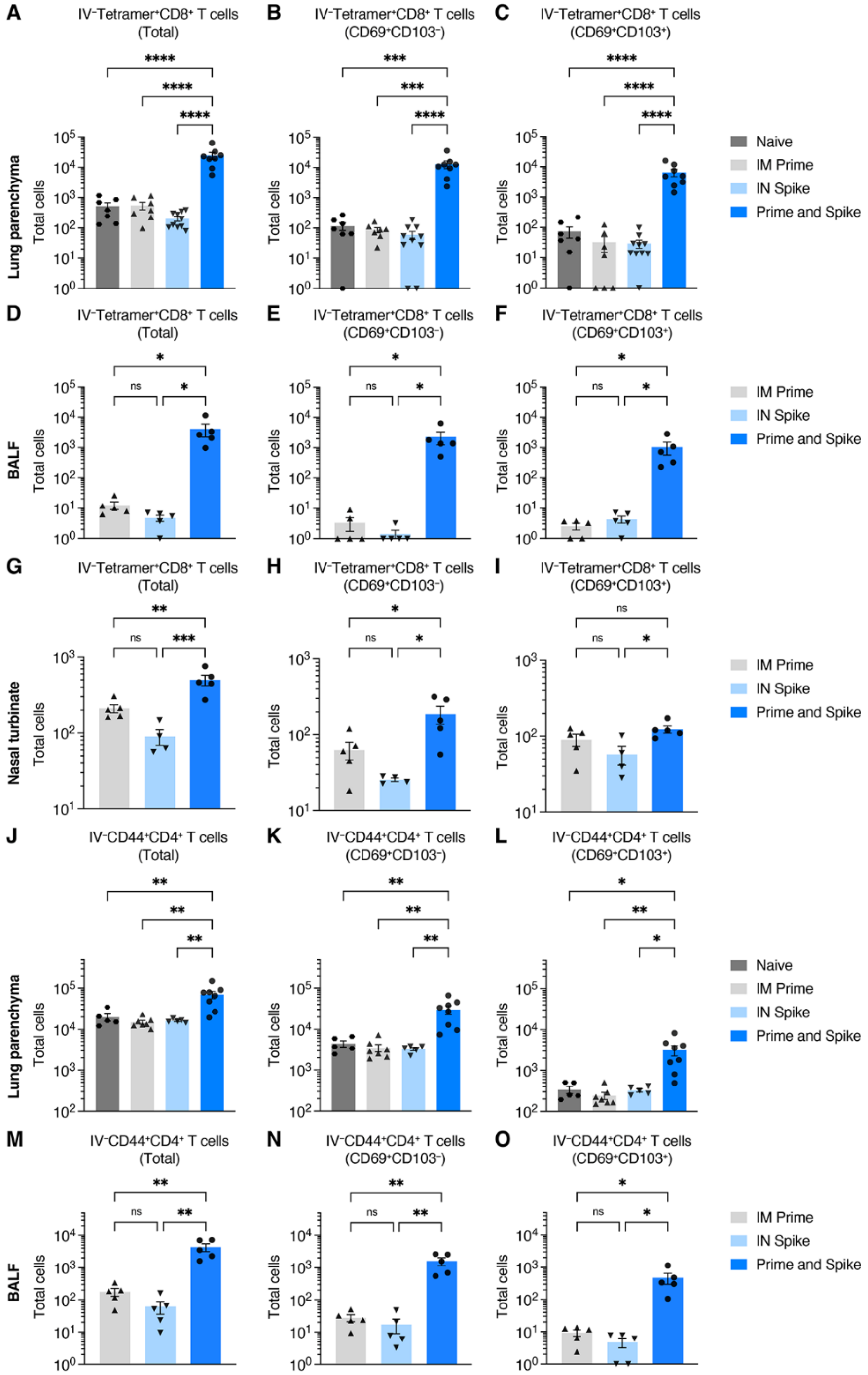

Fig. 2. IN boosting with stabilized SARS-CoV-2 spike induces mucosal T cell memory. K18-hACE2 mice were IM primed with 1 μg mRNA-LNP and 14 days later IN boosted with 1 μg SCV2 spike. Lung tissues, BALF, and nasal turbinates were collected for extravascular T cell analysis. Lung tissues were collected 14 days post boost, whereas BALF and nasal turbinates were obtained 7 days post boost. (**A** to **I**) Extravascular CD8 T cell responses: quantification of SCV2 spike-specific tetramer$^+$ CD8 T cells, CD69$^+$CD103$^-$tetramer$^+$ CD8 T cells, or CD69$^+$CD103$^+$tetramer$^+$ CD8 T cells in [(A) to (C)] lung tissues, [(D) to (F)] BALF, or [(G) to (I)] nasal turbinates from naïve, IM Prime, IN Spike, or P&S mice. (**J** to **O**) Extravascular CD4 T cell responses: quantification of activated polyclonal CD4 T cells, CD69$^+$CD103$^-$ CD4 T cells, or CD69$^+$CD103$^+$ CD4 T cells in [(J) to (L)] lung tissues or [(M) to (O)] BALF from naïve, IM Prime, IN Spike, or P&S mice. Mean ± s.e.m. Statistical significance was calculated by one-way ANOVA followed by Tukey' correction [(B) to (O)]; *$P \leq 0.05$, **$P \leq 0.01$, ***$P \leq 0.001$, ****$P \leq 0.0001$. Individual data points are represented and are pooled from two or three independent experiments.

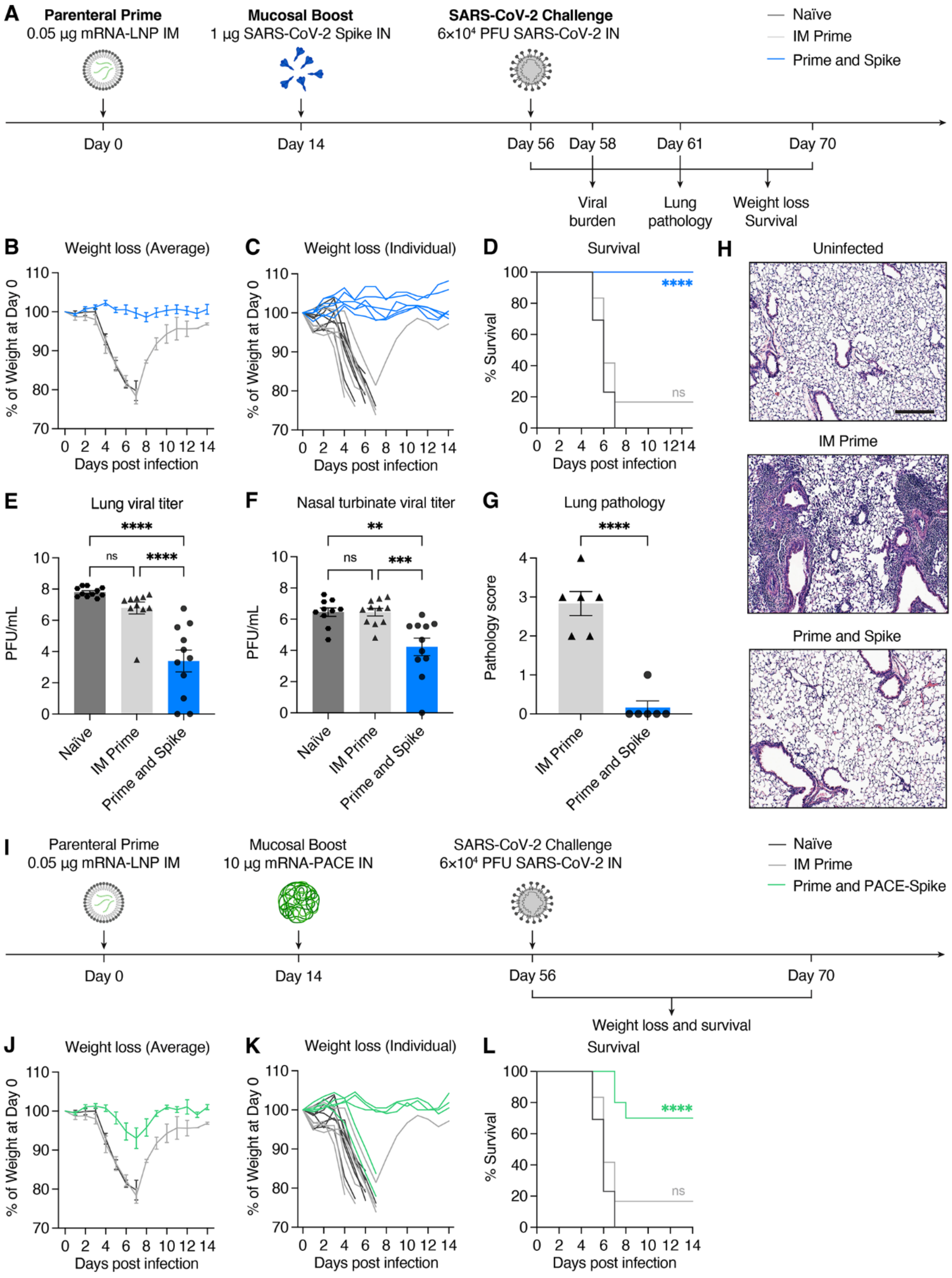
A
Parenteral Prime
0.05 µg mRNA-LNP IM
Mucosal Boost
1 µg SARS-CoV-2 Spike IN
SARS-CoV-2 Challenge
6×10⁴ PFU SARS-CoV-2 IN
Naïve
IM Prime
Prime and Spike
Day 0
Day 14
Day 56
Day 58
Day 61
Day 70
Viral burden
Lung pathology
Weight loss Survival
B
Weight loss (Average)
% of Weight at Day 0
Days post infection
C
Weight loss (Individual)
% of Weight at Day 0
Days post infection
D
Survival
% Survival
****
ns
Days post infection
H
Uninfected
IM Prime
Prime and Spike
E
Lung viral titer
PFU/mL
****
ns
****
Naïve
IM Prime
Prime and Spike
F
Nasal turbinate viral titer
PFU/mL
**
ns
***
Naïve
IM Prime
Prime and Spike
G
Lung pathology
Pathology score
****
IM Prime
Prime and Spike
I
Parenteral Prime
0.05 µg mRNA-LNP IM
Mucosal Boost
10 µg mRNA-PACE IN
SARS-CoV-2 Challenge
6×10⁴ PFU SARS-CoV-2 IN
Naïve
IM Prime
Prime and PACE-Spike
Day 0
Day 14
Day 56
Day 70
Weight loss and survival
J
Weight loss (Average)
% of Weight at Day 0
Days post infection
K
Weight loss (Individual)
% of Weight at Day 0
Days post infection
L
Survival
% Survival
****
ns
Days post infection

Fig. 3. Intranasal SARS-CoV-2 spike boosting protects against COVID-19-like disease. (**A**) Experimental schema: K18-hACE2 mice were IM primed with 0.05 µg of mRNA-LNP and IN boosted with 1 µg of spike IN 14 days post IM Prime. Six weeks post boost, mice were challenged with 6×10^4 PFU SCV2 (2019n-CoV/USA_WA1/2020). The first cohort was used to evaluate weight loss and survival up to 14 days post infection (DPI). The second cohort was used to collect lung and nasal turbinate tissues 2 DPI for viral titer measurement. The third cohort was used to collect lung tissues 5 DPI for histological assessment. (**B** to **D**) Weight loss and survival of naïve, IM Prime, or P&S mice from 1 to 14 DPI. (**E** to **F**) Measurement of infectious virus titer in lung and nasal turbinate tissues at 2 DPI by plaque assay. (**G**) Pathology score of lung sections at 5 DPI by hematoxylin and eosin (H&E) staining. (**H**) Representative H&E staining results from uninfected, IM Prime, or P&S mice. Scale bar: 250 µm. Sections are representative of multiple sections from at least five mice per group. (**I**) Experimental schema: K18-hACE2 mice were IM primed with 0.05 µg of mRNA-LNP and IN boosted with 10 µg of mRNA encapsulated by PACE (IN PACE-Spike) 14 days post IM Prime. Six weeks post boost, mice were challenged with 6×10^4 PFU SCV2 (2019n-CoV/USA_WA1/2020). Weight loss and survival were monitored up to 14 DPI. (**J** to **L**) Weight loss and survival of naïve, IM Prime, or Prime and PACE-Spike K18-hACE2 mice from 1 to 14 DPI. Mean ± s.e.m.; Statistical significance was calculated by [(D) and (L)] log-rank Mantel–Cox test, [(E) and (F)] one-way ANOVA followed by Tukey's correction, or (G) Student's *t* test; *$P \le 0.05$, **$P \le 0.01$, ***$P \le 0.001$, ****$P \le 0.0001$. Individual data points are represented and are pooled from two independent experiments.

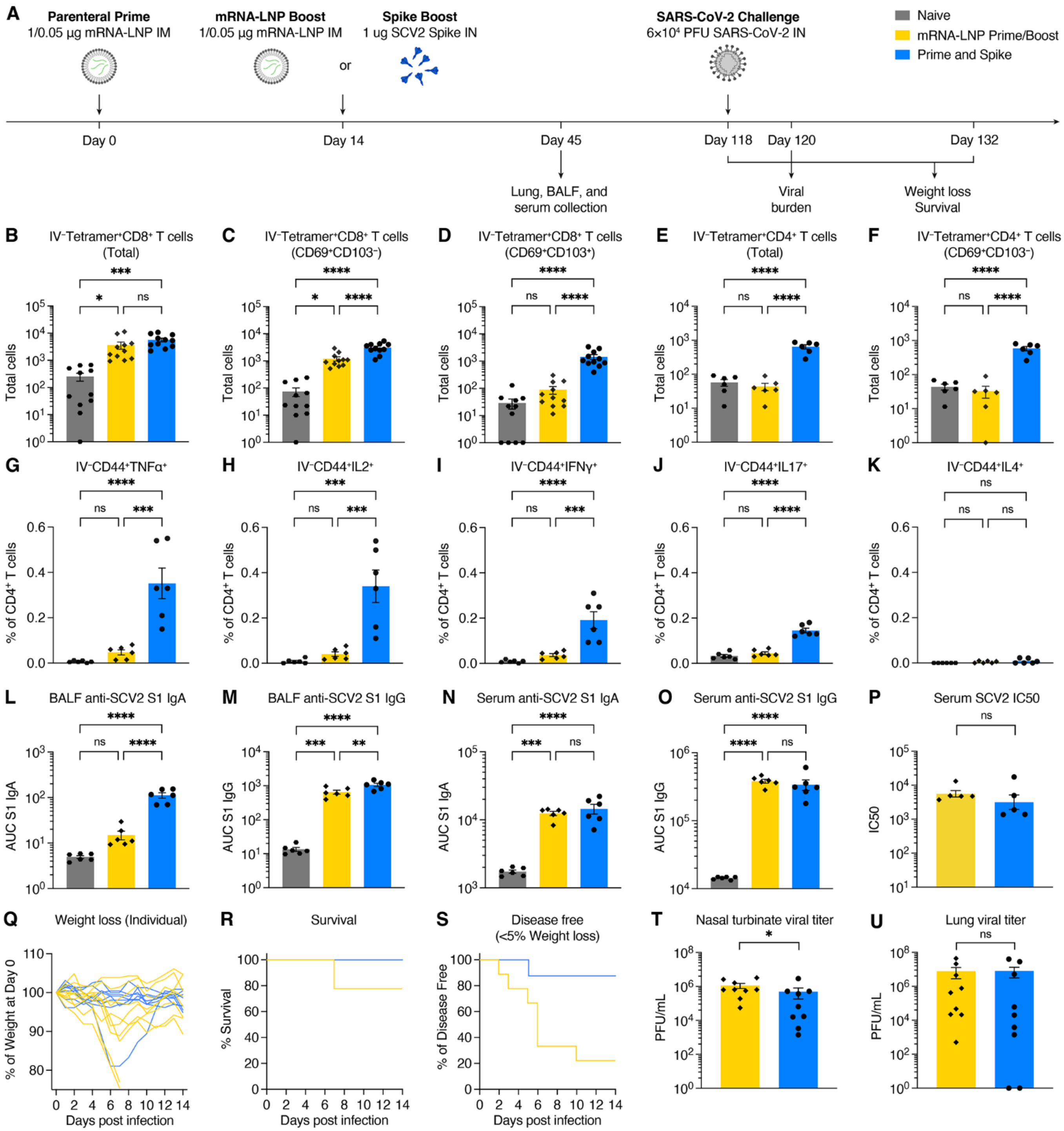

A
Parenteral Prime
1/0.05 µg mRNA-LNP IM
mRNA-LNP Boost
1/0.05 µg mRNA-LNP IM
or
Spike Boost
1 ug SCV2 Spike IN
SARS-CoV-2 Challenge
6×10⁴ PFU SARS-CoV-2 IN
Naive
mRNA-LNP Prime/Boost
Prime and Spike
Day 0
Day 14
Day 45
Day 118
Day 120
Day 132
Lung, BALF, and serum collection
Viral burden
Weight loss Survival
B IV⁻Tetramer⁺CD8⁺ T cells (Total)
C IV⁻Tetramer⁺CD8⁺ T cells (CD69⁺CD103⁻)
D IV⁻Tetramer⁺CD8⁺ T cells (CD69⁺CD103⁺)
E IV⁻Tetramer⁺CD4⁺ T cells (Total)
F IV⁻Tetramer⁺CD4⁺ T cells (CD69⁺CD103⁻)
Total cells
G IV⁻CD44⁺TNFα⁺
H IV⁻CD44⁺IL2⁺
I IV⁻CD44⁺IFNγ⁺
J IV⁻CD44⁺IL17⁺
K IV⁻CD44⁺IL4⁺
% of CD4⁺ T cells
L BALF anti-SCV2 S1 IgA
M BALF anti-SCV2 S1 IgG
N Serum anti-SCV2 S1 IgA
O Serum anti-SCV2 S1 IgG
P Serum SCV2 IC50
AUC S1 IgA
AUC S1 IgG
IC50
Q Weight loss (Individual)
% of Weight at Day 0
R Survival
% Survival
S Disease free (<5% Weight loss)
% of Disease Free
Days post infection
T Nasal turbinate viral titer
U Lung viral titer
PFU/mL

Fig. 4. IN spike boosting elicits enhanced mucosal immunity with similar systemic humoral responses to IM mRNA-LNP boosting. (**A**) Experimental schema: K18-hACE2 mice were IM primed with 1 μg of mRNA-LNP, followed 14 days later by boosting with 1 μg of mRNA-LNP IM or 1 μg of SCV2 spike IN. Forty-five days post prime, lung tissues were collected for T cell analysis by flow cytometry, and BALF and serum were collected for antibody measurement. K18-hACE2 mice were IM primed with 0.05 μg of mRNA-LNP, followed 14 days later by boosting with 0.05 μg of mRNA-LNP IM, or 1 μg of SCV2 spike IN and challenged with $6{\times}10^4$ PFU SCV2 at 118 days post prime. (**B** to **D**) Quantification of total tetramer$^+$ CD8 T cells, CD69$^+$CD103$^-$tetramer$^+$ CD8 T cells, or CD69$^+$CD103$^+$tetramer$^+$ CD8 T cells in lung tissues from naïve, mRNA-LNP prime–boost, or P&S mice. (**E** to **F**) Quantification of total tetramer$^+$ CD4 T cells or CD69$^+$CD103$^-$tetramer$^+$ CD4 T cells in lung tissues. (**G** to **K**) Lung lymphocytes were isolated via Percoll gradient and restimulated with spike peptide megapool from SCV2. Intracellular cytokine staining was performed to assess antigen-specific production of TNF-α, IL-2, IFN-γ, IL-17, and IL-4 by extravascular IV-CD45$^-$CD44$^+$ CD4 T cells. (**L** to **O**) Measurement of SCV2 spike S1 subunit-specific BALF IgA (L), BALF IgG (M), serum IgA (N), and serum IgG (O) in naïve, mRNA-LNP prime–boost, or P&S mice. (**P**) Measurement of neutralization titers against SCV2 spike-pseudotyped VSV. (**Q** to **S**) Weight loss, survival, and disease-free survival (<5% maximum weight loss) of mRNA-LNP prime–boost or P&S mice from 1 to 14 DPI. (**T** and **U**) Measurement of infectious virus titer in lung and nasal turbinate tissues at 2 DPI by plaque assay. To reduce overall number of experimental animals used, control data points from naïve and mRNA prime–boost are common to Figs. 4 and 6. Mean ± s.e.m.; Statistical significance was calculated by [(B) to (O)] one-way ANOVA followed by Tukey correction, [(P), (T), and (U)] or Student's *t* test, and by log-rank Mantel–Cox test (R,S); $*P \le 0.05$, $**P \le 0.01$, $***P \le 0.001$, $****P \le 0.0001$. Individual data points are represented and are pooled from two independent experiments.

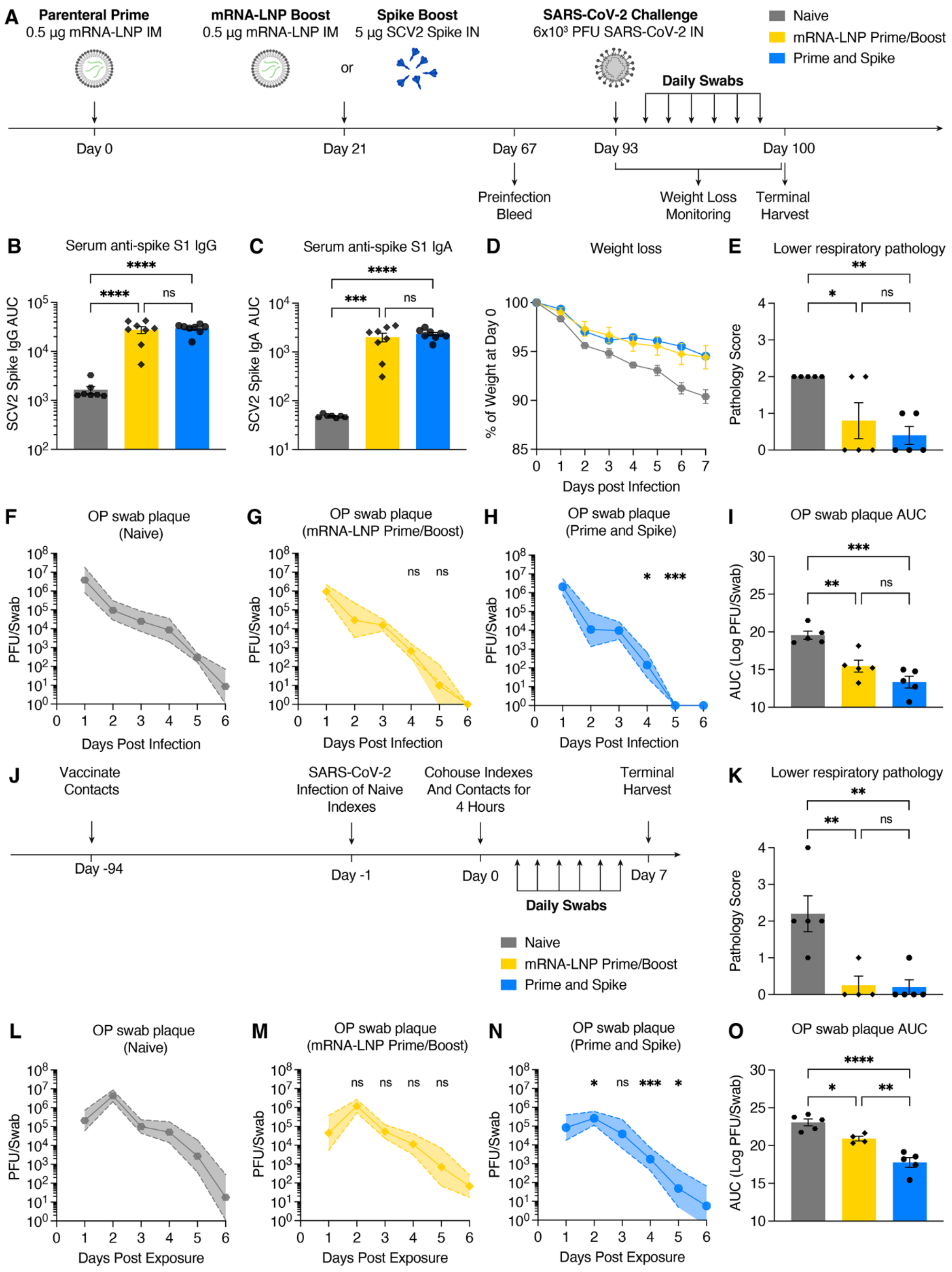

A
Parenteral Prime
0.5 µg mRNA-LNP IM
mRNA-LNP Boost
0.5 µg mRNA-LNP IM
or
Spike Boost
5 µg SCV2 Spike IN
SARS-CoV-2 Challenge
6x10³ PFU SARS-CoV-2 IN
Naive
mRNA-LNP Prime/Boost
Prime and Spike
Daily Swabs
Day 0
Day 21
Day 67
Day 93
Day 100
Preinfection Bleed
Weight Loss Monitoring
Terminal Harvest
B
Serum anti-spike S1 IgG
SCV2 Spike IgG AUC
C
Serum anti-spike S1 IgA
SCV2 Spike IgA AUC
D
Weight loss
% of Weight at Day 0
Days post Infection
E
Lower respiratory pathology
Pathology Score
F
OP swab plaque (Naive)
G
OP swab plaque (mRNA-LNP Prime/Boost)
H
OP swab plaque (Prime and Spike)
PFU/Swab
Days Post Infection
I
OP swab plaque AUC
AUC (Log PFU/Swab)
J
Vaccinate Contacts
SARS-CoV-2 Infection of Naive Indexes
Cohouse Indexes And Contacts for 4 Hours
Terminal Harvest
Day -94
Day -1
Day 0
Day 7
Daily Swabs
Naive
mRNA-LNP Prime/Boost
Prime and Spike
K
Lower respiratory pathology
Pathology Score
L
OP swab plaque (Naive)
M
OP swab plaque (mRNA-LNP Prime/Boost)
N
OP swab plaque (Prime and Spike)
PFU/Swab
Days Post Exposure
O
OP swab plaque AUC
AUC (Log PFU/Swab)

Fig. 5. IN spike boosting leads to reduced viral transmission in hamster model. (**A**) Experimental schema: Syrian hamsters were IM primed with 0.5 μg of mRNA-LNP, followed 21 days later by boosting with 0.5 μg of mRNA-LNP IM or 5 μg of SCV2 spike IN. (**B** and **C**) 67 days post prime serum IgG and IgA were assessed by ELISA. At 93 days post prime Naïve, mRNA-LNP prime–boost, and P&S hamsters were infected with 6×10^3 PFU SCV2. (**D**) Weight loss as percent of starting. (**E**) Histopathologic analysis of lung samples at 7 DPI. (**F** to **H**) Viral titer from oropharyngeal swabs are shown as mean (symbols) and standard deviation (shade), *p*-value relative to control at the same time point. (**I**) Area under the curve analysis (AUC) for viral titer over 6 DPI. (**J**) Transmission experimental schema: Syrian hamsters vaccinated as above were co-housed for 4 hours with naïve donor hamsters who had been infected 24 hours earlier with 6×10^3 PFU SCV2. (**K**) Histopathologic analysis of lung samples at 7 days post exposure. (**L** to **N**) Viral titer from oropharyngeal swabs are shown as mean (symbols) and standard deviation (shade), *P*-value relative to control at the same time point. (**O**) Area under the curve analysis (AUC) for viral titer over 6 DPI. Mean ± s.e.m.; Statistical significance was calculated by [(B), (C), (E), (I), (K), and (O)] one-way ANOVA followed by Tukey's correction, [(F) to (H)] Mixed-effect analysis followed by Tukey's multiple comparison test, or [(L) to (N)] two-way ANOVA followed by Dunnett's multiple comparisons test; $*P \leq 0.05$, $**P \leq 0.01$, $***P \leq 0.001$, $****P \leq 0.0001$. Individual data points are represented from one independent experiment.

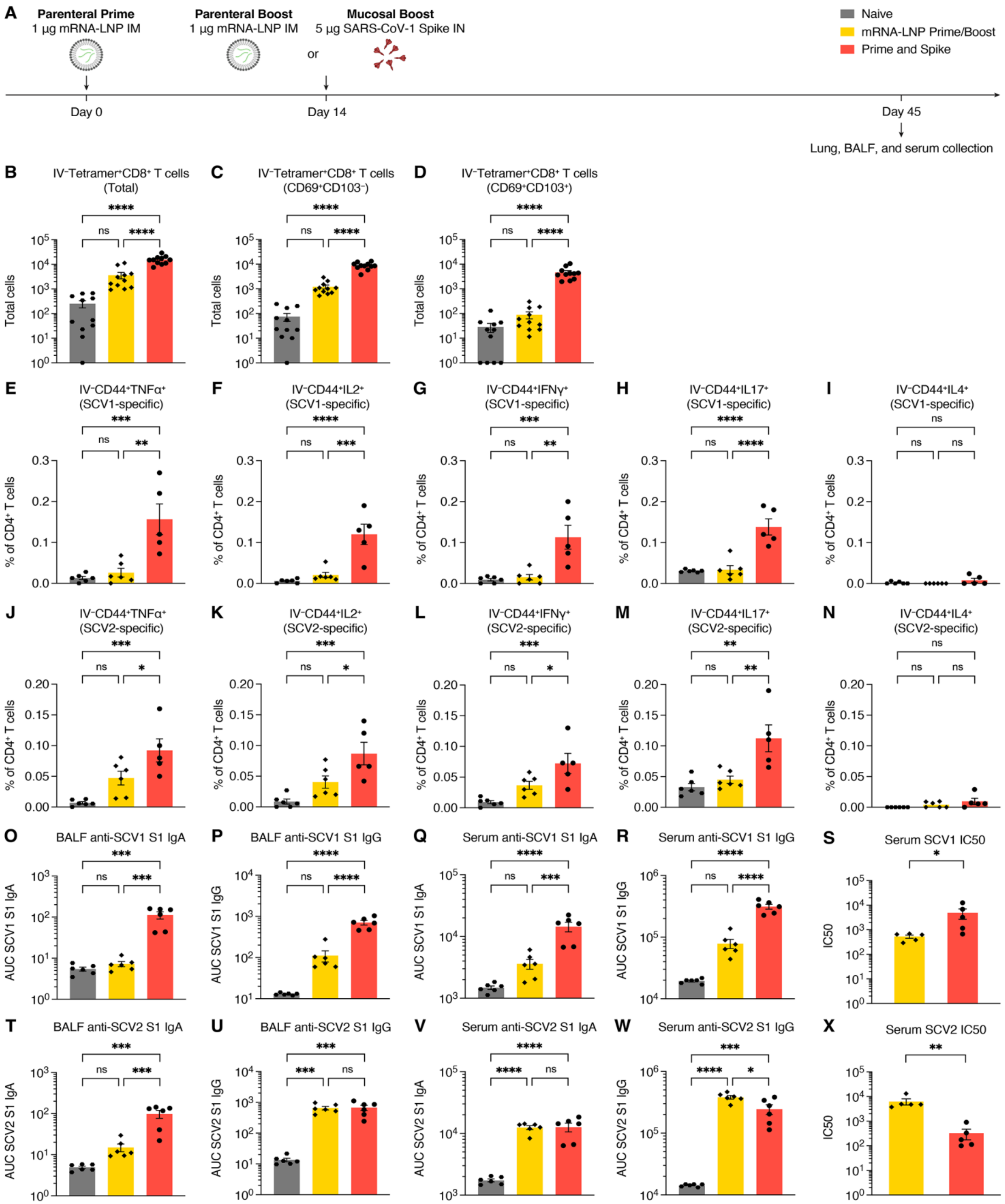
A
Parenteral Prime
1 µg mRNA-LNP IM
Parenteral Boost
1 µg mRNA-LNP IM
or
Mucosal Boost
5 µg SARS-CoV-1 Spike IN
Day 0
Day 14
Day 45
Lung, BALF, and serum collection
Naive
mRNA-LNP Prime/Boost
Prime and Spike
B
IV⁻Tetramer⁺CD8⁺ T cells (Total)
C
IV⁻Tetramer⁺CD8⁺ T cells (CD69⁺CD103⁻)
D
IV⁻Tetramer⁺CD8⁺ T cells (CD69⁺CD103⁺)
Total cells
E
IV⁻CD44⁺TNFα⁺ (SCV1-specific)
F
IV⁻CD44⁺IL2⁺ (SCV1-specific)
G
IV⁻CD44⁺IFNγ⁺ (SCV1-specific)
H
IV⁻CD44⁺IL17⁺ (SCV1-specific)
I
IV⁻CD44⁺IL4⁺ (SCV1-specific)
J
IV⁻CD44⁺TNFα⁺ (SCV2-specific)
K
IV⁻CD44⁺IL2⁺ (SCV2-specific)
L
IV⁻CD44⁺IFNγ⁺ (SCV2-specific)
M
IV⁻CD44⁺IL17⁺ (SCV2-specific)
N
IV⁻CD44⁺IL4⁺ (SCV2-specific)
% of CD4⁺ T cells
O
BALF anti-SCV1 S1 IgA
P
BALF anti-SCV1 S1 IgG
Q
Serum anti-SCV1 S1 IgA
R
Serum anti-SCV1 S1 IgG
S
Serum SCV1 IC50
AUC SCV1 S1 IgA
AUC SCV1 S1 IgG
IC50
T
BALF anti-SCV2 S1 IgA
U
BALF anti-SCV2 S1 IgG
V
Serum anti-SCV2 S1 IgA
W
Serum anti-SCV2 S1 IgG
X
Serum SCV2 IC50
AUC SCV2 S1 IgA
AUC SCV2 S1 IgG

Fig. 6. Heterologous IN boosting with SARS-CoV-1 spike enhances pre-existing SCV2-specific immunity and broadens reactivities to SCV1. (**A**) Experimental schema: K18-hACE2 mice were IM primed with 1 μg of mRNA-LNP, followed by boosting with 1 μg of mRNA-LNP IM, or 5 μg of prefusion-stabilized, trimeric, recombinant SARS-CoV-1 (SCV1) spike IN (IN SpikeX) 14 days post prime. (**B** to **D**) Quantification of total tetramer$^+$ CD8 T cells, CD69$^+$CD103$^-$tetramer$^+$ CD8 T cells, or CD69$^+$CD103$^+$tetramer$^+$ CD8 T cells in lung tissues from naïve, mRNA-LNP prime–boost, or P&Sx mice. (**E** to **N**) Precool gradient purified lung lymphocytes were re-stimulated with spike peptide megapool from [(E) to (I)] SCV1 or [(J) to (N)] SCV2 and intracellular cytokine staining was performed to assess antigen-specific production of TNF-α, IL-2, IFN-γ, IL-17, and IL-4 by extravascular IV-CD45$^-$CD44$^+$ CD4 T cells expressing. (**O** to **S**) Measurement of SCV1 spike S1 subunit-specific BALF IgA and IgG, and serum IgA and IgG. (**S**) Measurement of neutralization titer against SCV1 spike-pseudotyped VSV. (**T** to **W**) Measurement of SCV2 spike S1 subunit-specific BALF IgA and IgG, and serum IgA and IgG. (**X**) Measurement of neutralization titer against SCV2 spike-pseudotyped VSV. To reduce overall number of experimental animals used, control data points from naïve and mRNA prime–boost are common to Figs. 4 and 6. Mean ± s.e.m.; statistical significance was calculated by one-way ANOVA followed by Tukey's correction except for [(S) and (X)] Student's *t* test; $*P \leq 0.05$, $**P \leq 0.01$, $***P \leq 0.001$, $****P \leq 0.0001$. Individual data points are represented and are pooled from two independent experiments.
