# Supplementary material for: Unadjuvanted intranasal spike vaccine elicits protective mucosal immunity against sarbecoviruses
Source: Science. 2022 Nov 25;378(6622):eabo2523. doi: 10.1126/science.abo2523 (PMC9798903; doi:10.1126/science.abo2523)
Supplement: Supplementary file 2 — Figs. S1 to S10 Table S1 [file science.abo2523_sm.pdf]

Science AAAS

Supplementary Materials for

Unadjuvanted intranasal spike vaccine elicits protective mucosal immunity against sarbecoviruses

Tianyang Mao *et al*.

Corresponding authors: Benjamin Israelow, benjamin.goldman-israelow@yale.edu; Akiko Iwasaki, akiko.iwasaki@yale.edu

Science **378**, eabo2523 (2022)
DOI: 10.1126/science.abo2523

The PDF file includes:

Figs. S1 to S10
Table S1

Other Supplementary Material for this manuscript includes the following:

MDAR Reproducibility Checklist

Figure S1

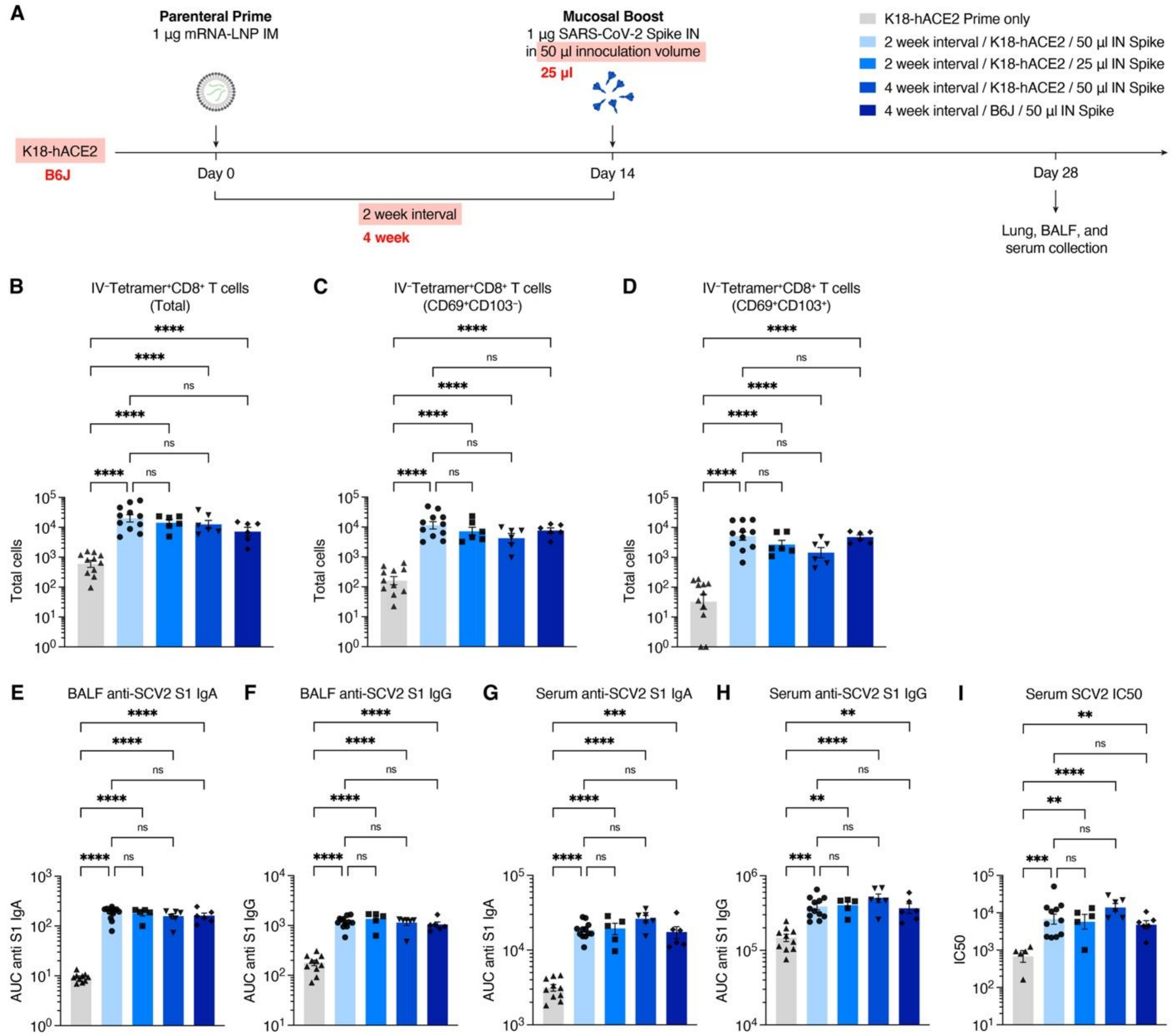

Fig S1. IN spike boost mediated mucosal immunity is not affected by host genotype, boosting interval or intranasal volume. (**A**) Experimental schema: K18-hACE2 or C57BL/6J (B6J) mice were IM primed with 1 µg of mRNA-LNP and IN boosted with 1 µg of SCV2 spike 2 or 4 weeks post IM Prime in 25 or 50 µl of inoculation volume. Fourteen days post boost, lung tissues were collected for T cell analysis by flow cytometry and BALF and blood were collected for antibody measurement. (**B to D**) Quantification of total tetramer$^+$ CD8 T cells, CD69$^+$CD103$^-$tetramer$^+$ CD8 T cells, or CD69$^+$CD103$^+$tetramer$^+$ CD8 T cells in lung tissues. (**E to H**) Measurement of SCV2 spike S1 subunit-specific BALF IgA (**E**), BALF IgG (**F**), serum IgA (**G**), and serum IgG (**H**). Measurement of neutralization titers against SCV2 spike-pseudotyped VSV (**I**) Mean ± s.e.m.; Statistical significance was calculated one-way ANOVA followed by Tukey's correction (**B to H**); *P≤0.05, **P≤0.01, ***P≤0.001, ****P≤0.0001. Individual data points are represented and are pooled from two independent experiments.

Figure S2

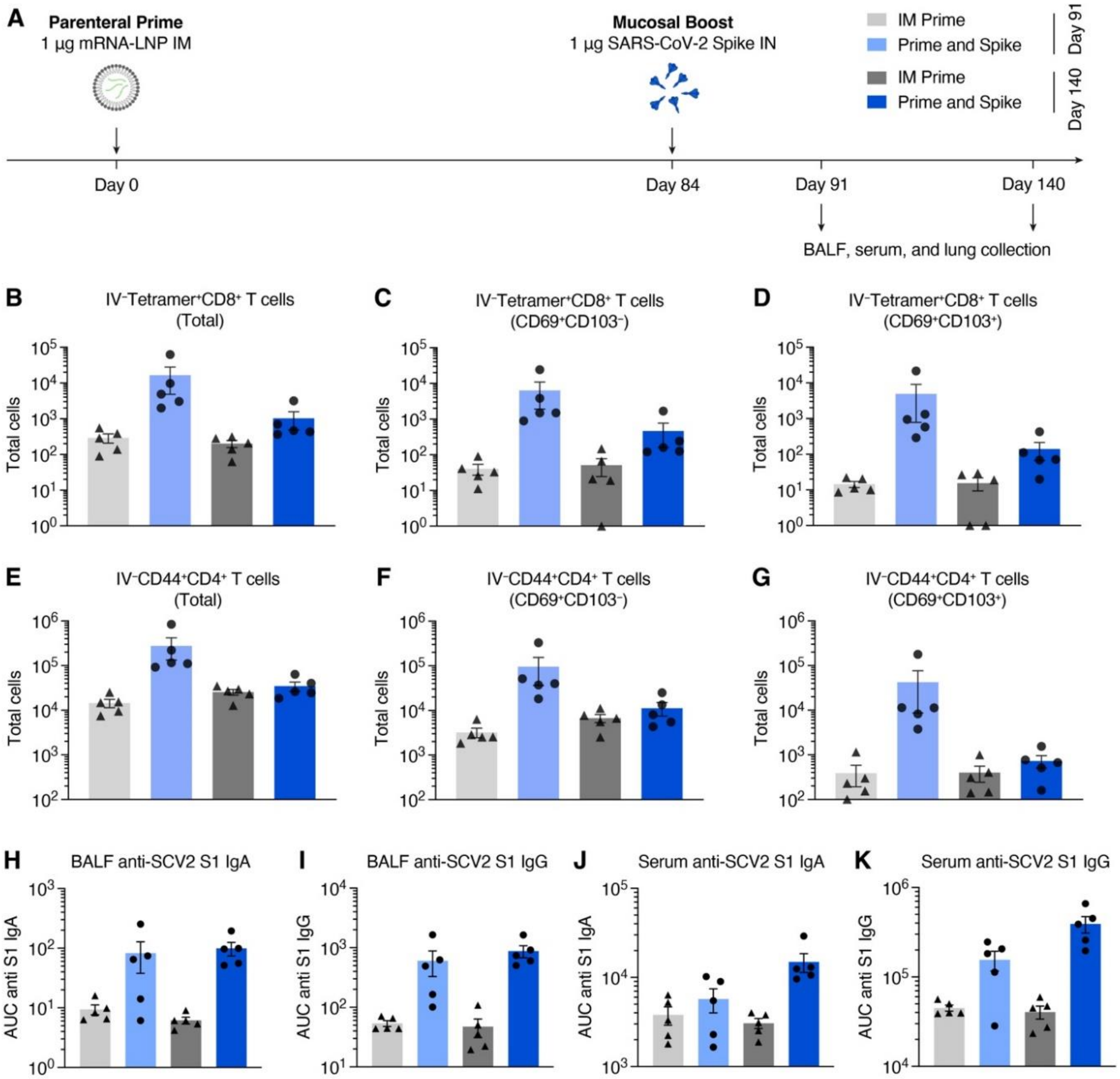

Fig S2. Delayed boosting with IN spike induces durable mucosal immunity. (**A**) Experimental schema: K18-hACE2 mice were IM primed with 1 μg of mRNA-LNP and IN boosted with 1 μg of SCV2 spike 12 weeks post IM Prime. Lung tissues were collected for T cell analysis by flow cytometry and BALF and blood were collected for antibody measurement 7 and 56 days post boost. (**B to D**) Quantification of total tetramer^{+} CD8 T cells, CD69^{+}CD103^{-}tetramer^{+} CD8 T cells, or CD69^{+}CD103^{+}tetramer^{+} CD8 T cells in lung tissues from IM Prime or P&S mice 7 and 56 days post boost. (**E to G**) Quantification of total activated, polyclonal CD4 T cells, CD69^{+}CD103^{-} CD4 T cells, or CD69^{+}CD103^{+} CD4 T cells in lung tissues from IM Prime or P&S mice 7 and 56 days post boost. (**H to K**) Measurement of SCV2 spike S1 subunit-specific BALF IgA (**H**), BALF IgG (**I**), serum IgA (**J**), and serum IgG (**K**) in IM Prime or P&S mice 7 and 56 days post boost. Mean ± s.e.m.; Statistical significance was calculated two-way ANOVA followed by Tukey's correction (**H to K**); *$P \leq 0.05$, **$P \leq 0.01$, ***$P \leq 0.001$, ****$P \leq 0.0001$. Individual data points are represented and are pooled from two independent experiments.

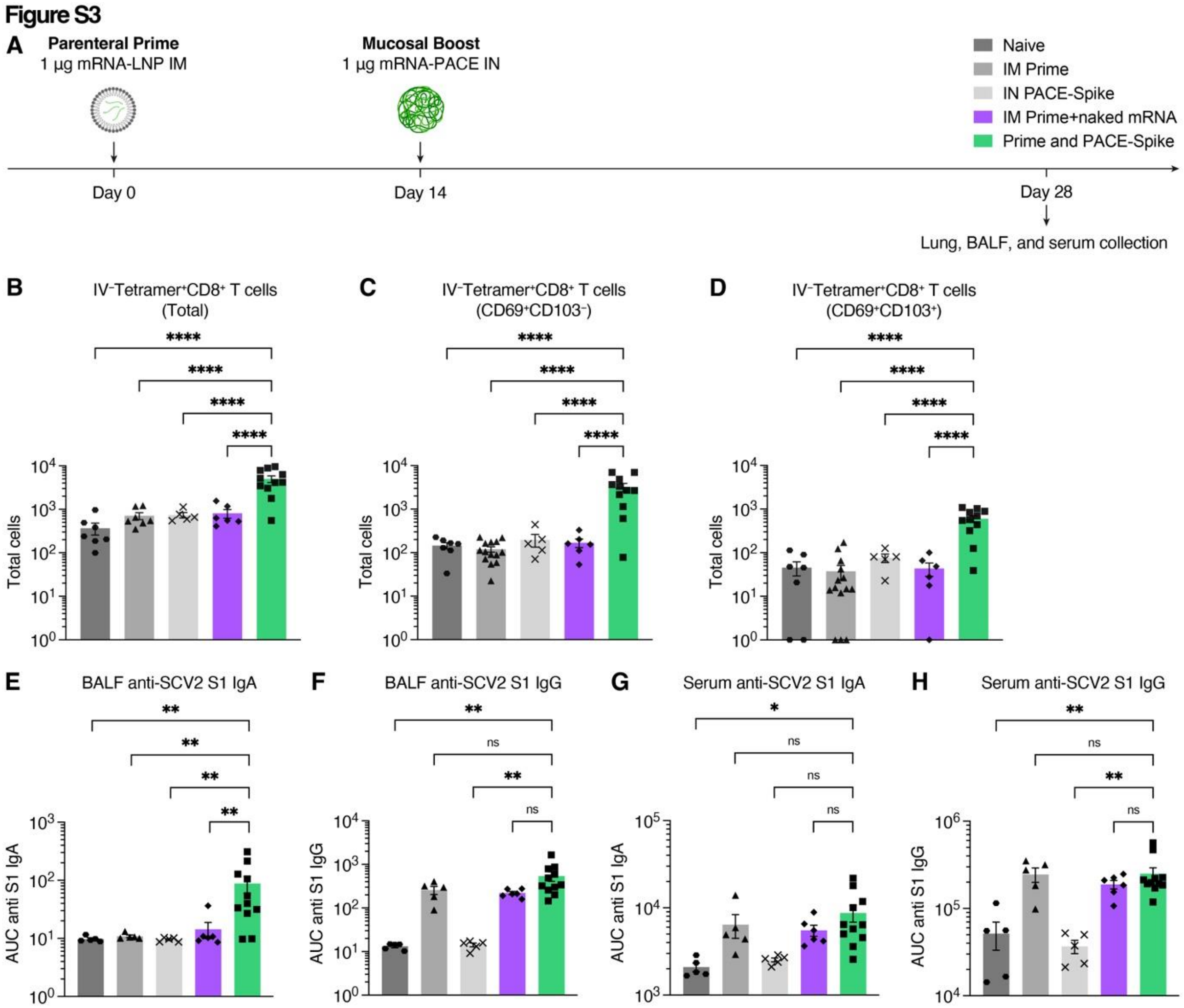

Fig S3. IN delivery of SCV2 spike mRNA encapsulated in poly(amine-co-ester) (PACE) terpolymers mediates mucosal boosting. (**A**) Experimental schema: K18-hACE2 mice were IM primed with 1 µg of mRNA-LNP, followed by IN boosting with 1 µg of naked mRNA (IN naked mRNA) or 1 µg of mRNA encapsulated by PACE (IN PACE-Spike) 14 days post IM Prime. Fourteen days post IN boost, BALF and blood were collected for antibody measurement. Lung tissues were collected for CD8 T cell analysis. (**B to D**) Quantification of total tetramer$^+$ CD8 T cells, CD69$^+$CD103$^-$tetramer$^+$ CD8 T cells, or CD69$^+$CD103$^+$tetramer$^+$ CD8 T cells in lung tissues from naïve, IM Prime, IN PACE-Spike, IM Prime+IN naked mRNA, or Prime and PACE-Spike mice. (**E to H**) Measurement of SARS-CoV-2 spike S1 subunit-specific BALF IgA (**E**), BALF IgG (**F**), serum IgA (**G**), and serum IgG (**H**) in naïve, IM Prime, IN PACE-Spike, IM Prime+IN naked mRNA, or Prime and PACE-Spike mice. Mean ± s.e.m.; Statistical significance was calculated by one-way ANOVA followed by Tukey's correction (**B to D**) or two-way ANOVA followed by Tukey's correction (**E to H**); *$P\leq0.05$, **$P\leq0.01$, ***$P\leq0.001$, ****$P\leq0.0001$. Data are pooled from two independent experiments.

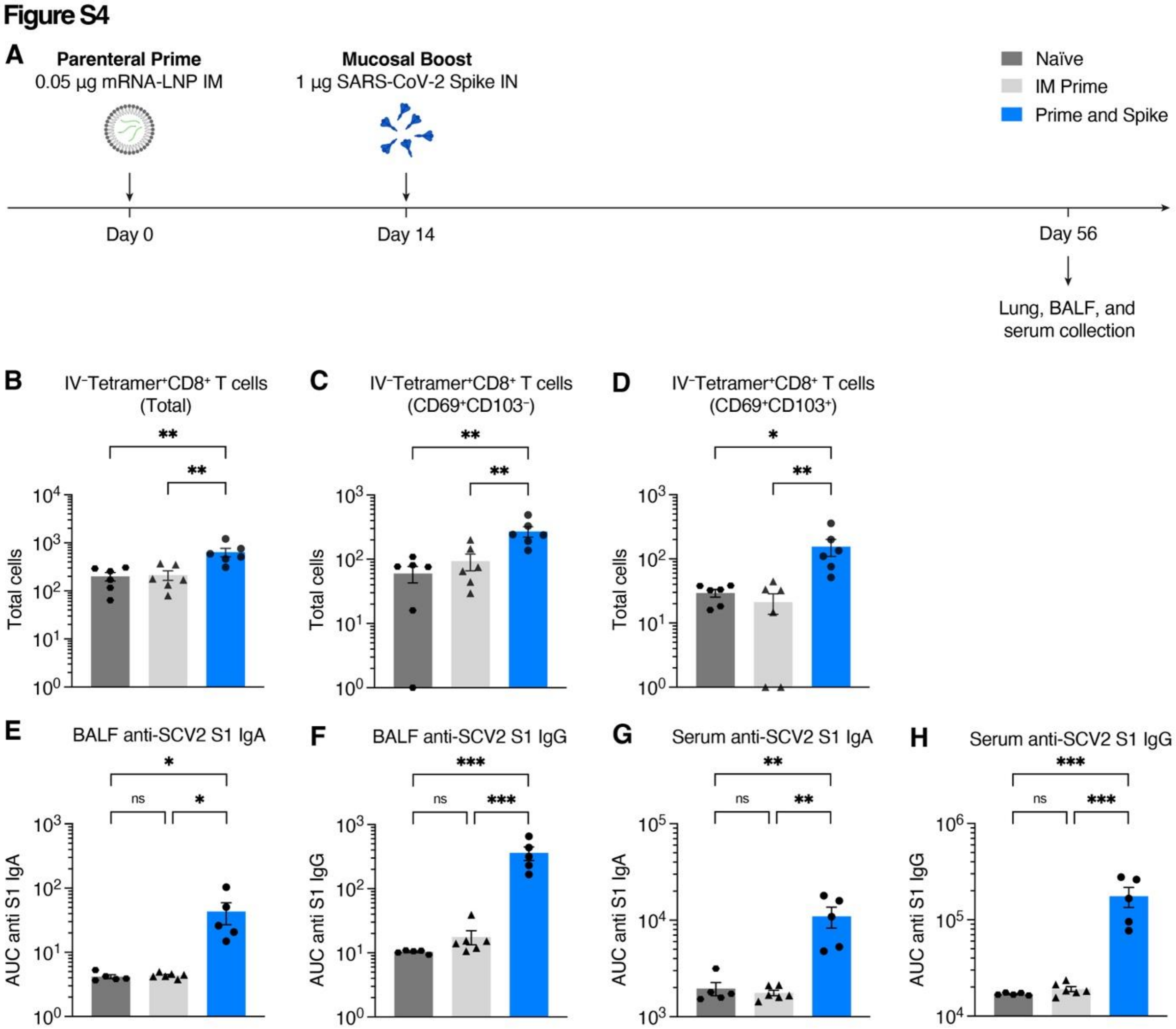

Fig S4. IN spike boost-induced protection against COVID-19 correlates with enhanced mucosal immunity. (**A**) Experimental schema: K18-hACE2 mice were IM primed with 0.05 µg of mRNA-LNP and IN boosted with 1 µg of spike IN 14 days post IM Prime. Six weeks post boost, lung tissues were collected for CD8 T cell analysis by flow cytometry, and BALF and blood were collected for antibody measurement. (**B to D**) Quantification of total tetramer^{+} CD8 T cells, CD69^{+}CD103^{-}tetramer^{+} CD8 T cells, or CD69^{+}CD103^{+}tetramer^{+} CD8 T cells in lung tissues from naïve, IM Prime, or P&S mice. (**E to H**) Measurement of SCV2 spike S1 subunit-specific BALF IgA (**E**), BALF IgG (**F**), serum IgA (**G**), and serum IgG (**H**) in naïve, IM Prime, or P&S mice. Mean ± s.e.m.; Statistical significance was calculated one-way ANOVA followed by Tukey's correction (**B to D**) or two-way ANOVA followed by Tukey's correction (**E to H**); *$P \leq 0.05$, **$P \leq 0.01$, ***$P \leq 0.001$, ****$P \leq 0.0001$. Individual data points are represented from one independent experiment.

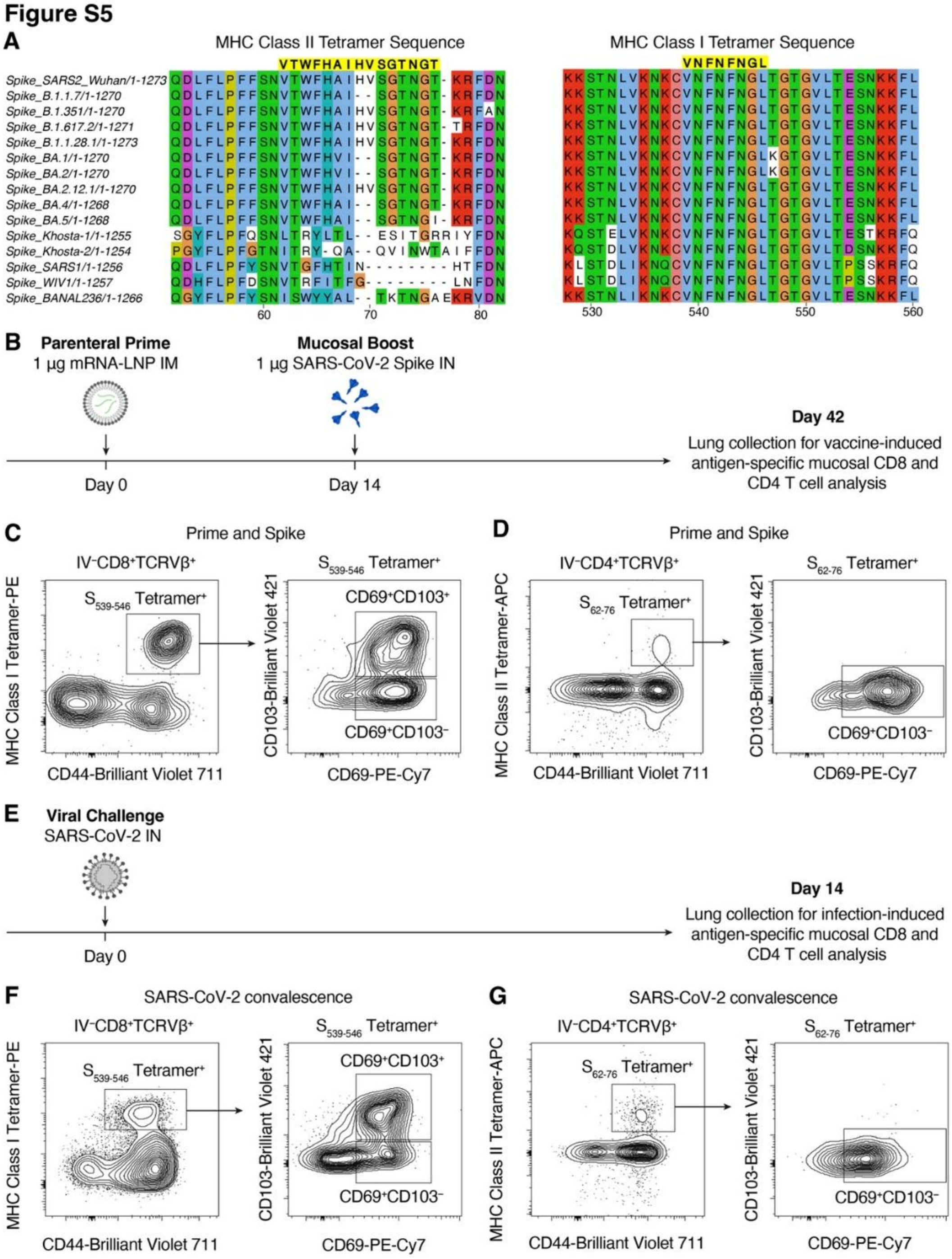

Fig S5. MHC-peptide tetramer-based identification of SARS-CoV-2 spike-specific CD8 and CD4 T cells. (**A**) Sequence alignment of CD4 epitope (VTWFHAIHVSGTNGT, $Spike_{62\text{-}76}$) and CD8 epitope (VNFNFNGL, $Spike_{539\text{-}546}$) from spike proteins of sarbecoviruses. (**B to D**) Identification and phenotypic analysis of spike-specific CD8 T cells (**C**) and CD4 T cells (**D**) in the lung 42 days post IM Prime and 28 days following IN spike boost. (**E to G**) Identification and phenotypic analysis of spike-specific CD8 T cells (**F**) and CD4 T cells (**G**) in the lung 14 days following SARS-CoV-2 infection. Contour plots (**C and F**) are representative of two independent experiments.

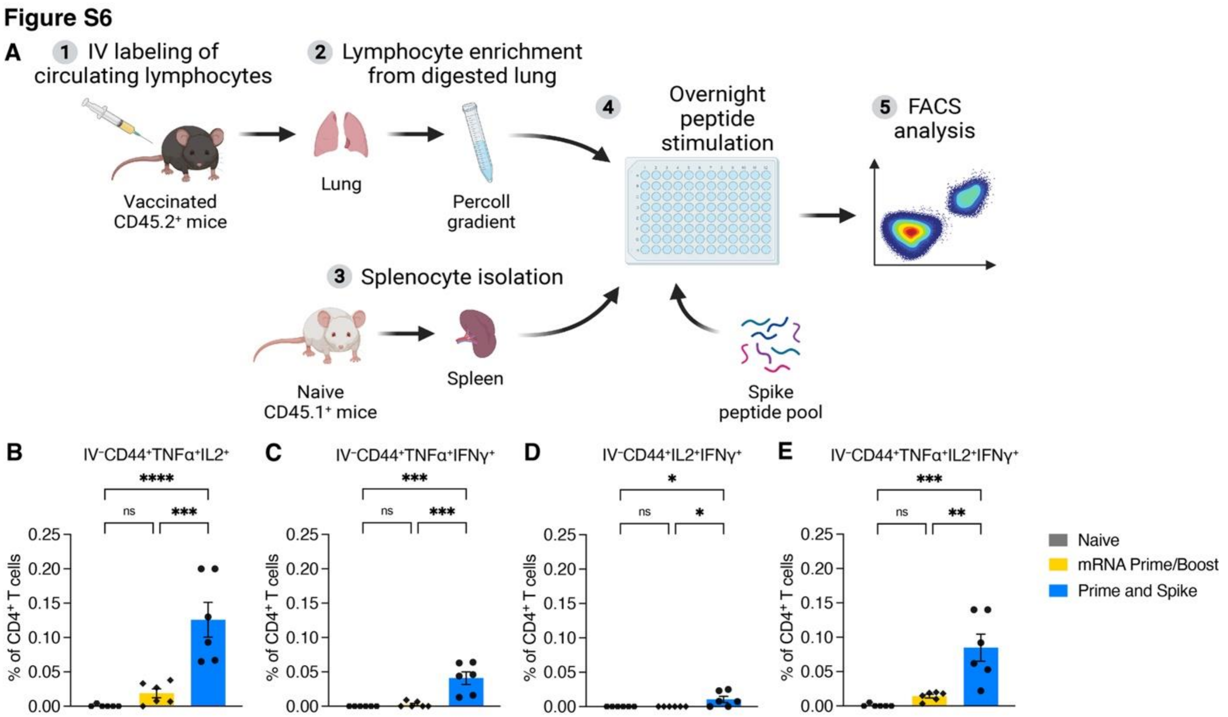

Fig S6. IN spike boost mediates expansion of polyfunctional spike-specific CD4 T cells in the lung parenchyma. (**A**) Experimental schema: K18-hACE2 mice were IM primed with 1 μg of mRNA-LNP and IN boosted with 1 μg of spike IN or IM boosted with 1 μg of mRNA-LNP 14 days post IM Prime. Forty-five days post prime, lung tissues were collected for assessment of antigen-specific $CD4^+$ T cell cytokine production by flow cytometry. Percoll gradient purified lung lymphocytes were restimulated with spike peptide megapool from SARS-CoV-2 and intracellular cytokine staining was performed to assess cytokine production by extravascular IV-$CD45^-CD4^+CD44^+$ T cells in naïve, mRNA-LNP prime–boost, or P&S mice. (**B to E**) Assessment of co-production of TNF-α and IL-2 (**B**), TNF-α and IFN-γ (**C**), IL-2 and IFN-γ (**D**), or TNF-α, IL-2, and IFN-γ (**E**). Mean ± s.e.m.; Statistical significance was calculated one-way ANOVA followed by Tukey's correction (**B to E**); *P≤0.05, **P≤0.01, ***P≤0.001, ****P≤0.0001. Individual data points are represented and are pooled from two independent experiments.

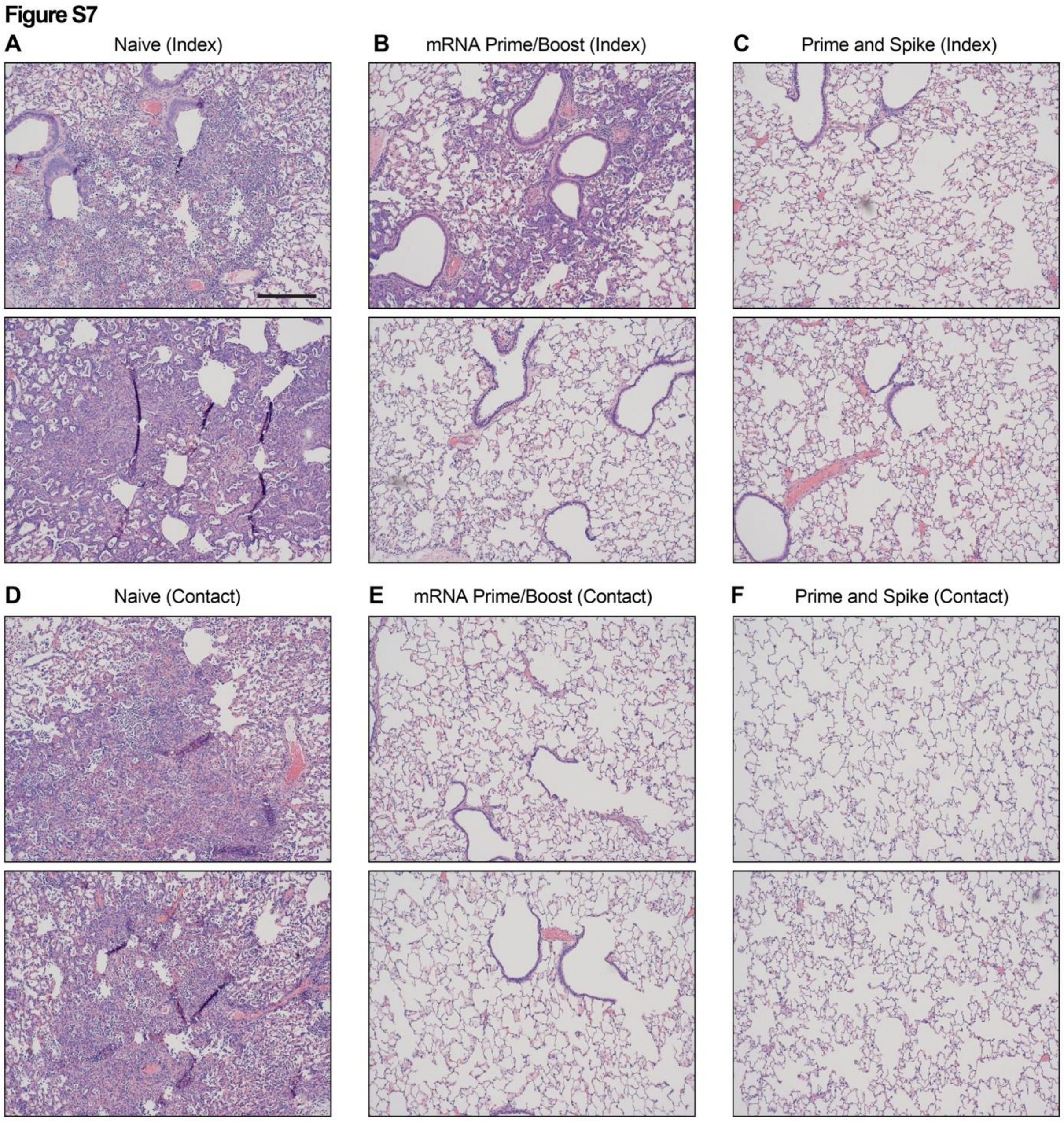

Fig S7. IN spike boost mediates disease protection in the lower respiratory tract in hamster model. (**A to C**) Experimental schema: Syrian hamsters were IM primed with 0.5 µg of mRNA-LNP, followed 21 days later by boosting with 0.5 µg of mRNA-LNP IM, or 5 µg of SCV2 spike IN. At 93 days post prime naïve, mRNA-LNP prime–boost, and P&S hamsters were infected with 6×10^3 PFU SARS-CoV-2. Lung tissues were collected for histopathologic analysis at 7 days post infection. Representative H&E staining results from naïve (**A**), mRNA prime–boost (**B**), or P&S (**C**) hamsters. **(D to F)** Experimental schema: Syrian hamsters vaccinated as above were cohoused for 4 hours with naïve donor hamsters who had been infected 24 hours earlier with 6×10^3 PFU SARS-CoV-2. Lung tissues were collected for histopathologic analysis at 7 days post viral exposure. Representative H&E staining results from naïve (**D**), mRNA prime–boost (**E**), or P&S (**F**) hamsters. Scale bar: 250 µm. Sections are representative of multiple sections from at least five hamsters per group.

Figure S8

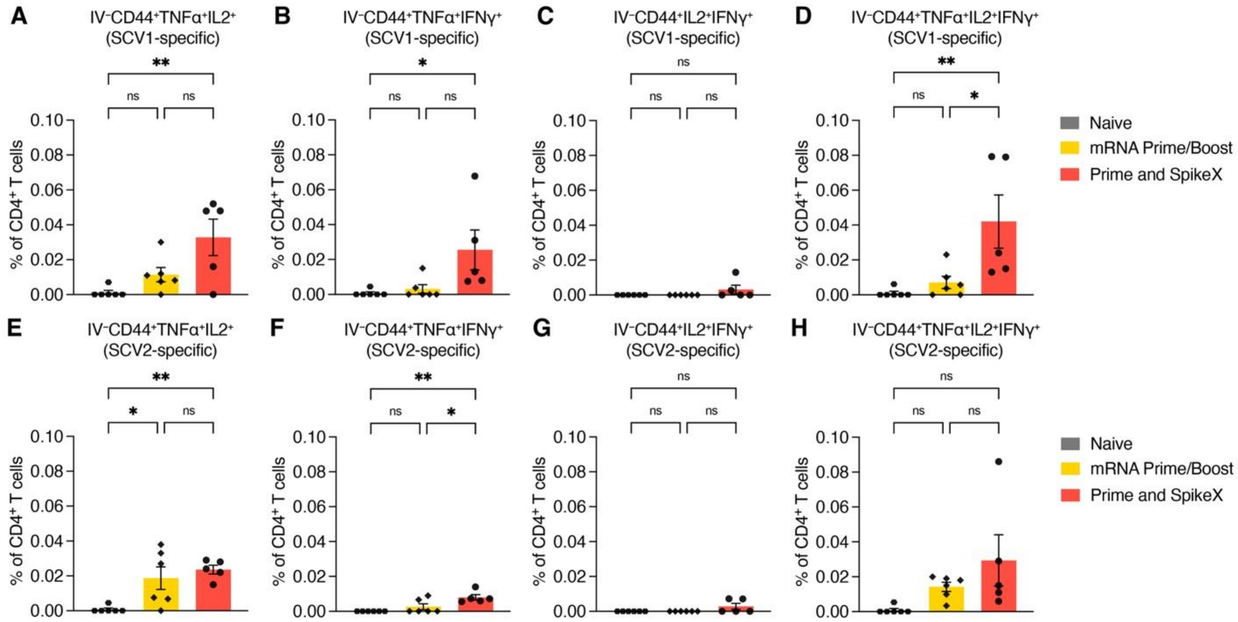

Fig S8. IN spike boost mediates expansion of polyfunctional spike-specific CD4 T cells in the lung parenchyma. (**A to H**) Experimental schema: K18-hACE2 mice were IM primed with 1 µg of mRNA-LNP and IN boosted with 5 µg of SARS-CoV-1 spike or IM boosted with 1 µg of mRNA-LNP 14 days post IM Prime. Forty-five days post prime, lung tissues were collected for assessment of antigen-specific CD4 T cell cytokine production by flow cytometry. Percoll gradient–purified lung lymphocytes were restimulated with spike peptide megapool from SARS-CoV-1 (**A to D**) or SARS-CoV-2 (**E to H**) and intracellular cytokine staining was performed to assess cytokine production by extravascular IV-$CD45^{-}CD44^{+}$ CD4 T cells in naïve, mRNA prime–boost, or P&Sx mice. (**A to D**) Assessment of co-production of TNF-α and IL-2 (**A**), TNF-α and IFN-γ (**B**), IL-2 and IFN-γ (**C**), or TNF-α, IL-2, and IFN-γ (**D**) by extravascular SARS-CoV-1 spike-specific $CD4^{+}$ T cells. (**E to H**) Assessment of co-production of TNF-α and IL-2 (**E**), TNF-α and IFN-γ (**F**), IL-2 and IFN-γ (**G**), or TNF-α, IL-2, and IFN-γ (**H**) by extravascular SARS-CoV-2 spike-specific $CD4^{+}$ T cells. Mean ± s.e.m.; Statistical significance was calculated one-way ANOVA followed by Tukey's correction (**A to H**); *$P \leq 0.05$, **$P \leq 0.01$, ***$P \leq 0.001$, ****$P \leq 0.0001$. Individual data points are represented and are pooled from two independent experiments.

Figure S9

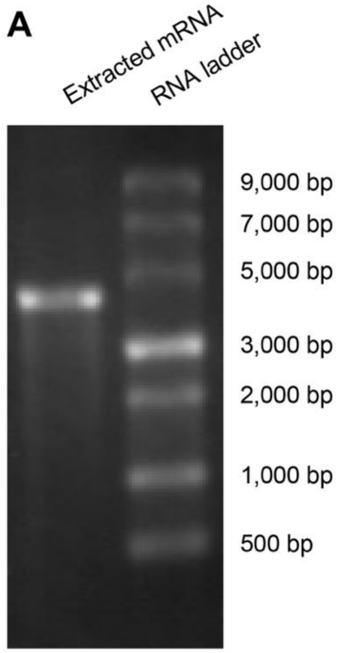

Fig S9. Confirmation of integrity of mRNA extracted from Comirnaty mRNA-LNP. (**A**) The length and integrity of extracted mRNA was analyzed using agarose gel electrophoresis. Extracted mRNA was mixed with SYBR Safe stain before being loaded onto a 1% agarose gel, let run in the TAE buffer, and imaged with a gel imaging system.

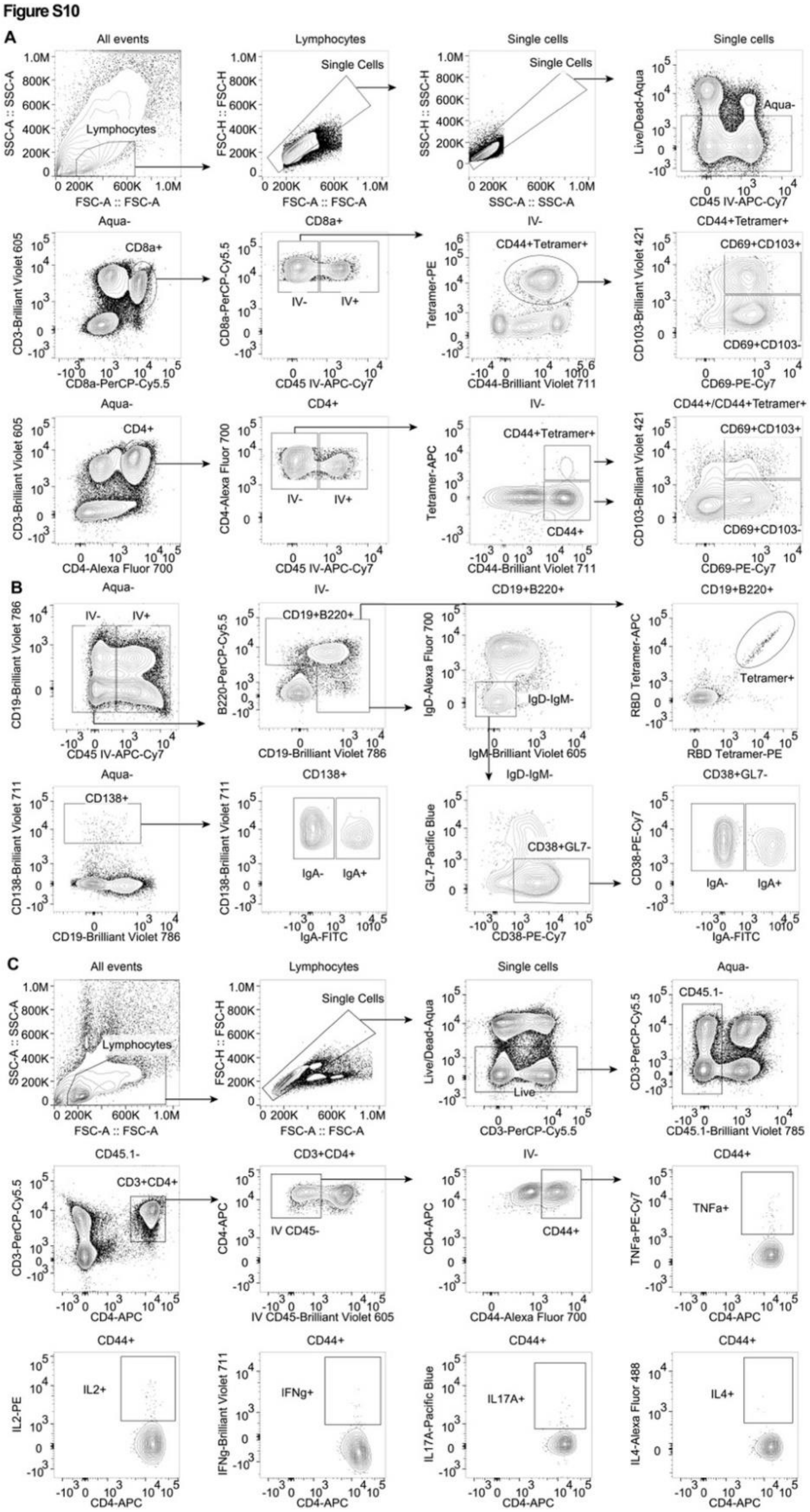

Fig S10. Gating strategies for analysis of extravascular adaptive immune responses in the respiratory tract. (**A**) Gating strategies to identify extravascular spike-specific CD8 and CD4 T cells. (**B**) Gating strategies to identify extravascular antigen-specific and polyclonal B cells. (**C**) Gating strategies to identify cytokine-producing extravascular spike-specific CD4 T cells.

Reagent	Fluorophore	Clone #	Cat #	Vendor	Final Conc.
					(µg/ml)
Anti-B220	PerCP/Cy5.5	RA3-6B2	103236	BioLegend	63.9
Anti-CD3	PerCP/Cy5.5	17A2	100218	BioLegend	36.1
Anti-CD3	BV605	17A2	100237	BioLegend	63.9
Anti-CD4	AF700	GK1.5	100430	BioLegend	159.7
Anti-CD4	AF647	GK1.5	100530	BioLegend	90.3
Anti-CD8a	PerCP/Cy5.5	53-6.7	100734	BioLegend	63.9
Anti-CD19	BV785	6D5	115543	BioLegend	5.4
Anti-CD38	PE/Cy7	90	102718	BioLegend	63.9
Anti-CD44	AF700	IM7	103026	BioLegend	159.7
Anti-CD44	BV711	IM7	103057	BioLegend	63.9
Anti-CD45.1	BV785	A20	110743	BioLegend	36.1
Anti-CD62L	FITC	MEL-14	104406	BioLegend	159.7
Anti-CD69	PE/Cy7	H1.2F3	104512	BioLegend	36.1
Anti-CD103	BV421	2E7	121422	BioLegend	36.1
Anti-CD138	BV711	281-2	142519	BioLegend	63.9
Anti-CXCR3	APC	CXCR3-173	126512	BioLegend	63.9
Anti-GL7	Pacific Blue	GL7	144614	BioLegend	159.7
Anti-IgA	FITC	N/A	1040-02	Southern Biotech	319.4
Anti-IgD	AF700	11-26c.2a	405730	BioLegend	159.7
Anti-IgM	BV605	RMM-1	406523	BioLegend	63.9
Anti-IL-2	PE	JES6-5H4	503808	BioLegend	36.1
Anti-IL-4	AF488	11B11	504109	BioLegend	90.3
Anti-IL-17A	BV421	TC11-18H10.1	506926	BioLegend	36.1
Anti-TNF-α	PE/Cy7	MP6-XT22	506324	BioLegend	36.1
Anti-IFN-γ	BV711	XMG1.2	505836	BioLegend	36.1
SCV2 Spike S62-76 MHC II tetramer	APC	N/A	N/A	NIH Tetramer Core	234.7
SCV2 Spike S539-546 MHC I tetramer	PE	N/A	N/A	NIH Tetramer Core	469.6
Anti-CD45	APC/Fire 750	30-F11	103154	BioLegend	2 µg/mouse
Anti-CD45	BV605	30-F11	103140	BioLegend	2 µg/mouse

Table S1. Specificities, conjugated fluorophores, clone numbers, catalogue numbers, vendors, dilutions, and final concentrations for flow cytometry reagents used in this study.
